# *Fmr1* Deletion and Early-Life Stress Interact to Increase Cell Proliferation and Glial Populations at the Expense of Immature Neurons in the Adult Dentate Gyrus

**DOI:** 10.3390/ijms27104356

**Published:** 2026-05-14

**Authors:** Sarah E. Latchney, Joan E. Ominuta, Lauryn E. L. Smitha, Katherine J. Blandin, Joaquin N. Lugo

**Affiliations:** 1 Department of Biology, St. Mary’s College of Maryland, St. Mary’s City, MD 20686, USA; 2 Department of Psychology and Neuroscience, Baylor University, Waco, TX 76709, USA

**Keywords:** Astrocyte, dentate gyrus, doublecortin, early-life stress, *Fmr1*, FMRP, hippocampus, Ki67, microglia, neurogenesis, proliferation

## Abstract

Fragile X Syndrome (FXS) is an inherited cause of intellectual disability and autism, arising from silencing of the *Fmr1* gene and loss of Fragile X Messenger Ribonucleoprotein 1 (FMRP). FMRP is an RNA-binding protein critically involved in neurodevelopmental processes, including neurogenesis. We examined the proliferation and maturation of adult-born dentate granule cells (abDGCs) and glial populations in *Fmr1* knockout (KO) and wild-type (WT) mice at 4, 12, and 24 weeks of age under control and early-life stress (ELS) conditions. Based on prior findings, we hypothesized that KO mice would exhibit increased neurogenesis and atypical responses to ELS compared with WT mice. Using immunohistochemistry, we quantified multiple stages of neurogenesis in the dentate gyrus, including proliferating (Ki67+), immature (doublecortin [DCX]+), and apoptotic (cleaved caspase-3 [CC3]+) cells. We also assessed glia using Iba1 (microglia) and GFAP (astrocytes) immunoreactivity. KO mice displayed significantly increased Ki67+ proliferating and reduced CC3+ apoptotic cells across ages, accompanied by increased Iba1+ and GFAP+ glial densities. However, KO mice exhibited fewer DCX+ neuroblasts at later time points. When reared in ELS conditions, KO mice show blunted or no changes in neurogenesis and glial populations relative to WT mice reared in ELS conditions or KO mice in control conditions. These results indicate that FMRP loss disrupts hippocampal neurogenesis by increasing cell proliferation while limiting neuronal maturation and expanding glial populations. Moreover, the absence of neurogenic and glial responses to ELS in KO mice highlights a gene–environment interaction that may influence FXS-related neuropathology by limiting the adaptive capacity of the hippocampal neurogenic niche.

## 1. Introduction

Fragile X syndrome (FXS) is an inherited form of intellectual disability and a leading genetic cause of autism spectrum disorder [1,2,3,4,5]. FXS results from transcriptional silencing of the Fragile X Messenger Ribonucleoprotein 1 (*FMR1*) gene and the consequent loss of its protein product, Fragile X Messenger Ribonucleoprotein (FMRP). FMRP is an RNA-binding protein that regulates mRNA transport, stability, and translation. It is highly expressed in the brain, where it plays important roles in synaptic plasticity, neuronal differentiation, and activity-dependent neurogenesis [6,7,8]. The *Fmr1* knockout (KO) mouse has served as the principal model of FXS to elucidate cellular and molecular mechanisms that contribute to the neurodevelopmental and cognitive deficits observed in the disorder.

Accumulating evidence indicates that FMRP also serves important regulatory functions in neural stem and progenitor cells, including adult-born dentate granule cells (abDGCs) residing in the subgranular zone (SGZ) of the hippocampal dentate gyrus [9,10,11,12]. Adult-born DGCs are critical for hippocampal plasticity, learning, and memory, and have been implicated in the etiology of several neuropsychiatric disorders [13,14,15,16,17]. Thus, alterations in the proliferation, maturation, or synaptic integration of abDGCs could contribute to hippocampal dysfunction in FXS [10,11,12,18]. To understand how loss of FMRP affects adult neurogenesis, earlier studies have examined cell proliferation and differentiation in the hippocampal SGZ of *Fmr1* KO mice using markers such as Ki67 and thymidine analogs (e.g., BrdU, CIdU) to quantify dividing and surviving cells and doublecortin (DCX) to label immature neurons. These studies reveal age-dependent effects on neurogenesis. In young adult mice (2 to 3 months old), *Fmr1* deletion is typically associated with increased cell proliferation [19,20], whereas in older adults (9 to 12 months old), proliferation and cell survival are reduced [21]. For example, Luo and colleagues [19] reported a ~52% increase in BrdU^+^ cells in young KO mice compared with wild-type (WT) controls, encompassing both radial glial cells and transient amplifying progenitors. Conditional deletion of *Fmr1* from neural stem cells in young mice similarly increased the number of proliferating glial fibrillary acidic protein (GFAP+) and S100β− radial glial cells and Ki67+DCX− transient amplifying neural progenitors while reducing the proportion of DCX+ neuroblasts and NeuN+ mature neurons [20]. Conversely, Lazarov et al. [21] demonstrated a significant reduction in both proliferating and surviving cells in older KO mice, leading to an overall decline in dentate granule cell numbers. Together, these early studies indicate that FMRP loss leads to age-dependent alterations in hippocampal neurogenesis and that FMRP acts cell-autonomously within abGGCs to regulate proliferation, cell lineage commitments, and behaviors dependent on hippocampal function [20].

Despite the documented disruptions in neurogenesis and glial populations in *Fmr1* KO mice, an important outstanding question is whether early-life stress (ELS) has additive effects in individuals with FXS. The average age of FXS diagnosis is approximately three years, suggesting that the early-life environment may interact with the underlying genetic vulnerability to shape long-term behavioral and cognitive outcomes [1,2]. The cognitive and behavioral phenotype of individuals with FXS includes reduced intellectual functioning, heightened anxiety, attention deficits, hyperactivity, and autism-related behaviors [1,2,3,4,5]. *Fmr1* KO mice also mirror some of these clinical observations. For example, KO mice have shown increased anxiety-like behavior and social anxiety in some studies [22,23] and have spatial learning and memory impairments in the Morris water maze task [24,25].

Physiologically, *Fmr1* KO mice exhibit a dampened corticosterone response to acute stress, despite normal basal corticosterone levels compared to WT littermates [26], suggesting dysregulation of the hypothalamic–pituitary–adrenal axis [27,28,29,30]. Although the decreased anxiety phenotype in KO mice appears inconsistent with the heightened anxiety seen in individuals with FXS, it may instead reflect atypical responsivity to stressful or arousing stimuli. Supporting this interpretation, KO mice exhibit impaired arousal regulation, which may underlie attention deficits and inhibitory control abnormalities [31,32]. Similarly, voluntary physical activity—a potent modulator of neurogenesis and an inherently arousing stimulus—attenuates neurogenic responses in KO mice. Pinar et al. [33] found that 28 days of voluntary running increased proliferation (Ki67+ cells) in the ventral dentate gyrus of WT mice but had no significant effect in KO mice. While BrdU+ cell survival improved in the dorsal dentate gyrus of KO mice after running, it remained unchanged ventrally, and DCX+ immature neuron counts were unaffected across conditions [33]. These results suggest that *Fmr1* loss compromises the hippocampus’s capacity to mount adaptive neurogenic responses to physiologically arousing experiences. Moreover, immediately following restraint stress, KO mice display a similarly blunted corticosterone response, with basal levels remaining unchanged [29], reinforcing the notion that the neural circuits mediating both stress- and arousal-related responses are functionally altered in the absence of FMRP [28,29,30].

Despite evidence of altered neurogenesis in KO mice, the temporal progression and regional specificity of neurogenic changes in these mice and the interaction with environmental factors such as ELS remain incompletely understood. Here, we examined abDGCs across subregions of the hippocampal dentate gyrus (SGZ, Granule Cell Layer [GCL], Hilus, and Molecular Layer) and glial cell populations (microglia and astrocytes) at 4, 12, and 24 weeks of age under baseline and ELS conditions. Based on prior evidence that *Fmr1* loss increases progenitor proliferation in young adults [19,20] but reduces proliferation and survival with aging [21], we hypothesized that KO mice would exhibit an early increase in proliferative activity followed by a decline in proliferative capacity at later stages, ultimately resulting in fewer postmitotic neurons in mature adults. We further predicted that neurogenic changes would accompany glial changes and that KO mice would exhibit an atypical neurogenic and glial response to ELS, reflecting a gene–environment (G × E) interaction that may contribute to hippocampal dysfunction and behavioral phenotypes with FXS.

## 2. Results

**Figure 1:**
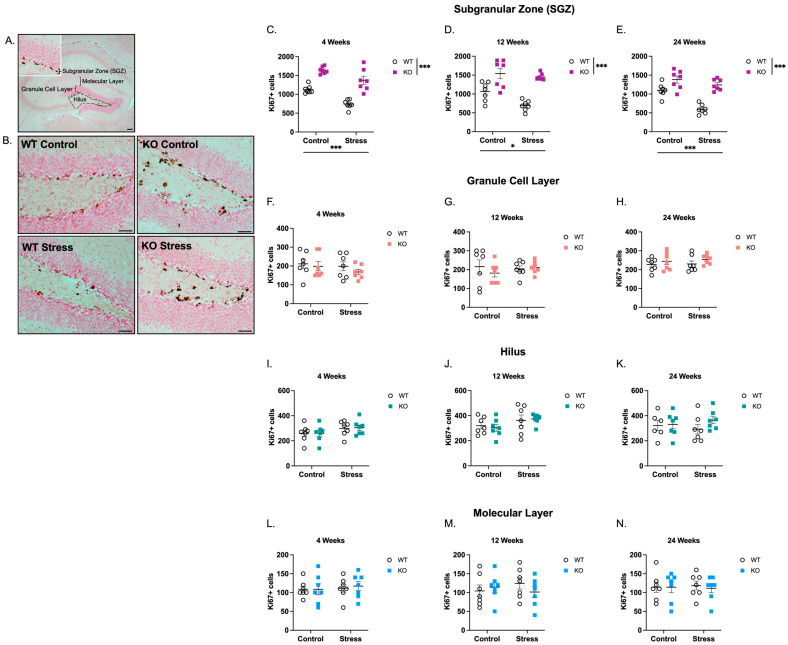
*Fmr1* KO mice and mice reared under ELS conditions have more Ki67-immunoreactive (Ki67+) proliferating cells in the SGZ at 4, 12, and 24 weeks of age. (**A**) Representative low-magnification image (40× total magnification; scale bar = 100 µm) of Ki67+ cells in the dentate gyrus, with dentate gyrus subregions labeled. (**B**) Representative higher-magnification images (200× total magnification; scale bar = 50 µm) of Ki67+ cells along the SGZ at 4 weeks of age. Ki67+ cells were counted using stereological methods in the SGZ ((**C**) 4 weeks, (**D**) 12 weeks, (**E**) 24 weeks), outer GCL ((**F**) 4 weeks, (**G**) 12 weeks, (**H**) 24 weeks), Hilus ((**I**) 4 weeks, (**J**) 12 weeks, (**K**) 24 weeks), and Molecular Layer ((**L**) 4 weeks, (**M**) 12 weeks, (**N**) 24 weeks. All data were analyzed by 2-way ANOVA (Genotype × Condition). Asterisks indicate significant main effects of genotype and rearing condition. Main effects of genotype were observed at 4 weeks (F(1,24) = 77.4, *p* < 0.0001), 12 weeks (F(1,24) = 47.4, *p* < 0.0001), and 24 weeks F(1,24) = 47.0, *p* < 0.0001). Main effects of ELS were observed at 4 weeks (F(1,24) = 25.0, *p* < 0.0001), 12 weeks (F(1,24) = 6.66, *p* < 0.05), and 24 weeks (F(1,24) = 22.6, *p* < 0.001). Because no genotype × condition interaction was detected, no post hoc analyses were performed. * *p* < 0.05, *** *p* < 0.001. N = 7 per group. Original microscopy images at full resolution are available as Supplementary Figure S1.

We assessed markers of neurogenesis and glial cell populations in the dentate gyrus at 4, 12, and 24 weeks of age, including the SGZ, GCL, Hilus, and Molecular Layer. A representative low-magnification image with all subregions labeled is shown in Figure 1A. With our study design, we tested three hypotheses: (1) compared to WT mice, *Fmr1* KO mice would display increased abDGC proliferation at early ages but decreased proliferative capacity and fewer adult-born post-mitotic neurons at later ages; (2) decreased neurogenesis in *Fmr1* KO mice would be associated with increased density of glial cells; and (3) *Fmr1* KO mice would demonstrate an atypical cellular response to early-life stressors, indicative of diminished capacity for adaptive plasticity towards stressful environments.

### 2.1. Proliferating Cells (Ki67) in Fmr1 KO Mice Under Control and ELS Conditions

The majority of Ki67+ cells were localized to the SGZ of both WT and KO mice, appearing as punctate nuclei lining the inner border of the granule cell layer (representative images of Ki67+ cells are shown in Figure 1A,B). In the SGZ, stereological quantification revealed a main effect of genotype where KO mice had a significant increase in Ki67+ cells compared to WT mice at 4 weeks (Figure 1C; 62% increase; F(1,24) = 77.4, *p* < 0.001), 12 weeks (Figure 1D; 71% increase; F(1,24) = 47.4, *p* < 0.001), and 24 weeks (Figure 1E; 55% increase; F(1,24) = 47.0, *p* < 0.001). There was also a main effect of ELS where ELS-exposed mice had a significant decrease in Ki67+ cells compared to mice reared in control conditions at 4 weeks (24% decrease; F(1,24) = 25.0, *p* < 0.001), 12 weeks (18% decrease; F(1,24) = 6.66, *p* < 0.05), and 24 weeks of age (25% decrease; F(1,24) = 22.6, *p* < 0.001). No genotype × ELS interaction was observed at any age; as such, no post hoc analyses are reported in Figure 1. When Ki67+ cells were quantified in the GCL (Figure 1F–H), Hilus (Figure 1I–K), or the Molecular Layer (Figure 1L–N), there were no effects of genotype, ELS, or interaction between the two variables at any age. These results suggest that KO mice have increased proliferative capacity in the SGZ compared with WT mice and that both *Fmr1* loss and ELS exposure were significant contributors to changes in abDGC proliferation.

**Figure 2:**
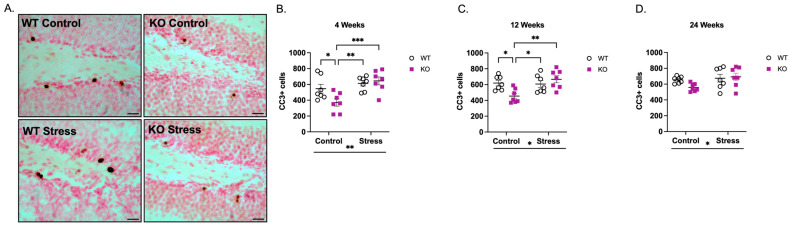
*Fmr1* KO mice reared under control conditions have reduced CC3-immunoreactive (CC3+) apoptotic cells at 4 and 12 weeks of age. (**A**) Representative images of CC3+ cells along the SGZ (200× magnification; scale bar = 50 µm). Stereological quantification of CC3+ cells at (**B**) 4 weeks, (**C**) 12 weeks, (**D**) 24 weeks revealed main effects of ELS, but not genotype, at all ages (4 weeks: F(1,24) = 13.0, *p* < 0.01; 12 weeks: F(1,24) = 6.78, *p* < 0.05; 24 weeks: F(1,24) = 4.49, *p* < 0.05). Significant genotype × ELS interactions were observed at 4 weeks (F(1,24) = 4.75, *p* < 0.05) and 12 weeks (F(1,24) = 8.41, *p* < 0.01), but not at 24 weeks. Data were analyzed by 2-way ANOVA (Genotype × Condition). Asterisks below the x-axis indicate significant main effects of rearing condition. Significant genotype × condition interactions were analyzed by Tukey’s multiple comparisons test. * *p* < 0.05, ** *p* < 0.01, *** *p* < 0.001. N = 7 per group. Original microscopy images at full resolution are available as Supplementary Figure S2.

While a surplus of abDGCs is typically produced, a subset of these undergo apoptosis to regulate neurogenesis [34,35]. The increase in cell proliferation observed in KO mice may be partially due to a decrease in apoptotic cell numbers. To investigate this, we quantified apoptosis in the SGZ using cleaved caspase-3 (CC3). As expected, CC3+ cells were evident in the dentate gyrus as punctate nuclei mainly in the SGZ (representative image of CC3+ cells in the SGZ is shown in Figure 2A). Because no changes in Ki67+ cells were observed in the GCL (Figure 1F–H), Hilus (Figure 1I–K), or Molecular Layer (Figure 1L–N), CC3+ cells were not quantified in these regions. In the SGZ, stereological quantification revealed a genotype × ELS interaction at 4 weeks of age (Figure 2B; F(1,24) = 4.75, *p* < 0.05). Follow-up analyses indicated a main effect of ELS (F(1,24) = 13.0, *p* < 0.01) but not genotype (F(1,24) = 2.29, *p* > 0.05), where ELS-exposed mice exhibited a 37% increase in CC3+ cells compared to mice reared in control conditions. *Post hoc* analysis revealed that in control conditions, KO mice had fewer CC3+ cells than WT mice (32% decrease; *p* < 0.05). However, ELS-exposed WT mice exhibited more apoptotic cells vs. KO mice in control conditions (*p* < 0.01), and ELS-exposed KO mice had more CC3+ cells vs. KO mice in control conditions (*p* < 0.001). There was no statistical significance between ELS-exposed WT and ELS-exposed KO mice, highlighting the effects of ELS—but not genotype—on apoptosis.

A similar genotype × ELS interaction was observed at 12 weeks of age (Figure 2C; F(1,24) = 8.41, *p* < 0.01), and this interaction was also driven by a main effect of ELS (F(1,24) = 6.78, *p* < 0.05) but not genotype (F(1,24) = 1.82, *p* > 0.05). ELS-exposed mice showed a 15% increase in CC3+ cells compared with mice reared in control conditions. *Post hoc* analysis revealed that in control mice, KO mice had fewer CC3+ cells compared to WT mice (26% decrease; *p* < 0.05), but ELS-exposed mice exhibited more apoptotic cells (ELS-exposed WT vs. KO control: 33% increase, *p* < 0.05; ELS-exposed KO vs. KO control: 47% increase, *p* < 0.01). No significant difference was observed between ELS-exposed WT and ELS-exposed KO mice.

At 24 weeks of age (Figure 2D), there was a main effect of ELS (F(1,24) = 4.49, *p* < 0.05), but no genotype × ELS interaction (F(1,24) = 2.46, *p* > 0.05). Accordingly, no *post hoc* results are reported. Across all ages, these results indicate that ELS exposure—but not genotype—was the primary driver of programmed cell death within the SGZ.

### 2.2. Neuroblasts (DCX) in Fmr1 KO Mice Under Control and Early-Life Stress Conditions

**Figure 3:**
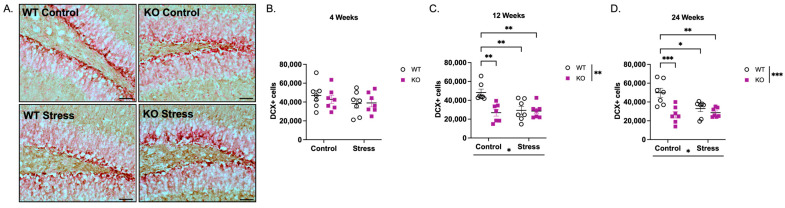
*Fmr1* KO mice and mice reared under ELS conditions have fewer DCX-immunoreactive (DCX+) immature neurons at 12 and 24 weeks of age. (**A**) Representative images of DCX+ cells in the SGZ (200× total magnification; scale bar = 50 µm). Stereological quantification of DCX+ cells at (**B**) 4 weeks, (**C**) 12 weeks, (**D**) 24 weeks. Main effects of genotype, ELS, and genotype × ELS interactions were observed at 12 weeks (Genotype: F(1,24) = 8.01, *p* < 0.01; ELS: F(1,24) = 6.01, *p* < 0.05; Genotype × ELS: F(1,24) = 8.62, *p* < 0.01) and 24 weeks (Genotype: F(1,24) = 16.0, *p* < 0.001; ELS: F(1,24) = 4.27, *p* < 0.05; Genotype × ELS: F(1,24) = 7.45, *p* < 0.05). Data were analyzed by 2-way ANOVA (Genotype × Condition). Asterisks indicate significant main effects of genotype and rearing condition. Significant genotype × condition interactions were analyzed by Tukey’s multiple comparisons test. * *p* < 0.05, ** *p* < 0.01, *** *p* < 0.001. N = 7 per group. Original microscopy images at full resolution are available as Supplementary Figure S3.

Doublecortin (DCX) is a microtubule-associated protein used to identify short-lived neuroblasts, in which the majority of cells have exited the cell cycle [36,37,38,39]. Because the cell bodies of DCX+ cells are concentrated in the SGZ and GCL (representative images of DCX+ cells are shown in Figure 3A), and since we did not observe proliferative changes outside the SGZ, we focused on DCX+ cell bodies located in the SGZ and GCL. Stereological quantification revealed an age-dependent change in the number of DCX+ cells. At 4 weeks (Figure 3B), there is no interaction between genotype and ELS (F(1,24) = 0.249, *p* > 0.05) nor a main effect of genotype (F(1,24) = 0.139, *p* > 0.05) or ELS (F(1,24) = 1.75, *p* > 0.05). However, at 12 weeks (Figure 3C), there is a genotype × ELS interaction (F(1,24) = 8.62, *p* < 0.01) and main effects of genotype (29% decrease in KO vs. WT; F(1,24) = 8.01, *p* < 0.01) and ELS condition (23% decrease in ELS vs. Control; F(1,24) = 6.01, *p* < 0.05). At 24 weeks (Figure 3D), there was also a genotype × ELS interaction (F(1,24) = 7.45, *p* < 0.05) and main effects of genotype (34% decrease in KO vs. WT; F(1,24) = 16.0, *p* < 0.001) and ELS condition (18% decrease in ELS vs. Control; F(1,24) = 4.27, *p* < 0.05). *Post hoc* analysis revealed a significant decrease in KO vs. WT mice reared in control conditions at 12 weeks (45% decrease; *p* < 0.01) and 24 weeks (11% decrease; *p* < 0.001). Mice of both genotypes reared in ELS conditions also had statistically fewer DCX+ cells compared to WT mice reared in control conditions, but no significance was observed between ELS-exposed WT and KO mice (*p* > 0.05), underscoring the contribution of *Fmr1* loss—rather than ELS exposure—to changes in DCX+ cell number.

### 2.3. Glial Populations in Fmr1 KO Mice Under Control and Early-Life Stress Conditions

**Figure 4:**
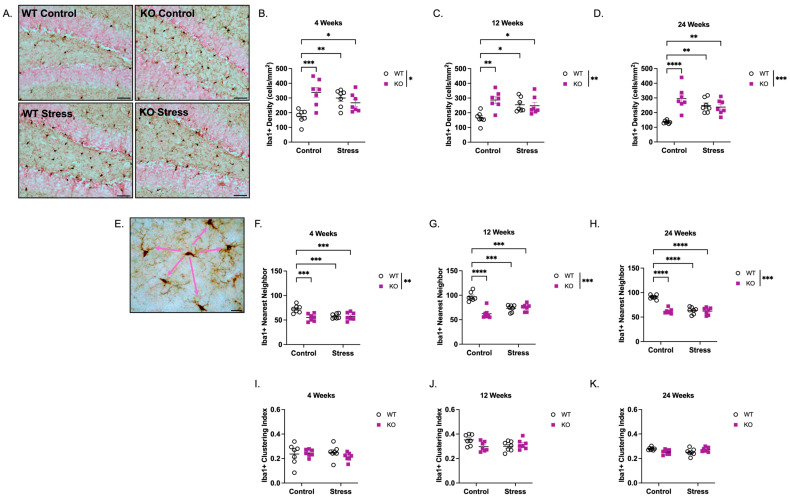
*Fmr1* KO mice have increased Iba1-immunoreactive (Iba1+) microglia in the dentate gyrus at 4, 12, and 24 weeks of age. (**A**) Representative images of Iba1+ cells in the dentate gyrus (200× total magnification; scale bar = 50 µm). Density measurements of Iba1+ cells in the dentate gyrus at (**B**) 4 weeks, (**C**) 12 weeks, (**D**) 24 weeks. A main effect of genotype (4 weeks: F(1,24) = 6.77, *p* < 0.05; 12 weeks: F(1,24) = 8.12, *p* < 0.01; 24 weeks: F(1,24) = 14.0, *p* < 0.001) and genotype × ELS interactions (4 weeks: F(1,24) = 15.2, *p* < 0.001; 12 weeks: F(1,24) = 10.1, *p* < 0.01; 24 weeks: F(1,24) = 18.2, *p* < 0.001) were observed. (**E**) Representative image of Iba1+ cells to show the concept of “nearest neighbor” and “spacing index.” Pink arrows represent distances of an Iba1+ cell to its “nearest neighbors;” values are averaged and normalized for density to obtain the spacing (clustering) index. Scale bar = 20 µm. Smaller nearest neighbor values indicate shorter average distances. Nearest neighbor measurements at (**F**) 4 weeks, (**G**) 12 weeks, (**H**) 24 weeks. Main effect of genotype was observed at 4 weeks (F(1,24) = 9.85, *p* < 0.01), 12 weeks (F(1,24) = 26.4, *p* < 0.001), and 24 weeks (F(1,24) = 43.3, *p* < 0.001). Significant genotype × ELS interactions were also observed at 4 weeks (F(1,24) = 9.85, *p* < 0.01), 12 weeks (F(1,24) = 31.5, *p* < 0.0001), and 24 weeks (F(1,24) = 35.4, *p* < 0.0001). Spacing (clustering) index measurements at (**I**) 4 weeks, (**J**) 12 weeks, and (**K**) 24 weeks revealed no significant effects of genotype, ELS, or genotype × ELS interaction. Data were analyzed by 2-way ANOVA (Genotype × Condition), and asterisks indicate significant main effects of genotype. Significant genotype × condition interactions were analyzed by Tukey’s multiple comparisons test. * *p* < 0.05, ** *p* < 0.01, *** *p* < 0.001, **** *p* < 0.0001. N = 7 per group. Original microscopy images at full resolution are available as Supplementary Figure S4.

Microglia are significant contributors to maintaining neural stem cell/progenitor numbers in the developing cortex [40] and hippocampus [41], and their density increases throughout the brain during development [42]. The hippocampus of ELS-exposed adult mice also exhibits changes in microglial morphology and transcriptomics [43]. Therefore, we quantified the density and spatial distribution of ionized calcium-binding adaptor molecule 1 (Iba1)+ microglia in the dentate gyrus. We hypothesized that KO mice would exhibit increased microglial density, paralleling the increased cell proliferation observed at early ages, an association we have previously reported [44]. Because we only observed statistical changes in neurogenesis in the SGZ, and microglia are abundant throughout the hippocampal dentate gyrus, we quantified Iba1+ cells throughout the entire dentate gyrus as a single measurement (representative images of Iba1+ cells are shown in Figure 4A). Iba1+ density counts revealed a genotype × ELS interaction at 4 weeks (Figure 4B; F(1,24) = 15.2, *p* < 0.001), 12 weeks (Figure 4C; F(1,24) = 10.1, *p* < 0.01), and 24 weeks (Figure 4D; F(1,24) = 18.2, *p* < 0.001) and main effects of genotype at 4 weeks (F(1,24) = 6.77, *p* < 0.05), 12 weeks (F(1,24) = 8.12, *p* < 0.01), and 24 weeks (F(1,24) = 14.0, *p* < 0.001). There was no main effect of ELS at any age. *Post hoc* analysis revealed that KO mice had a significant increase in Iba1+ density compared to WT mice at 4 weeks (93% increase; *p* < 0.001), 12 weeks (74% increase; *p* < 0.01), and 24 weeks (117% increase; *p* < 0.001) in the absence of ELS exposure. ELS-exposed mice of both genotypes also exhibited significantly higher Iba1+ density vs. WT control mice at all ages. However, in the presence of ELS, there was no statistical difference in Iba1+ density between WT and KO mice at any age, underscoring *Fmr1* loss to be the main contributor to increased microglial density in the dentate gyrus.

Using the density data, we calculated the nearest neighbor distance to measure the proximity of cell bodies within the dentate gyrus. Because this measurement is based on the shortest distance to a cell’s nearest neighbor (see description in Methods; Figure 4E), decreases in these values indicate that cells are spaced closer together, reflecting a higher cell density. Supporting Figure 4B–D, there was a main effect of genotype for Iba1+ nearest neighbor distance at 4 weeks (Figure 4F; F(1,24) = 9.85, *p* < 0.01), 12 weeks (Figure 4G; F(1,24) = 26.4, *p* < 0.001), and 24 weeks (Figure 4H; F(1,24) = 43.3, *p* < 0.001). KO mice reared in control conditions displayed a 22% decrease at 4 weeks (*p* < 0.001), 36% decrease at 12 weeks (*p* < 0.001), and 32% decrease at 24 weeks (*p* < 0.001) in nearest neighbor distance compared to WT control mice. These genotype effects contributed to a significant genotype × ELS interaction at 4 weeks (F(1,24) = 9.85, *p* < 0.01), 12 weeks (F(1,24) = 31.5, *p* < 0.001), and 24 weeks (F(1,24) = 35.4, *p* < 0.001). However, similar to the density measurements, there were no main effects of ELS at any age. *Post hoc* analysis revealed that KO mice reared in control conditions exhibited decreases of 13% (4 weeks), 29% (12 weeks), and 35% (24 weeks) in Iba1+ cell distance compared to WT controls. However, as with Iba1+ density, while ELS-exposed WT and KO mice had decreased spacing between cell bodies, there was no statistical difference between ELS-exposed WT and ELS-exposed KO mice at any age.

Nearest neighbor measurements—when corrected for density (see description in Methods)—can also be used to assess the spacing regularity of cell bodies, referred to as the spacing or clustering index (Figure 4I–K). There were no main effects of genotype, ELS, or genotype × ELS interaction in the Iba1+ clustering index among any group, suggesting that even though KO mice reared in control conditions and ELS-exposed mice had a higher microglia density (Figure 4B–D) and decreased spacing between cell bodies (Figure 4F–H), the regularity of their spacing patterns throughout the dentate gyrus did not differ (Figure 4I–K).

**Figure 5:**
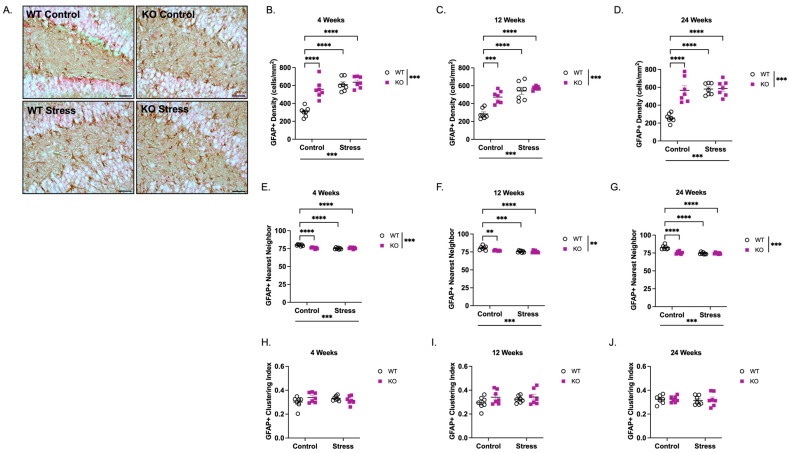
*Fmr1* KO mice have increased GFAP-immunoreactive (GFAP +) astrocytes in the dentate gyrus at 4, 12, and 24 weeks of age. (**A**) Representative images of GFAP + cells in the dentate gyrus (200× total magnification; scale bar = 50 µm). Density measurements of GFAP + cells in the dentate gyrus at (**B**) 4 weeks, (**C**) 12 weeks, (**D**) 24 weeks. At 4 weeks, there were main effects of genotype (F(1,24) = 23.2, *p* < 0.001), ELS (F(1,24) = 44.6, *p* < 0.001), and a genotype × ELS interaction (F(1,24) = 15.7, *p* < 0.001). Similar effects were observed at 12 weeks (Genotype: F(1,24) = 18.1, *p* < 0.001; ELS: F(1,24) = 47.7, *p* < 0.001; genotype × ELS: F(1,24) = 9.08, *p* < 0.01) and 24 weeks (Genotype: F(1,24) = 21.5, *p* < 0.001; ELS: F(1,24) = 25.4, *p* < 0.001; genotype × ELS: F(1,24) = 19.4, *p* < 0.001). Nearest neighbor measurements at (**E**) 4 weeks, (**F**) 12 weeks, (**G**) 24 weeks. At 4 weeks, there were main effects of genotype (F(1,14) = 14.9, *p* < 0.001), ELS (F(1,24) = 34.1, *p* < 0.001), and a genotype × ELS interaction (F(1,24) = 29.7, *p* < 0.001). Similar effects were observed at 12 weeks (Genotype: F(1,24) = 8.26, *p* < 0.01; ELS: F(1,24) = 24.0, *p* < 0.001; genotype × ELS: F(1,24) = 5.52, *p* < 0.05) and 24 weeks (Genotype: F(1,24) = 23.9, *p* < 0.001; ELS: F(1,24) = 38.5, *p* < 0.001; genotype × ELS: F(1,24) = 26.0, *p* < 0.001). Spacing (clustering) index measurements at (**H**) 4 weeks, (**I**) 12 weeks, and (**J**) 24 weeks. Data were analyzed by 2-way ANOVA (Genotype × Condition), and asterisks indicate significant main effects of genotype and rearing condition. Significant genotype × condition interactions were analyzed by Tukey’s multiple comparisons test. ** *p* < 0.01, *** *p* < 0.001, **** *p* < 0.0001. N = 7 per group. Original microscopy images at full resolution are available as Supplementary Figure S5.

Astrocytes are another glial population affected in many neurodevelopmental disorders, including FXS [45,46,47,48,49,50]. To this end, we also quantified the density and spacing of Glial Fibrillary Acidic Protein (GFAP)+ astrocytes using the same methods as Iba1 (representative images of GFAP+ cells are shown in Figure 5A). There was a main effect of genotype (F(1,24) = 23.2, *p* < 0.001), ELS (F(1,24) = 44.6, *p* < 0.001), and a genotype × ELS interaction (F(1,24) = 15.7, *p* < 0.001) at 4 weeks of age (Figure 5B). *Post hoc* analysis revealed a significant increase in GFAP+ density in the WT control group vs. KO control (82% increase; *p* < 0.0001), WT ELS (106% increase; *p* < 0.0001) and ELS-exposed KO (108% increase; *p* < 0.0001). Similar effects were observed at 12 weeks (Figure 5C; Genotype: F(1,24) = 18.1, *p* < 0.001; ELS: F(1,24) = 47.7, *p* < 0.001; genotype × ELS: F(1,24) = 9.08, *p* < 0.01) and 24 weeks (Figure 5D; Genotype: F(1,24) = 21.5, *p* < 0.001; ELS: F(1,24) = 25.4, *p* < 0.001; genotype × ELS: F(1,24) = 19.4, *p* < 0.001). *Post hoc* analysis also revealed similar significant increases in GFAP + density in the WT control vs. KO control (12 weeks: 66% increase, *p* < 0.0001; 24 weeks: 119% increase, *p* < 0.0001), ELS-exposed WT (12 weeks: 90% increase, *p* < 0.0001; 24 weeks: 125% increase, *p* < 0.0001), and ELS-exposed KO (12 weeks: 101% increase, *p* < 0.0001; 24 weeks: 128% increase, *p* < 0.0001) groups. However, in the presence of ELS, there was no statistical difference in GFAP + density between WT and KO mice at any age.

When GFAP + nearest neighbor distance was measured, there was a main effect of genotype at 4 weeks (Figure 5E; F(1,14) = 14.9, *p* < 0.001), 12 weeks (Figure 5F; F(1,24) = 8.26, *p* < 0.01), and 24 weeks (Figure 5G; F(1,24) = 23.9, *p* < 0.001) and a main effect of ELS at 4 weeks (F(1,24) = 34.1, *p* < 0.001), 12 weeks (F(1,24) = 24.0, *p* < 0.001), and 24 weeks (F(1,24) = 38.5, *p* < 0.001). These effects contributed to a significant genotype × ELS interaction at 4 weeks (F(1,24) = 29.7, *p* < 0.001), 12 weeks (F(1,24) = 5.52, *p* < 0.05), and 24 weeks (F(1,24) = 26.0, *p* < 0.001). *Post hoc* analysis revealed consistent statistical decreases in nearest neighbor distances in WT mice reared in control conditions vs. KO mice reared in control conditions, ELS-exposed WT, and ELS-exposed KO groups at 4, 12, and 24 weeks. However, in the presence of ELS, there was no statistical difference between WT and KO mice at any age.

When the clustering index for GFAP+ cells was calculated, there were no main effects of genotype, ELS, or genotype × ELS interaction among any group, suggesting that even though KO mice raised in control conditions and mice exposed to ELS had a higher density of astrocytes (Figure 5B–D) and decreased spacing between cell bodies (Figure 5E–G), the regularity of their spacing patterns throughout the dentate gyrus did not differ (Figure 5H–J).

## 3. Discussion

In this study, we assessed whether *Fmr1* KO mice show alterations in the proliferation and differentiation of adult-born dentate granule cells (abDGCs), a phenotype previously reported [19,20,21], and whether these changes interact with ELS exposure in a mouse model of FXS. We hypothesized that KO mice would exhibit increased abDGC proliferation at early ages but reduced proliferative capacity and fewer mature neurons later in life, accompanied by a greater density of glial cells, replicating established findings. We further hypothesized that KO mice would exhibit atypical cellular responses to early-life stressors—a previously untested aspect of *Fmr1*-related neurogenesis—which could be considered a maladaptive form of neuroplasticity. In aggregate, our results show dynamic and complex influences of genetic vulnerability to FXS, ELS exposure, and developmental age on abDGCs and glial populations, highlighting an important interaction between genetic risk and early-life environmental influences.

Consistent with earlier studies [19,20,21], KO mice showed increased numbers of proliferating abDGCs in the dentate gyrus SGZ starting at an early age (4 weeks) and persisting throughout adulthood (Figure 1), but fewer immature neurons labeled with DCX (Figure 3). The consistent increase in proliferating abDGCs in KO mice across ages, coupled with reduced numbers of immature neurons, has been documented by others and points to a sustained imbalance between cell division and neuronal differentiation [19,20,21]. In young adults, proliferation is often increased without consistent gains in survival or neuronal output [19,20,21]. In older adults, both proliferation and survival are markedly reduced, resulting in fewer immature (DCX+) and mature (NeuN+) neurons, despite a maintained or elevated fraction of neuronal differentiation among surviving cells [21]. The combination of the increased proliferation and reduced apoptosis observed in the current study suggests an imbalance in neurogenesis, in which increased cell survival does not translate into effective neuronal differentiation or maturation. Developmental timing also appears to shape how *Fmr1* loss impacts abDGCs and glial cells. The early increase in abDGC proliferation without a corresponding increase in DCX+ immature neurons suggests that FMRP normally constrains proliferation and promotes timely differentiation during the early postnatal period. Over time, chronic dysregulation may deplete the abDGC pool or disrupt niche signaling, contributing to the reduced neurogenesis observed at later ages. While speculative, these developmental shifts could have important implications for the emergence of cognitive and affective symptoms in FXS, which often arise during childhood and adolescence, periods when hippocampal remodeling and experience-dependent plasticity are particularly active.

Increased proliferation was also associated with fewer apoptotic cells (Figure 2) and higher glial density (Figure 4 and Figure 5), suggesting altered cell survival and lineage specification. FMRP regulates the translation of mRNAs involved in signaling cascades such as Notch, Wnt, and PI3K–mTOR [6,51], which are critical for neural stem cell maintenance and fate specification. Dysregulation of these pathways could bias abDGCs toward continued self-renewal or glial differentiation, consistent with our observation of increased microglial and astrocytic densities. Similar cellular phenotypes have been observed in human iPSC-derived neural progenitors lacking FMRP [45] and in conditional *Fmr1* deletion models [19,20], suggesting that disrupted translational control within abDGCs is a core cellular feature of FXS. When considered together, these findings suggest that the reduction in immature neurons in *Fmr1* KO mice reflects impaired neuronal maturation, driven by reduced survival, increased apoptosis, and/or altered cell-fate specification within the neurogenic niche.

The elevation in Iba1+ microglia density in KO mice reared in control conditions (Figure 4) may further reflect an altered neuroimmune environment that contributes to an atypical cellular milieu within the dentate gyrus. Microglia regulate the survival, differentiation, and integration of abDGC through both trophic and pruning mechanisms [35,41,52,53]. Hyperproliferation or prolonged activation of microglia can impair neurogenesis, synaptic remodeling, and cognitive function [54,55,56]. Recent evidence indicates that FMRP deficiency intrinsically alters microglial morphology, phagocytic activity, and responsiveness to sensory experience [26,57,58], suggesting that the elevated Iba1+ density in KO mice reflects a disruption of microglial homeostasis rather than a uniform proinflammatory activation. Supporting this view, postmortem analyses of individuals with FXS reveal elevated inflammatory markers and abnormal microglial morphology, paralleling findings in related neurodevelopmental disorders such as autism spectrum disorder [47,50,59,60].

While it was unexpected that microglial density was not different in KO mice reared in ELS conditions relative to those reared in control conditions, it may represent a compensatory or homeostatic response mediated by altered glucocorticoid signaling or dampened hypothalamic–pituitary–adrenal (HPA) axis reactivity in the absence of FMRP [26,28,29]. Reduced corticosterone responsivity in *Fmr1* KO mice could attenuate stress-induced neuroinflammatory cascades that typically heighten microglial activation, thereby blunting ELS-induced effects on glial morphology and density [43,61,62]. This pattern aligns with the broader hypothesis that loss of FMRP confers reduced sensitivity to environmental perturbation, manifesting as both a buffering effect under stress and a diminished capacity for adaptive plasticity under stimulating conditions [33].

Astrocytic alterations paralleled microglial changes, where we observed elevated GFAP+ density in KO mice reared under control conditions that was not further increased by ELS (Figure 5). FMRP is expressed in astrocytes, and these cells play central roles in neuronal maturation, synaptogenesis, and modulating the neurogenic niche through the release of trophic factors, metabolic support, and cytokine signaling [47,63,64,65]. In FXS models, astrocyte-specific loss of FMRP alters astrocyte-neuron signaling and delays dendritic maturation and synaptic protein expression [65,66], and recent reviews emphasize the contribution of glial dysfunction to FXS pathophysiology [47,48,50]. Increased GFAP+ density in KO mice may reflect altered astrocyte differentiation, cell lineage commitments, reactive-like states, or compensatory glial proliferation that disrupts support for abDGCs. Such astrocytic dysregulation can impair neuronal maturation and circuit refinement. Importantly, astrocytes express glucocorticoid receptors and are direct targets of stress hormones. Glucocorticoids and chronic stress alter astrocyte morphology, glutamate and energy metabolism, and cytokine release—mechanisms that can modulate neurogenesis and synaptic stability [67,68]. Thus, the persistence of elevated GFAP+ density in *Fmr1* KO mice, regardless of early-life condition, may reflect intrinsic FMRP-dependent astrocyte dysregulation that occludes further stress-induced remodeling, or alternatively, a blunted astrocytic endocrine responsiveness. Human and translational studies also point to astrocytic perturbations in FMR1-related conditions [46,47], supporting the clinical relevance of these cellular phenotypes. Together, it is conceivable that these microglial and astrocytic alterations contribute to FXS neuropathology by disrupting the neurogenic niche, impairing neuronal maturation and synaptic integration, and ultimately altering hippocampal circuit function.

The ELS paradigm used in our current study is the limited bedding and nesting (LBN) model, an established model of early postnatal stress that disrupts maternal care during a sensitive period of hippocampal development [69,70]. The developmental window during which mice were reared under control or LBN conditions (P2–P9) corresponded to the third trimester of human gestation [71,72,73]. This paradigm reliably induces both immediate and enduring alterations in HPA axis function, neural structure, and behavior. For example, Rice and colleagues [70] demonstrated that LBN exposure during postnatal days 2 to 9 elevates basal plasma corticosterone levels, blunts stress-induced corticosterone release, and impairs hippocampal-dependent spatial learning and memory in adulthood [70]. Similarly, Naninck et al. [69] demonstrated that early LBN exposure reduces cell proliferation and the number of DCX+ immature neurons in the dentate gyrus, accompanied by increased anxiety-like behavior and decreased hippocampal glucocorticoid receptor expression in adult mice [69]. These studies establish the LBN model as a paradigm for producing lasting neuroendocrine and cognitive consequences of ELS through hippocampal circuit dysregulation and impaired neurogenesis.

In this context, our findings provide insight into how genetic vulnerability to FXS may interact with ELS within a broader gene–environment (G × E) framework in neurodevelopmental disorders. Our results show that KO mice may respond to ELS differently than WT mice, suggesting that they may be buffered to the adverse effects of ELS environments or have diminished adaptive capacity. Across all ages, KO mice in ELS conditions largely did not differ from KO mice in control conditions or from WT mice in ELS conditions for all cell populations measured (DCX+, Iba1+, and GFAP+), with the exception of actively dividing Ki67+ cells. Ki67+ cells could be uniquely susceptible to ELS, as proliferating progenitors are particularly vulnerable to glucocorticoid- and stress-induced influences that impair survival and maturation [74], and suppression of adult neurogenesis has been shown to slow HPA axis recovery following stress [75]. Classic studies have similarly demonstrated that psychosocial stress and ELS suppress hippocampal neurogenesis [76,77,78,79]. Structural plasticity within the hippocampus—including both neurogenic and glial adaptations—represents a key mechanism through which stress and experience shape cognitive and emotional function [80]. Thus, the increased proliferation of Ki67+ cells in *Fmr1* KO mice may likely fail to produce functionally integrated neurons, with potential negative consequences for hippocampal-dependent learning and memory. Together, these results suggest that while ELS exerts long-lasting effects on hippocampal neurogenesis in WT animals [69,70], the absence of FMRP disrupts this relationship, leading to a muted or atypical cellular response to early stress. In contrast to canonical G × E models in which environmental stress amplifies underlying vulnerability, this divergence from canonical LBN outcomes implies that FMRP serves as a molecular modulator of stress-responsive signaling within the hippocampal neurogenic niche—potentially by regulating the translation of mRNAs involved in glucocorticoid receptor signaling, neurotrophic pathways, and synaptic remodeling—such that its loss disrupts the capacity of progenitor and glial populations to adapt to early-life adversity.

The lack of additive or synergistic ELS effects on neurogenic or glial measures contrasts with prior reports in other neurodevelopmental disorder models, in which early adversity amplifies cellular and behavioral phenotypes, such as phenotypes observed in MECP2- and SHANK3- genetic models of autism, as well as models of binge alcohol drinking, changes in maternal care, and maternal immune activation that can lead to neurodevelopmental changes [81,82,83,84,85,86]. In this context, our findings hint at a distinct form of G × E interaction in FXS, characterized not by exacerbation but by attenuation or blunting of stress effects. One explanation is that *Fmr1* KO mice already exhibit a ceiling effect in baseline abDGCs and glial activity, limiting further modulation by ELS. Alternatively, as explained above, blunted HPA axis reactivity and atypical stress responsivity reported in FXS [26,28,29] may render these mice less capable of mounting the typical glucocorticoid-dependent adaptations to early-life challenges. This could create a “stress-buffered” phenotype in which cellular processes are insulated from environmental perturbation, not because of resilience per se, but because the neurogenic niche is less flexible or less responsive to dynamic inputs. By situating *Fmr1* loss within this spectrum of G × E effects, our results suggest that reduced environmental sensitivity may be an underappreciated consequence of genetic risk in neurodevelopmental disorders. Such rigidity may underlie broader patterns of reduced experience-dependent plasticity observed in FXS models [87,88]. Future studies incorporating direct measures of glucocorticoid signaling, stress hormone levels, cytokine signaling, and glial activation states will be essential to test this hypothesis.

It is important to note that the factors driving the interaction between genetic susceptibility to FXS and ELS in the current study were not uniform but instead varied across cell populations—whether proliferating cells, apoptotic cells, immature neurons, or glia—indicating that ELS effects in FXS individuals are nuanced, intricate, and cell-type specific. For example, genotype and ELS were both significant contributors to the changes in Ki67+ number (Figure 1), whereas genetic vulnerability was the primary driver of alterations in DCX+ neurons, with ELS having no detectable contribution at any age (Figure 3). With respect to glial populations, genotype—but not ELS—accounted for increased Iba1+ microglial density (Figure 4), while both factors contributed to changes in GFAP+ astrocyte density (Figure 5). These cell-type-specific effects likely reflect differential sensitivity of proliferative, neurogenic, and glial pathways to FMRP loss and early-life adversity, and may underlie distinct components of the cognitive, emotional, and behavioral phenotypes associated with FXS. These findings underscore the need for continued investigation into how environmental influences interact with *Fmr1* loss to shape neurogenesis and gliogenesis throughout development and into adulthood.

While these data provide insight into how FMRP loss modulates cellular adaptation to early-life stress, several limitations warrant consideration. Our study provides a comprehensive cellular analysis across multiple developmental ages, but we did not assess the functional outcomes of altered neurogenesis and glial density, such as dendritic complexity, synaptic integration, or behavioral effects. Addressing this gap represents a high-priority future direction, particularly through experiments combining BrdU-based lineage tracing with activity-dependent markers (e.g., c-Fos) to link abDGC cell fate to circuit integration and behavior. Lineage tracing of abDGCs would also clarify whether the increased abDGC proliferation in KO mice results in aberrant glial differentiation or in nonproductive cycling through the cell cycle. Lastly, the ELS paradigm may not capture the full range of environmental stressors relevant to FXS in humans. Future work should integrate longitudinal assessments of physiological stress responses and single-cell transcriptomic profiling to delineate how FMRP loss and environmental stress converge on shared molecular pathways.

In summary, the loss of FMRP disrupts the coordination of neurogenic and glial processes in abDGCs, altering how these cells respond to ELS. The resulting cellular imbalance may underlie aspects of the cognitive and emotional dysregulation characteristic of FXS. By identifying the developmental and cell-type-specific effects of *Fmr1* loss, this work highlights the importance of considering both genetic vulnerability and environmental context in understanding the pathophysiology of neurodevelopmental disorders.

## 4. Materials and Methods

### 4.1. Mice

Animals used in this study included adult male *Fmr1* knockout and wild-type mice on a C57BL/6J background strain (Jackson Laboratory Stock No. 003025) [89]. Female heterozygous (+/−) mice were bred with male wildtype (+/+) mice to produce male wildtype (+/+) mice (WT) and male knockout (−/−) mice (KO). Since *Fmr1* is an X-linked gene, male mice lacking the gene on their X chromosome are considered a full knockout. All mice were bred and housed as cage mates at Baylor University under standard laboratory conditions with an ambient temperature of 22 °C and a 12 h light/dark diurnal cycle. Mice were provided with water and food ad libitum. All procedures were conducted in compliance with the Baylor University Institutional Animal Care and Use Committee and the National Institutes of Health Guidelines for the Care and Use of Laboratory Animals [90,91].

Early-life stress (ELS) was induced using a limited bedding and nesting (LBN) stress paradigm, as previously described by others [43,69,70,92], with some modifications. Sires were removed from the home cage once pregnancy was suspected, and pregnant dams received standard health monitoring until birth. At P2, litters were randomly assigned to the control or ELS condition. Dams and pups assigned to the ELS condition were moved into a standard cage with wire mesh covering the typical amount of wood-shaving bedding, allowing feces and urine to drop through without allowing nesting use. The dam was given two-thirds of the standard nestlet (2″ × 1″) for nesting. The dam and pups remained undisturbed in the stressful environment until P9. Control mice were kept in standard rearing conditions following birth. Therefore, mice were exposed to the LBN paradigm during a developmental window (P2-P9), corresponding to the third trimester of human gestation [71,72,73]. At P9, the pups and dams in the ELS environment were returned to the home cage under standard rearing conditions. Throughout all procedures, manipulation was kept to a minimum to avoid handling effects, and animals were left undisturbed until P9. All animals were supplied with food and water ad libitum.

### 4.2. Tissue Collection and Immunohistochemistry (IHC)

Mice were anesthetized using isoflurane before intracardial perfusion with 4 °C 0.1 M phosphate-buffered saline (PBS) containing 2 IU/mL heparin for exsanguination, followed by 4% paraformaldehyde for fixation. Brains were extracted and immersed in 4% paraformaldehyde in 0.1 M PBS at 4 °C for 24 h, followed by cryoprotection in 0.1 M PBS with 30% sucrose and 0.01% sodium azide. The brain of each mouse was sectioned in the coronal plane extending from anterior to the lateral ventricles to the cerebellum (1.70 to −4.20 mm from Bregma) and was sectioned at 30 μm using a cryostat (Thermo Scientific HM 525NX Cryostat) in a 1:10 series to permit stereological quantification. Brain sections were stored in PBS with 0.01% NaN3 at 4 °C until IHC.

Slide-mounted IHC for immunopositive cells in the dentate gyrus (Ki67+, doublecortin [DCX]+, cleaved caspase-3 [CC3]+, ionized calcium-binding adapter molecule 1 [Iba1]+, and Glial Fibrillary Acidic Protein [GFAP]+ cells) was performed as previously described [44,93]. For each IHC procedure, one entire series containing the hippocampus (−0.90 to −4.20 mm from Bregma; every 10th section) was slide-mounted onto Superfrost-Plus charged slides (ThermoFisher Scientific, 12-550-16, Pittsburgh, PA, USA) and allowed to dry for 2 h. We performed antigen retrieval on slide-mounted sections (0.01 M citric acid, pH 6.0, 90–95 °C, 15 min) followed by washing in room temperature 1× PBS. For CC3 IHC, two additional antigen retrieval steps were performed: permeabilization (0.1% Trypsin in 0.1 M Tris and 0.1% CaCl_2_, 10 min) and denaturation (2N HCl in 1× PBS, 30 min). Endogenous peroxidase activity was inhibited via incubation with 0.3% hydrogen peroxide (H_2_O_2_) for 30 min. Non-specific binding was blocked with 3% serum (goat) and 0.3% Triton-X 100 in 1× PBS for 60 min. The sections were then incubated overnight with the appropriate primary antibody in 3% serum and 0.3% Tween-20. The following primary antibodies were used: rabbit-anti-Ki67 (1:500; ThermoFisher Scientific, 514520, Pittsburgh, PA, USA), guinea pig-anti-DCX (1:500; Millipore, AB2253, Billerica, MA, USA), rabbit-anti-Iba1 (1:1000; Wako Chemicals, 019–19741, Richmond, VA, USA), rabbit-anti-GFAP (1:1000; Millipore, AB5804, Billerica, MA, USA), and rabbit-anti-cleaved caspase-3 (1:500; Cell Signaling, 9661S, Danvers, MA, USA). Primary antibody incubation was followed by 1× PBS rinses and incubation with biotinylated secondary antibodies (goat-anti-rabbit-IgG, 111-065-003; goat-a-guinea pig-IgG, 106-065-003; all 1:200 and from Jackson ImmunoResearch, West Grove, PA, USA) for 2 h. After additional 1× PBS rinses, slides were incubated with an avidin-biotin complex for 90 min (Elite ABC-HRP Kit, PK-6100, Vector Laboratories, Burlingame, CA, USA). After another set of rinses in 1× PBS, immunoreactive cells were visualized by incubation with 3,3′-diaminobenzidine metal-concentrate (ThermoFisher Scientific, PI34065, Pittsburgh, PA, USA) for ~10 min. Slides were counterstained with Nuclear Fast Red (Vector Laboratories, H-3403-500, Pittsburgh, PA, USA). We then performed a series of increasing ethanol concentrations to dehydrate the sections and coverslipped using DPX mountant (ThermoFisher Scientific, 50-980-370, Pittsburgh, PA, USA).

### 4.3. Ki67+, CC3+, and DCX+ Stereology

Unbiased stereology was used to enumerate Ki67-, CC3-, and DCX-immunopositive cells. Due to their rarity [93,94], Ki67+ and CC3+ cells were quantified in every 10th section throughout the entire hippocampus (Bregma levels −0.90 to −4.20). Cells were visualized with a Nikon Eclipse 80i microscope at 400× total magnification (40× objective; NA 0.75) with continuous adjustment through the depth of the section. Characteristics considered when determining Ki67+ or CC3+ cells were size, color, shape, transparency, location, and focal plane [93]. As described in Latchney et al. [93], Ki67+ and CC3+ cells were counted in four areas of the dentate gyrus: the SGZ (30 μm into the hilus and the inner half of the GCL), frequently considered to be the “neurogenic niche” of the dentate gyrus [95,96,97]; the outer GCL (oGCL), to which a small number of adult-generated cells migrate [98]; the hilus, through which dentate gyrus granule cells extend their processes toward CA3; and the molecular layer, where the dendrites of dentate gyrus granule cells are located.

Total cell counts were multiplied by a section sampling fraction of 10 to attain an estimate of the total cell number. Because initial cell counts for immunopositive cells were low according to dissector/fractionator standards, the area and height sampling fractions were both set to 1, as is used for rare cell populations [93,94]. All cell quantification was performed blindly.

DCX+ cells were quantified via unbiased stereology in the SGZ and the entire GCL using the optical fractionator procedure in Stereo Investigator (MicroBrightField; [93]). DCX+ cells in the hilus and molecular layer appeared faint and ill-defined and were not enumerated. Every 10th coronal section throughout the hippocampus was analyzed on a Zeiss AxioImager M2 microscope. The SGZ and GCL were outlined for each section at 100× total magnification (10× objective, NA 0.30). An unbiased counting frame was overlaid on the region of interest to enumerate immunopositive cells. Quantification was performed at 400× (40× objective, NA 0.75) by focusing throughout the depth of the section. At least 200 cells per mouse were counted and the average number of counting fields per mouse was close to 300 in an average of 10 sections per mouse [93]. DCX+ cells were counted if they satisfied three criteria: the entire border of the cell body was intact, there was a dendritic process emerging from the cell body, and the cell body was darker than the surrounding background [93]. To minimize shrinkage effects on the tissue, the average measured mounting thickness after processing was maintained at ~20 µm, and an optical dissector height of 12 mm was used. The area-sampling fraction was 1/10, and every 10th section was used to obtain a total estimate of the number of DCX+ cells within the SGZ and GCL. The Gunderson coefficient of variance for each mouse quantified was always <10% [93]. Data are reported as the total number of DCX+ cells per brain. All quantification was performed blindly.

### 4.4. Iba1+ and GFAP+ Cell Density Quantification

Iba1+ and GFAP+ cells were quantified in four anatomically matched sections throughout the entire hippocampus corresponding to −1.50, −2.0, −2.50, and −3.0 mm from Bregma [44]. Cells were visualized with a Nikon Eclipse 80i microscope at 400× total magnification (40× objective; NA 0.75) with continuous adjustment through the depth of the section. Criteria for inclusion as an Iba1+ microglia or a GFAP+ astrocyte were a consistently labeled soma easily distinguishable from the counterstain and the presence of an entire cross-sectional view through the soma in the photomicrograph focal plane. Images were taken at 100× magnification (10× objective; NA 0.30) to capture the entire dentate gyrus region, and immunopositive cells were quantified across the entire dentate gyrus, which included the SGZ, GCL, Hilus, and the Molecular Layer. The area was measured in pixels, converted to square millimeters (mm^2^), and cell counts were divided by their respective areas to obtain cell density. Density values were averaged such that each dentate gyrus was represented by one density value. All cell quantification was performed blindly.

### 4.5. Iba1+ and GFAP+ Spacing Analysis

The cellular coordinates obtained from the density calculations (Section 4.4) were analyzed using a custom algorithm implemented in MATLAB [MathWorks version R2025b [44]] to determine the average straight-line distance between each cell and its nearest neighbor based on the Pythagorean theorem (Nearest Neighbor distance; an example is shown in Figure 4E). An average Nearest Neighbor distance was calculated for all cells in an image for each animal and then averaged by group. A higher Nearest Neighbor value indicates that cells are spread further apart, and a lower value indicates that cells are closer together. The Nearest Neighbor distance was then corrected for density by calculating a spatial index value (or “clustering index”). This was calculated as (average nearest neighbor distance)^2^ × microglial density.

### 4.6. Data Presentation and Analysis and Image Presentation

Experimenters were blinded, and the code was broken after data analysis. Data are displayed as individual data points with the mean and standard error of the mean. All data were assessed for normality with the Shapiro–Wilk test. The Grubbs Outlier test (GraphPad Prism, version 10) was used to check for and exclude statistical outliers. Statistical analyses were performed using a 2-way ANOVA (Genotype [WT, KO] × Condition [Control, ELS]) and graphs were generated in GraphPad Prism (version 10). Tukey’s multiple comparisons test was used to analyze significant Genotype × Condition interactions. *p* ≤ 0.05 denoted statistical significance. Images were captured using a Zeiss Axiocam 305 camera with Zeiss Zen Blue software (version 3.7) and then imported into FIJI for labeling.

## 5. Conclusions

Our results demonstrate that loss of FMRP produces an imbalance in hippocampal neurogenesis and gliogenesis that unfolds across development and reshapes cellular responsiveness to early-life adversity. *Fmr1* KO mice exhibited increases in abDGC proliferation, accompanied by reduced immature neuron numbers and elevated microglial and astrocytic densities. In addition, rather than amplifying canonical stress effects, early-life stress induced cell-type-specific changes in KO mice, suggesting that FMRP deficiency alters the set point and plasticity of the neurogenic niche. This “stress-buffered,” but likely inflexible phenotype, may reflect dysregulated translational control of pathways involved in glucocorticoid signaling, inflammation, and cell lineage decisions. Importantly, the effects of genotype and environment were not uniform across proliferative, neuronal, and glial populations, underscoring the need to consider developmental timing and cell-type specificity when modeling gene × environment interactions in FXS. By delineating how FMRP loss modifies baseline neurogenesis and gliogenesis, as well as stress responsivity, this study advances our understanding of mechanisms that may contribute to cognitive and affective symptoms in FXS and highlights potential cellular targets for therapeutic intervention.

## Data Availability

The original contributions presented in this study are included in the article. Further inquiries can be directed to the corresponding author.

## Supplementary Materials

The following supporting information can be downloaded at: https://www.mdpi.com/article/10.3390/ijms27104356/s1.

## Abbreviations

The following abbreviations are used in this manuscript:

abDGC	adult-born dentate granule cells
BrdU	5-bromo-2′-deoxyuridine
CC3	Cleaved caspase-3
CIdU	5′-chloro-2′-deoxyuridine
DCX	Doublecortin
GCL	Granule cell layer
ELS	Early-life stress
*Fmr1*	Fragile X messenger ribonucleoprotein 1 gene
FMRP	Fragile X messenger ribonucleoprotein
FXS	Fragile X syndrome
GFAP	Glial fibrillary acidic protein
HPA	hypothalamic–pituitary–adrenal
Iba1	Ionized calcium-binding adapter molecule 1
IHC	Immunohistochemistry
KO	Knockout
LBN	Limited bedding and nesting
NeuN	Neuronal nuclei
P	Postnatal
PBS	Phosphate-buffered saline
SGZ	Subgranular zone
WT	Wild-type
