# Supplementary figures and images for: Fmr1 Deletion and Early-Life Stress Interact to Increase Cell Proliferation and Glial Populations at the Expense of Immature Neurons in the Adult Dentate Gyrus
Source: Int J Mol Sci. 2026 May 14;27(10):4356. doi: 10.3390/ijms27104356 (PMC13207772; doi:10.3390/ijms27104356)

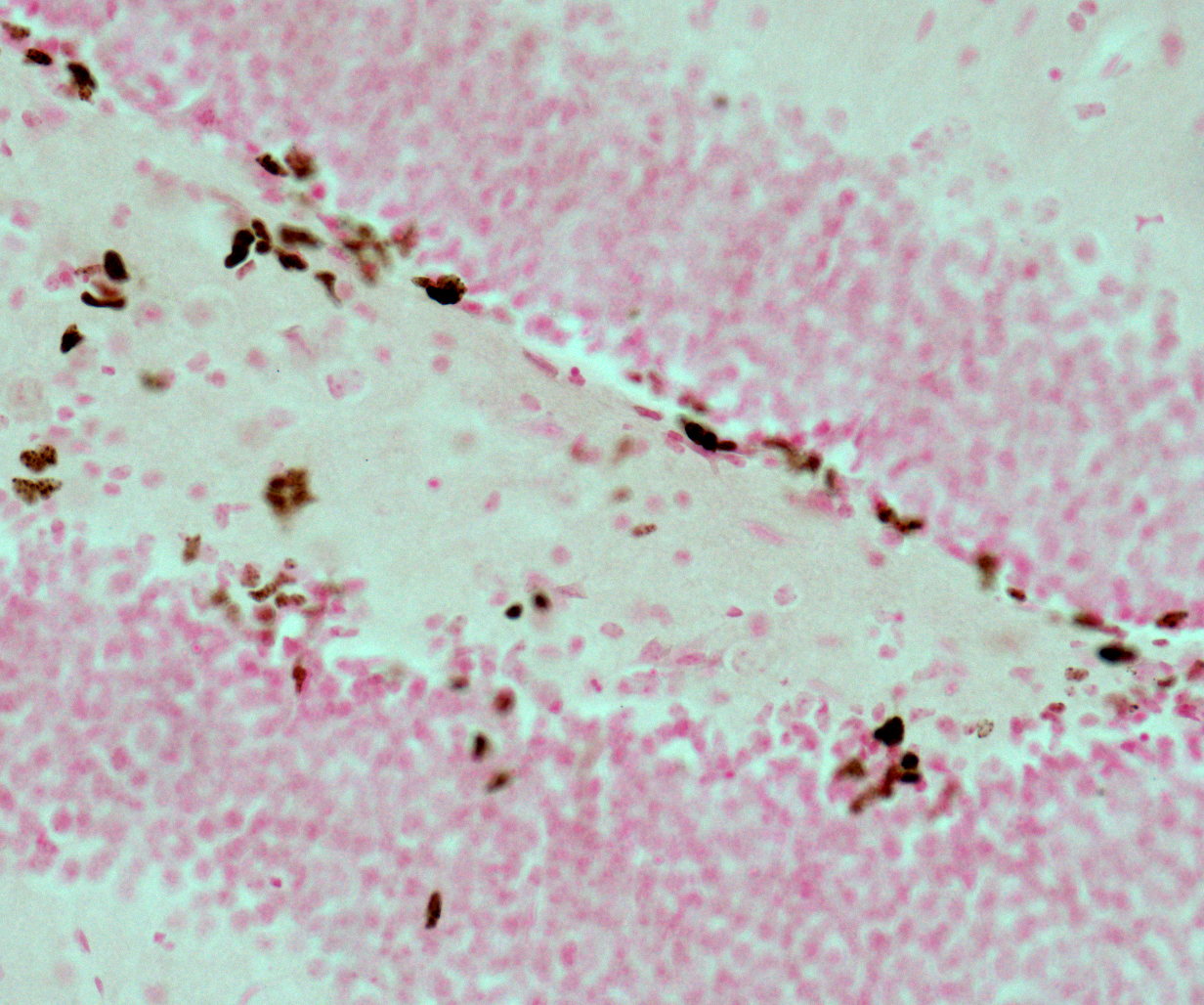

Supplement: Supplementary file 1 [file ijms-27-04356-s001.zip › Supplementary Figures/Supplementary Figure S1. Ki67/Ki67 KO Control/20x_Ki67.tif]

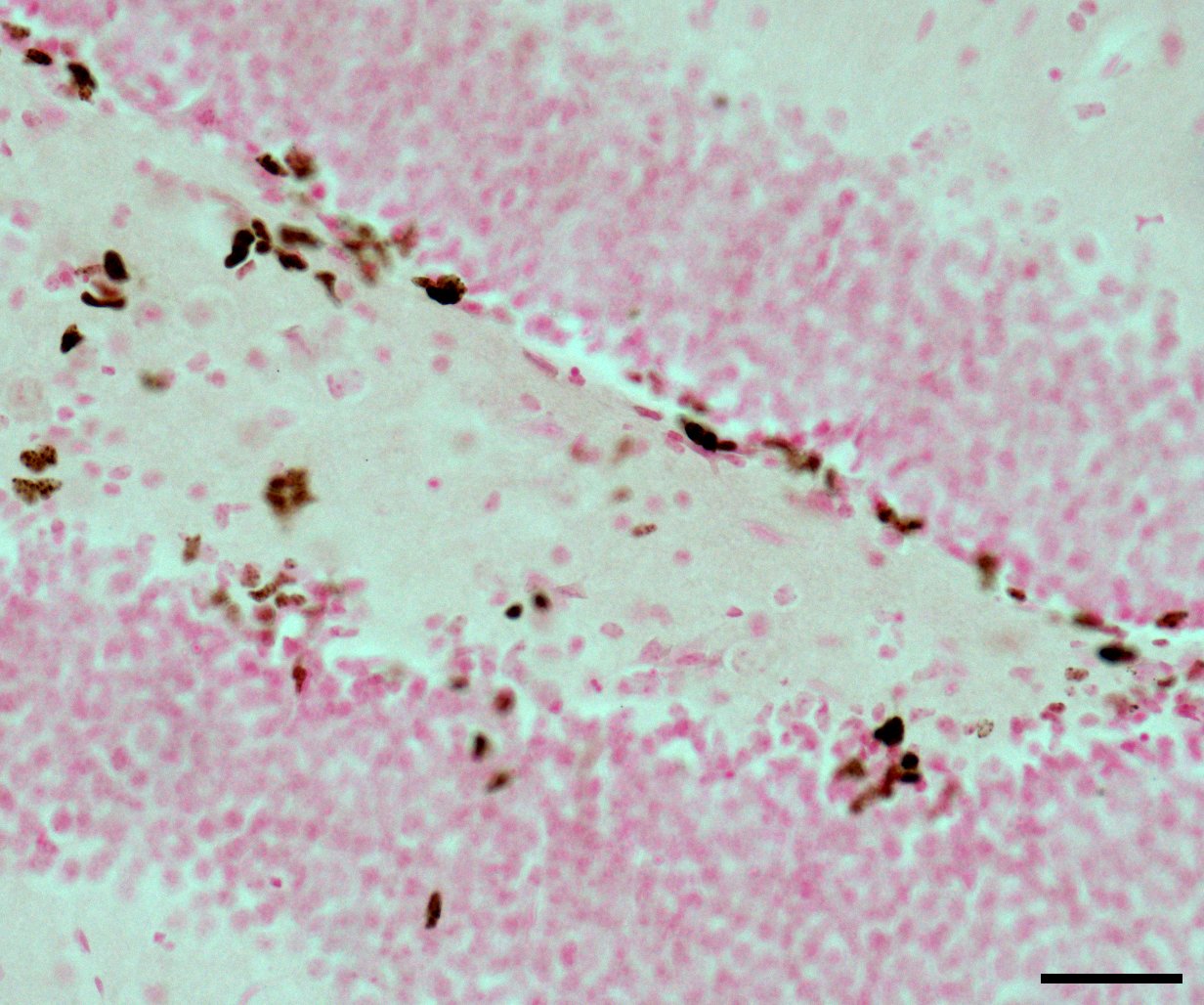

Supplement: Supplementary file 1 [file ijms-27-04356-s001.zip › Supplementary Figures/Supplementary Figure S1. Ki67/Ki67 KO Control/20x_Ki67_scalebar.jpg]

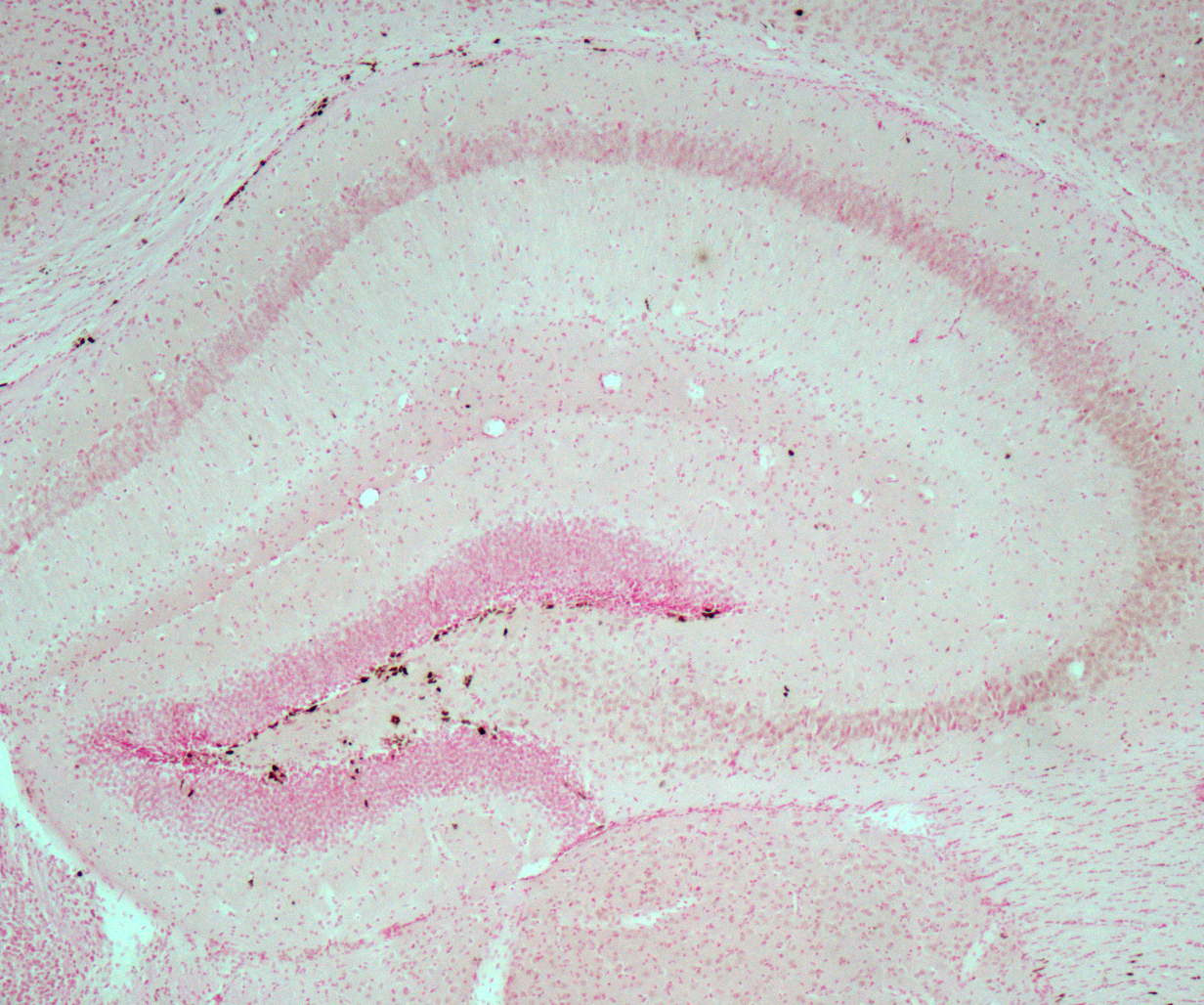

Supplement: Supplementary file 1 [file ijms-27-04356-s001.zip › Supplementary Figures/Supplementary Figure S1. Ki67/Ki67 KO Control/4x_Ki67.tif]

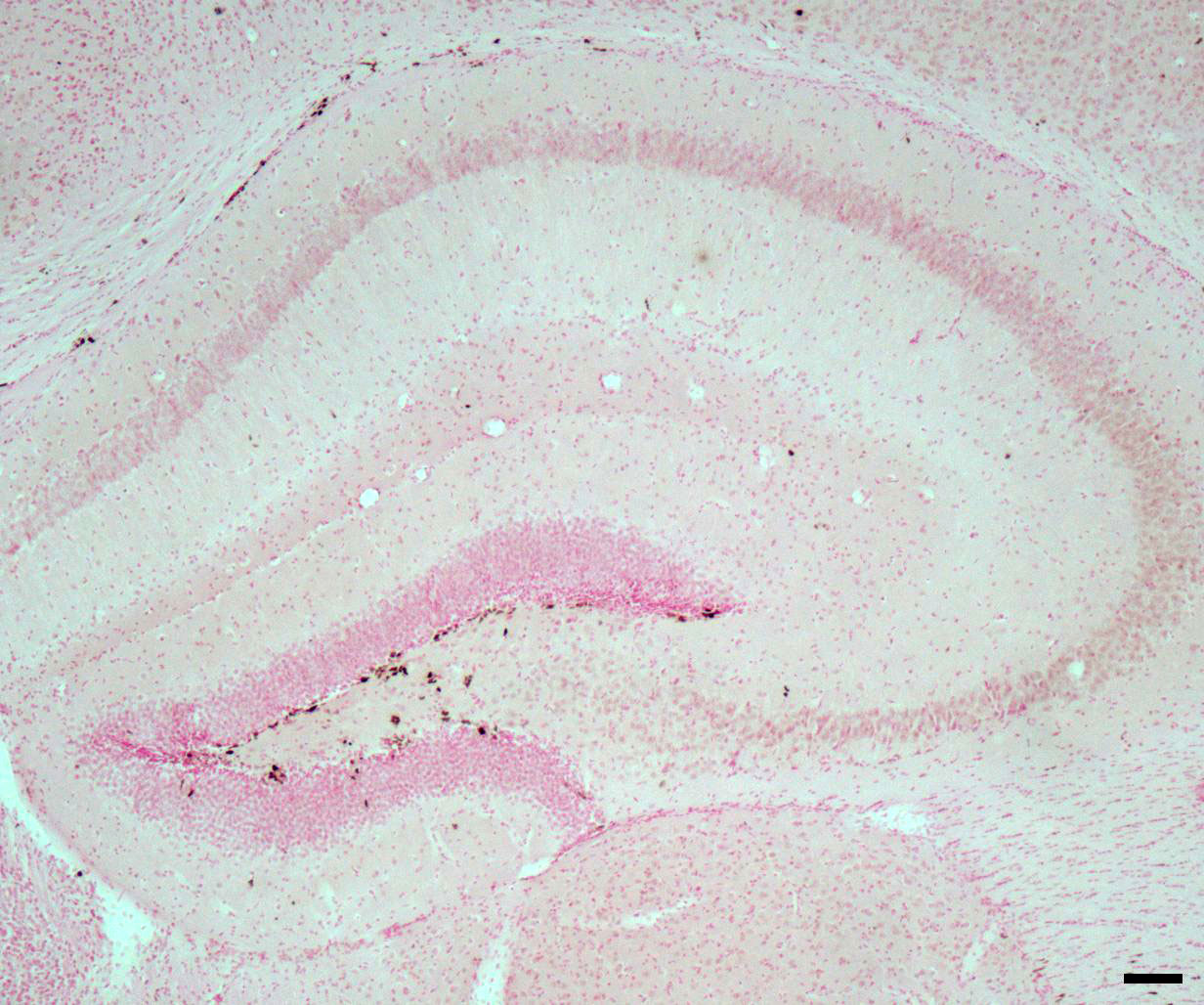

Supplement: Supplementary file 1 [file ijms-27-04356-s001.zip › Supplementary Figures/Supplementary Figure S1. Ki67/Ki67 KO Control/4x_Ki67_scalebar.jpg]

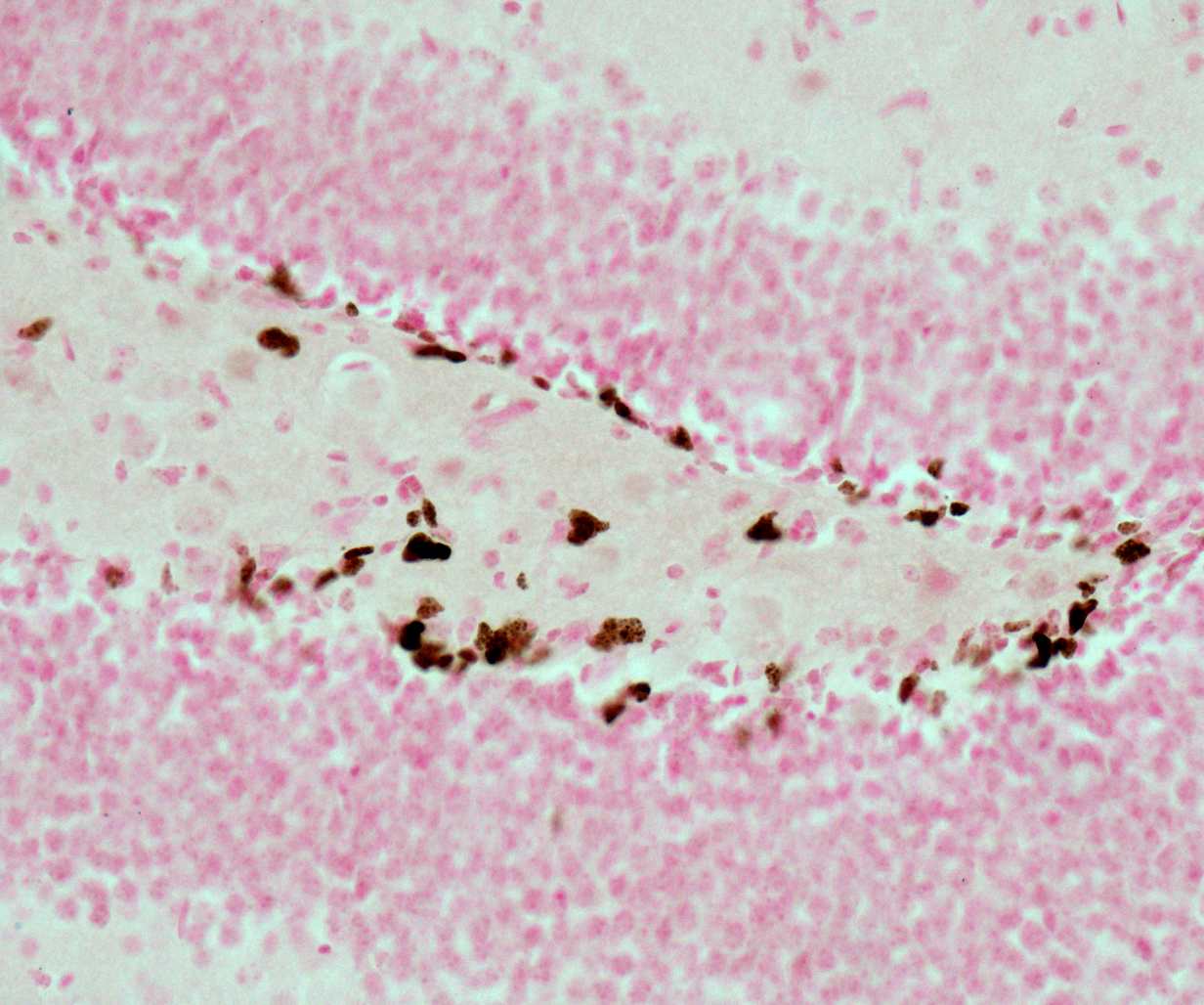

Supplement: Supplementary file 1 [file ijms-27-04356-s001.zip › Supplementary Figures/Supplementary Figure S1. Ki67/Ki67 KO Stress/20x_Ki67.tif]

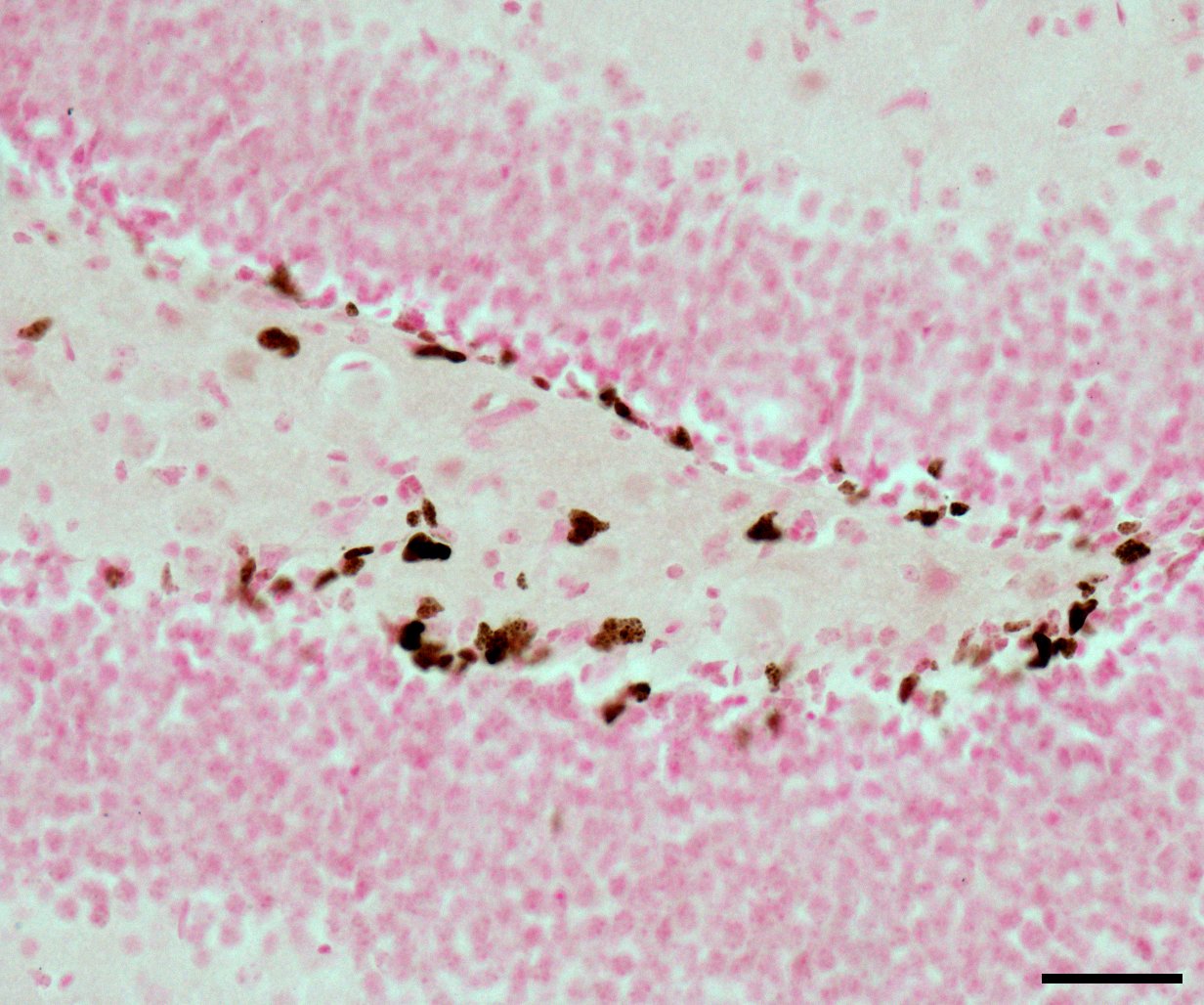

Supplement: Supplementary file 1 [file ijms-27-04356-s001.zip › Supplementary Figures/Supplementary Figure S1. Ki67/Ki67 KO Stress/20x_Ki67_scalebar.jpg]

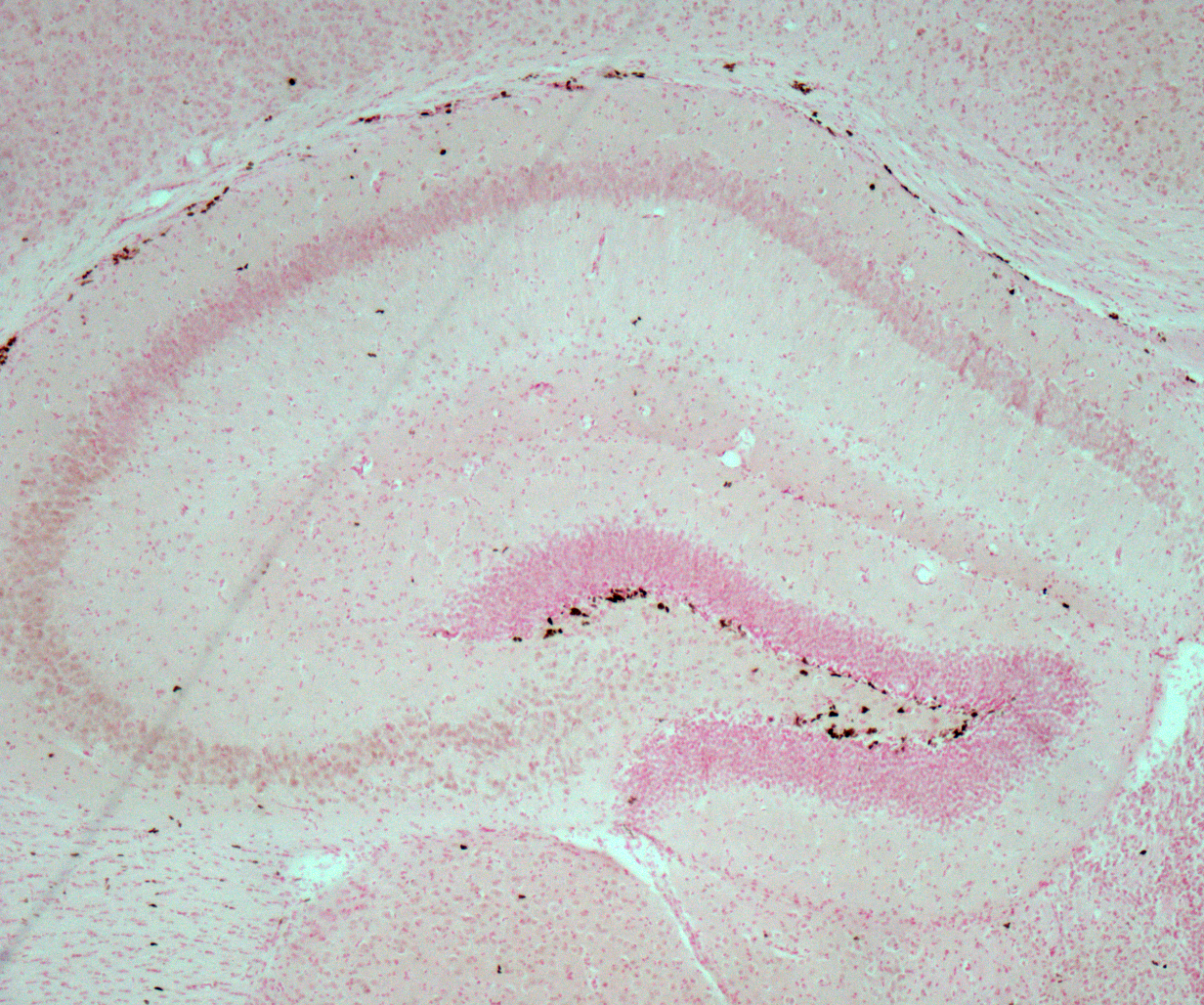

Supplement: Supplementary file 1 [file ijms-27-04356-s001.zip › Supplementary Figures/Supplementary Figure S1. Ki67/Ki67 KO Stress/4x_Ki67.tif]

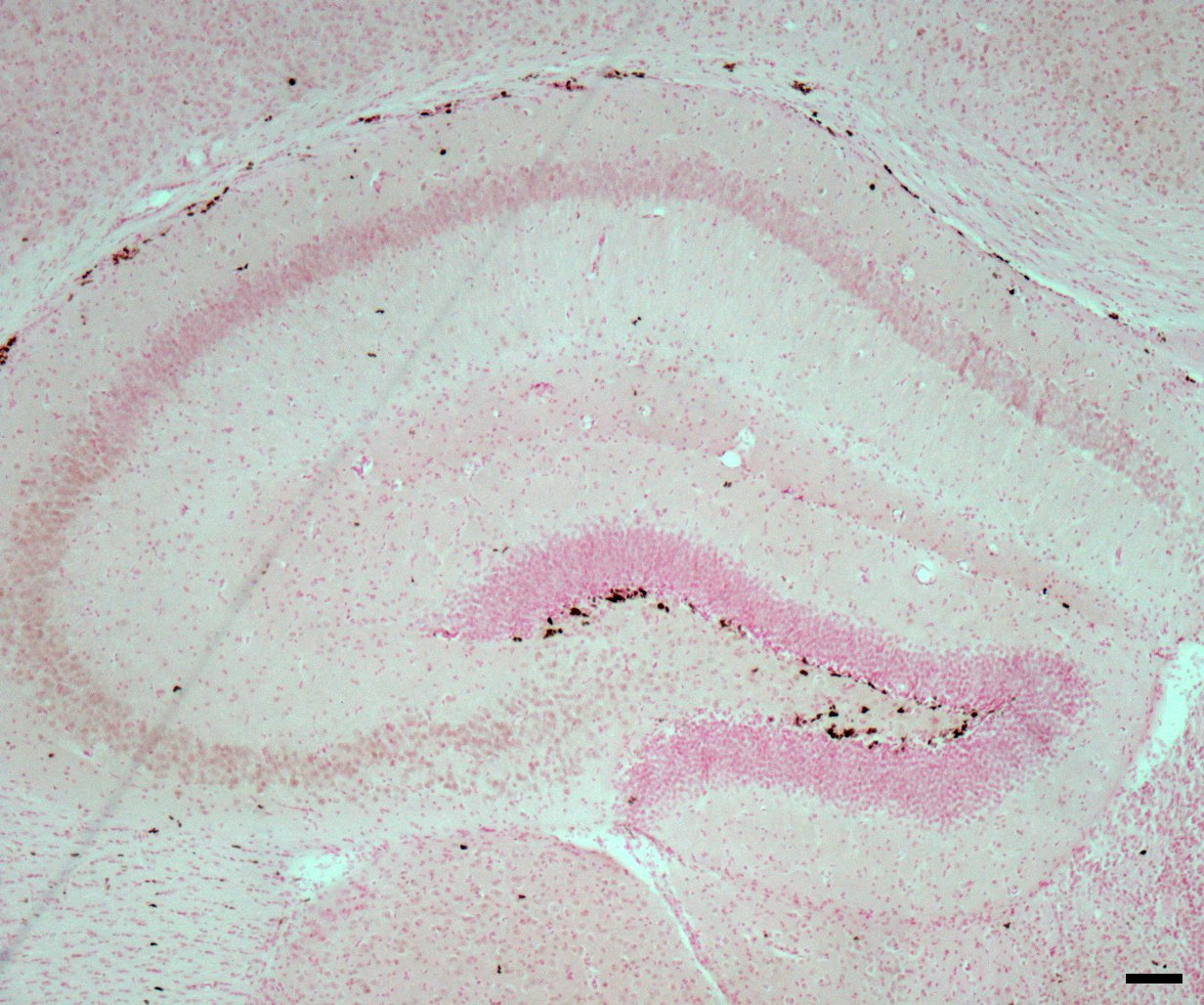

Supplement: Supplementary file 1 [file ijms-27-04356-s001.zip › Supplementary Figures/Supplementary Figure S1. Ki67/Ki67 KO Stress/4x_Ki67_scalebar.jpg]

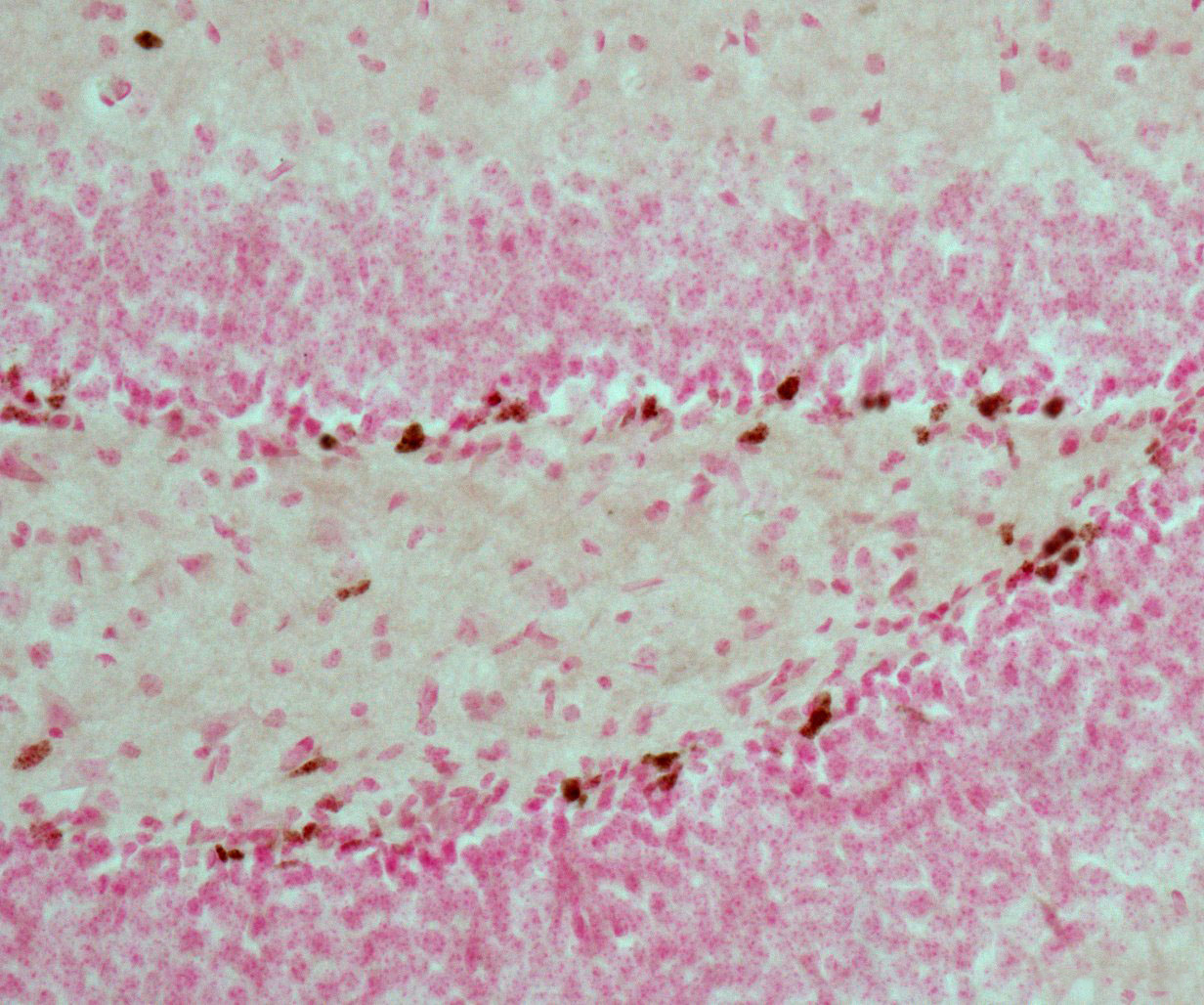

Supplement: Supplementary file 1 [file ijms-27-04356-s001.zip › Supplementary Figures/Supplementary Figure S1. Ki67/Ki67 WT Control/20x_Ki67.tif]

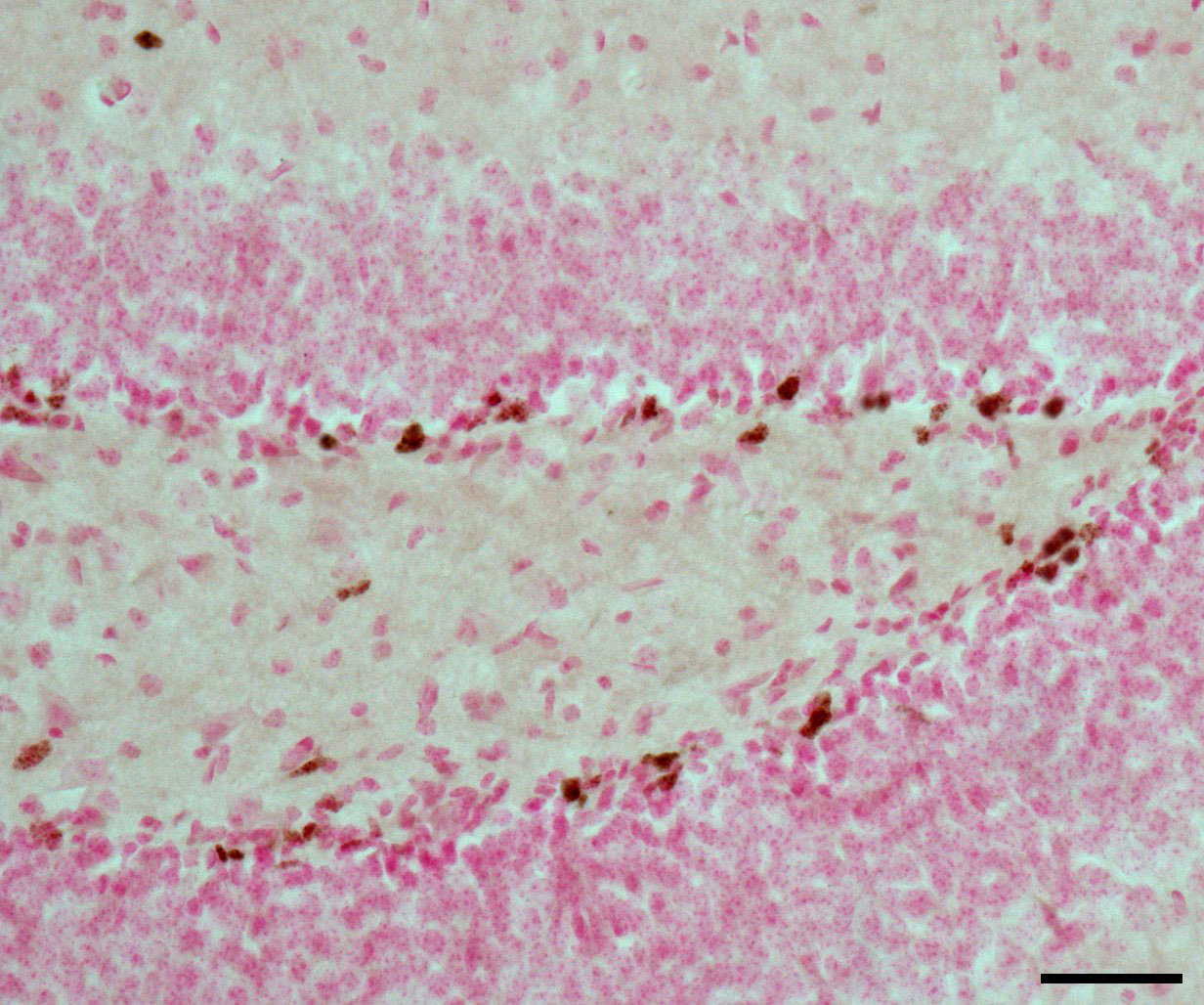

Supplement: Supplementary file 1 [file ijms-27-04356-s001.zip › Supplementary Figures/Supplementary Figure S1. Ki67/Ki67 WT Control/20x_Ki67_scalebar.jpg]

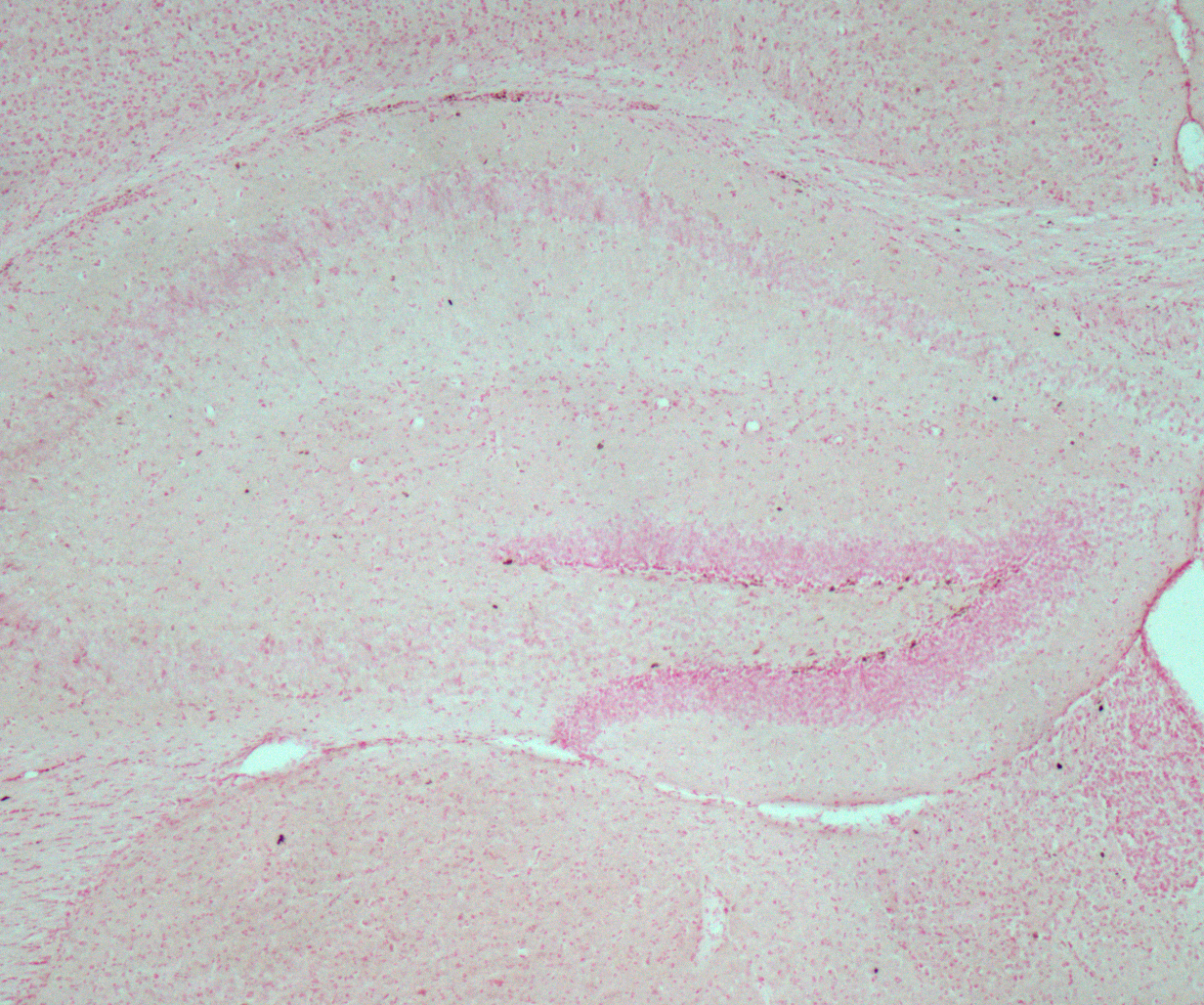

Supplement: Supplementary file 1 [file ijms-27-04356-s001.zip › Supplementary Figures/Supplementary Figure S1. Ki67/Ki67 WT Control/4x_Ki67.tif]

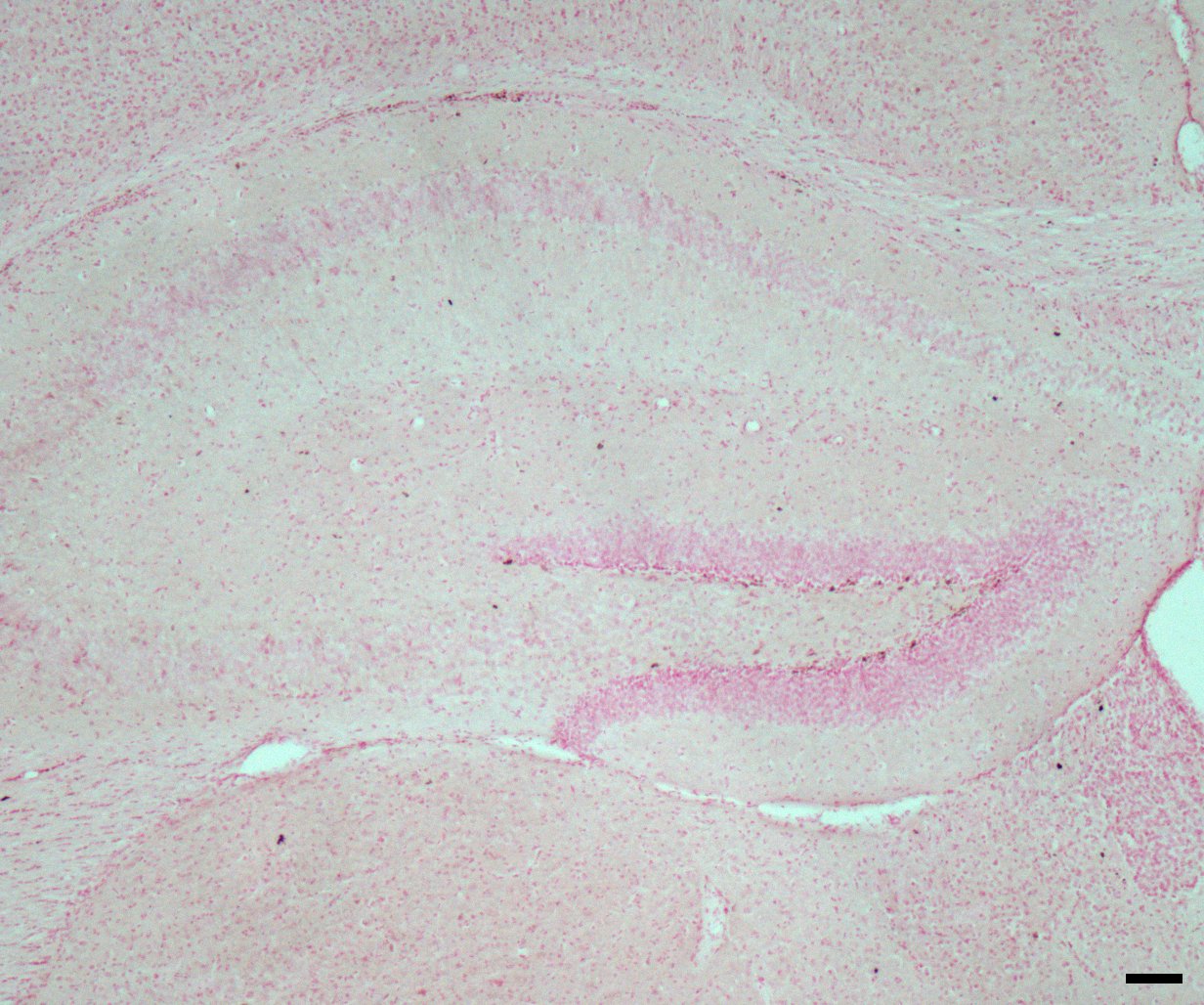

Supplement: Supplementary file 1 [file ijms-27-04356-s001.zip › Supplementary Figures/Supplementary Figure S1. Ki67/Ki67 WT Control/4x_Ki67_scalebar.jpg]

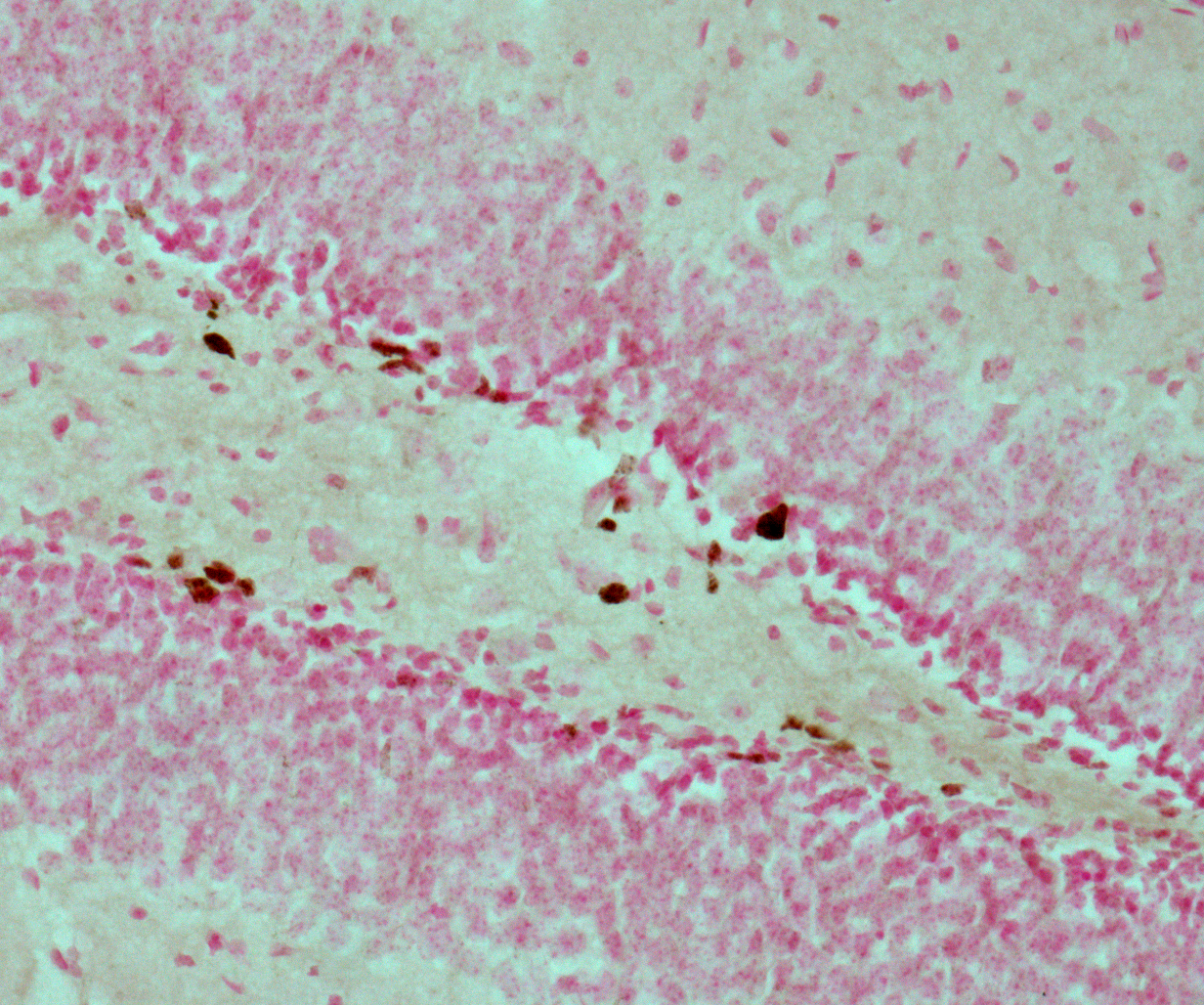

Supplement: Supplementary file 1 [file ijms-27-04356-s001.zip › Supplementary Figures/Supplementary Figure S1. Ki67/Ki67 WT Stress/20x_Ki67.tif]

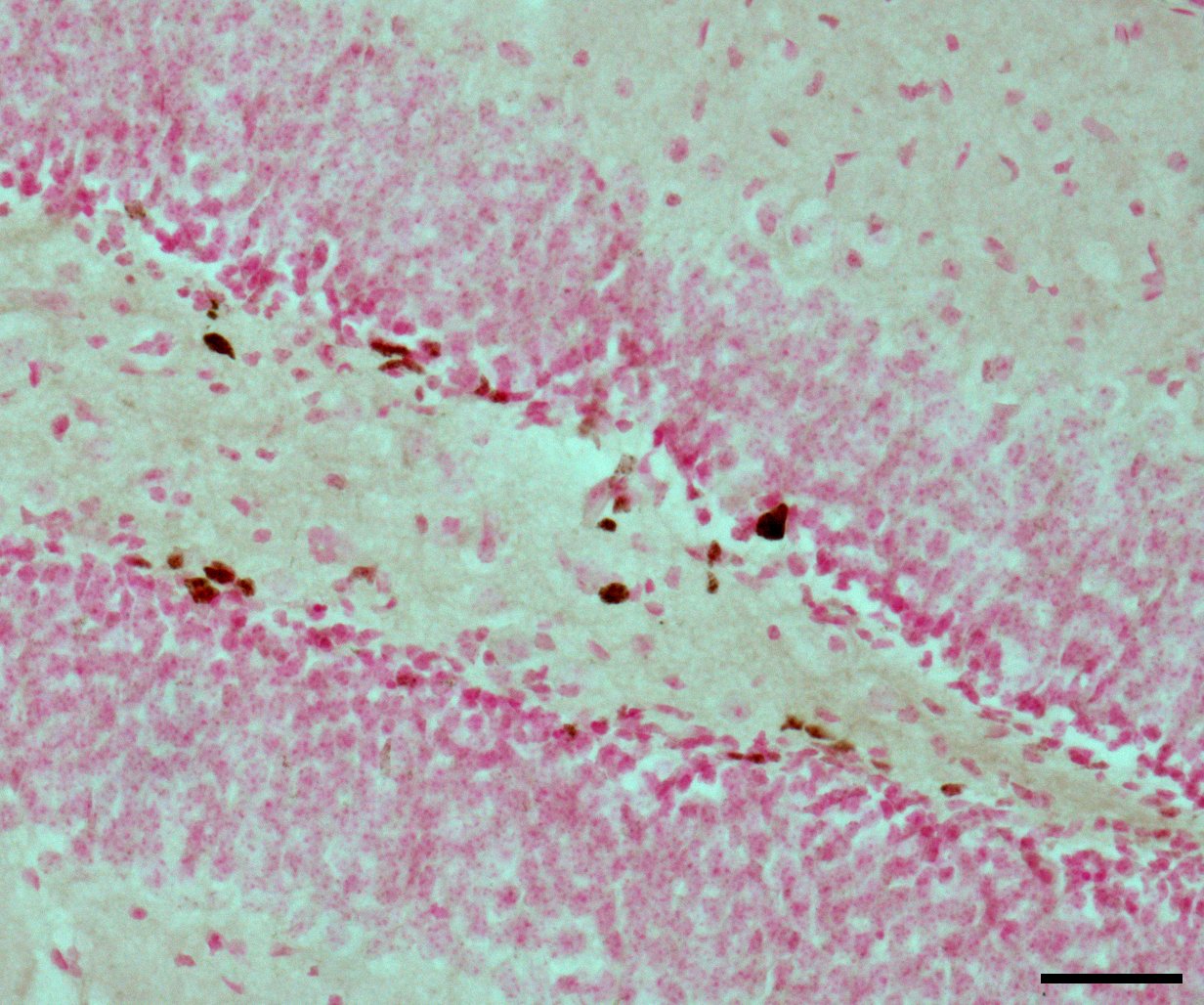

Supplement: Supplementary file 1 [file ijms-27-04356-s001.zip › Supplementary Figures/Supplementary Figure S1. Ki67/Ki67 WT Stress/20x_Ki67_scalebar.jpg]

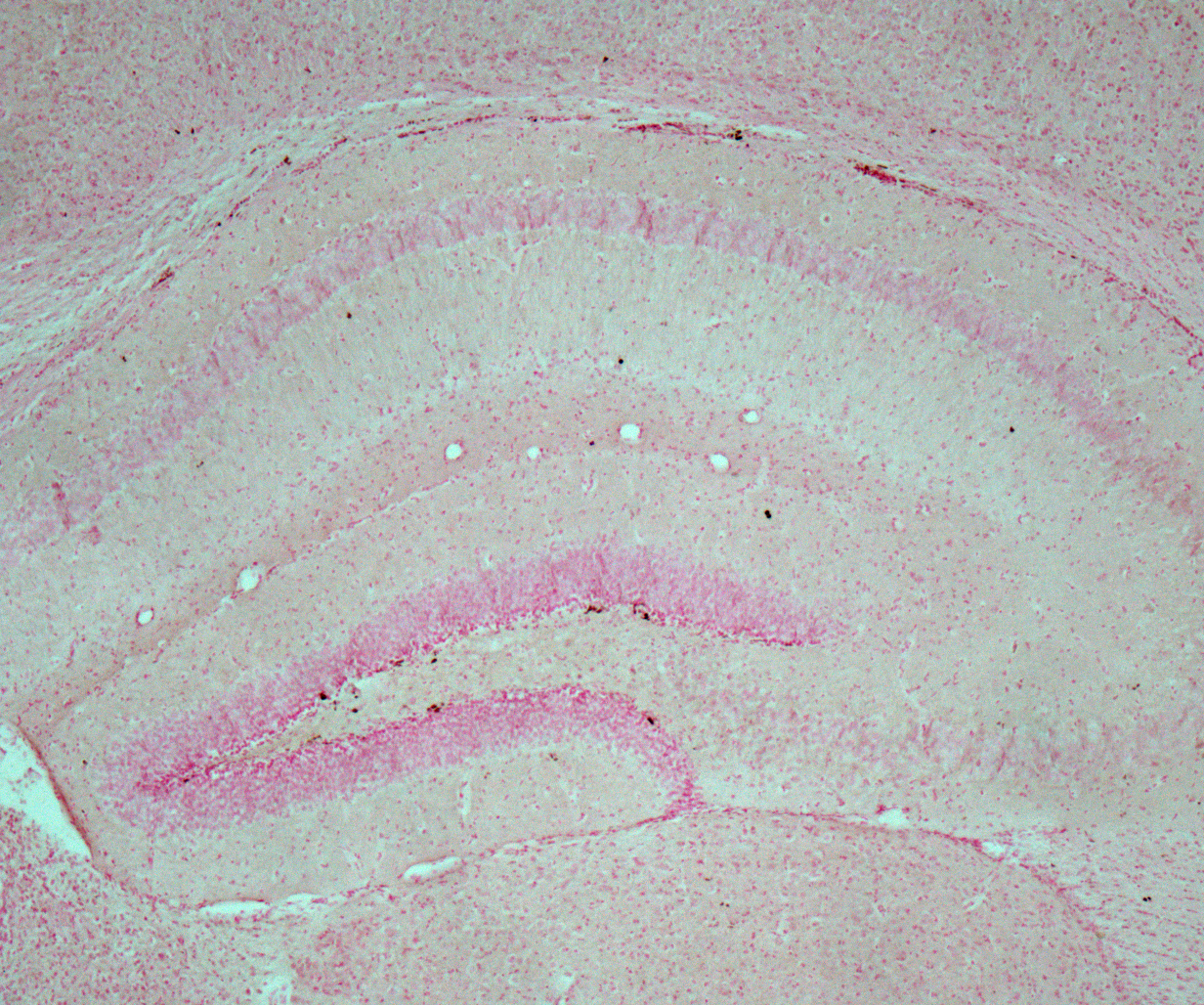

Supplement: Supplementary file 1 [file ijms-27-04356-s001.zip › Supplementary Figures/Supplementary Figure S1. Ki67/Ki67 WT Stress/4x_Ki67.tif]

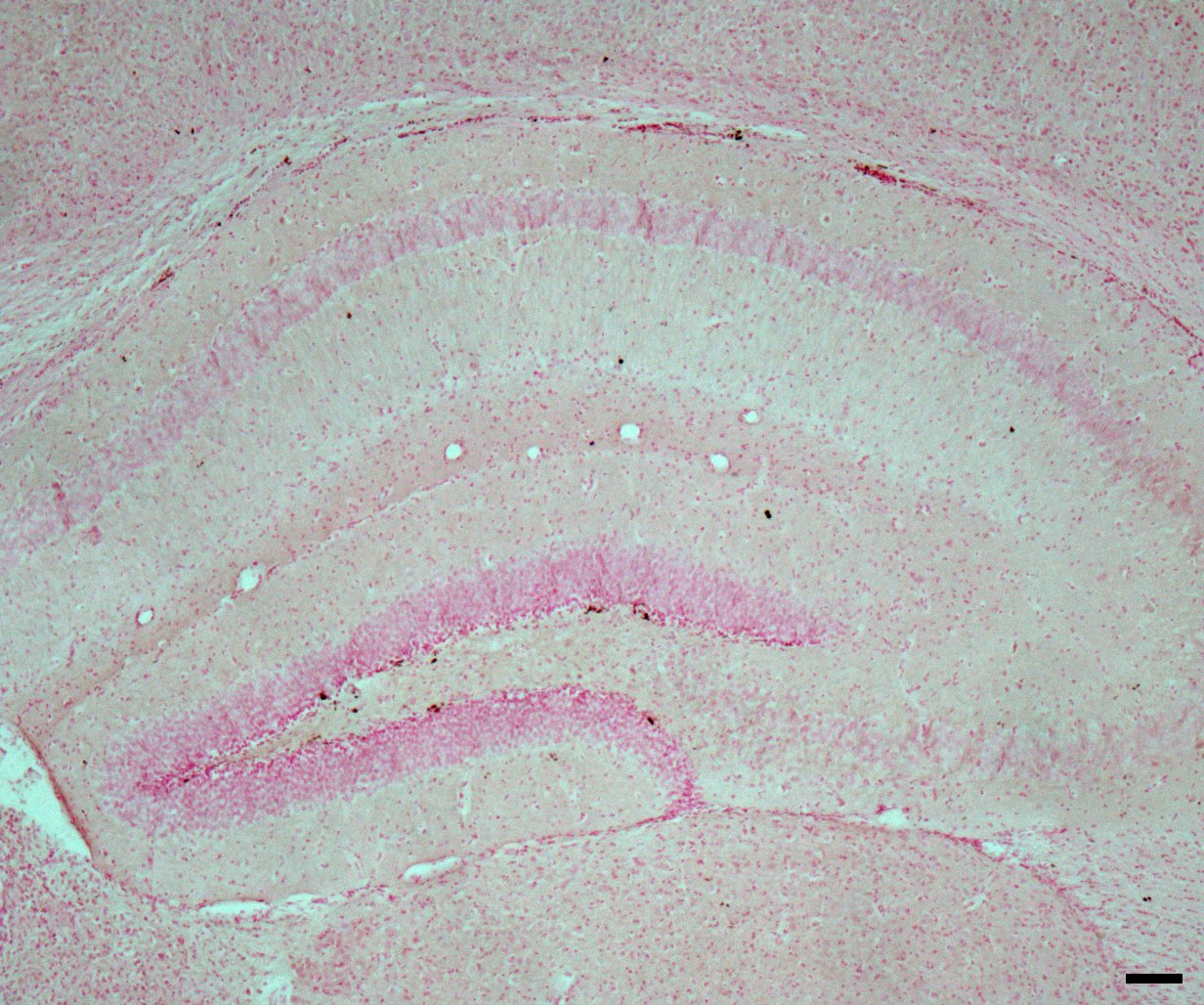

Supplement: Supplementary file 1 [file ijms-27-04356-s001.zip › Supplementary Figures/Supplementary Figure S1. Ki67/Ki67 WT Stress/4x_Ki67_scalebar.jpg]

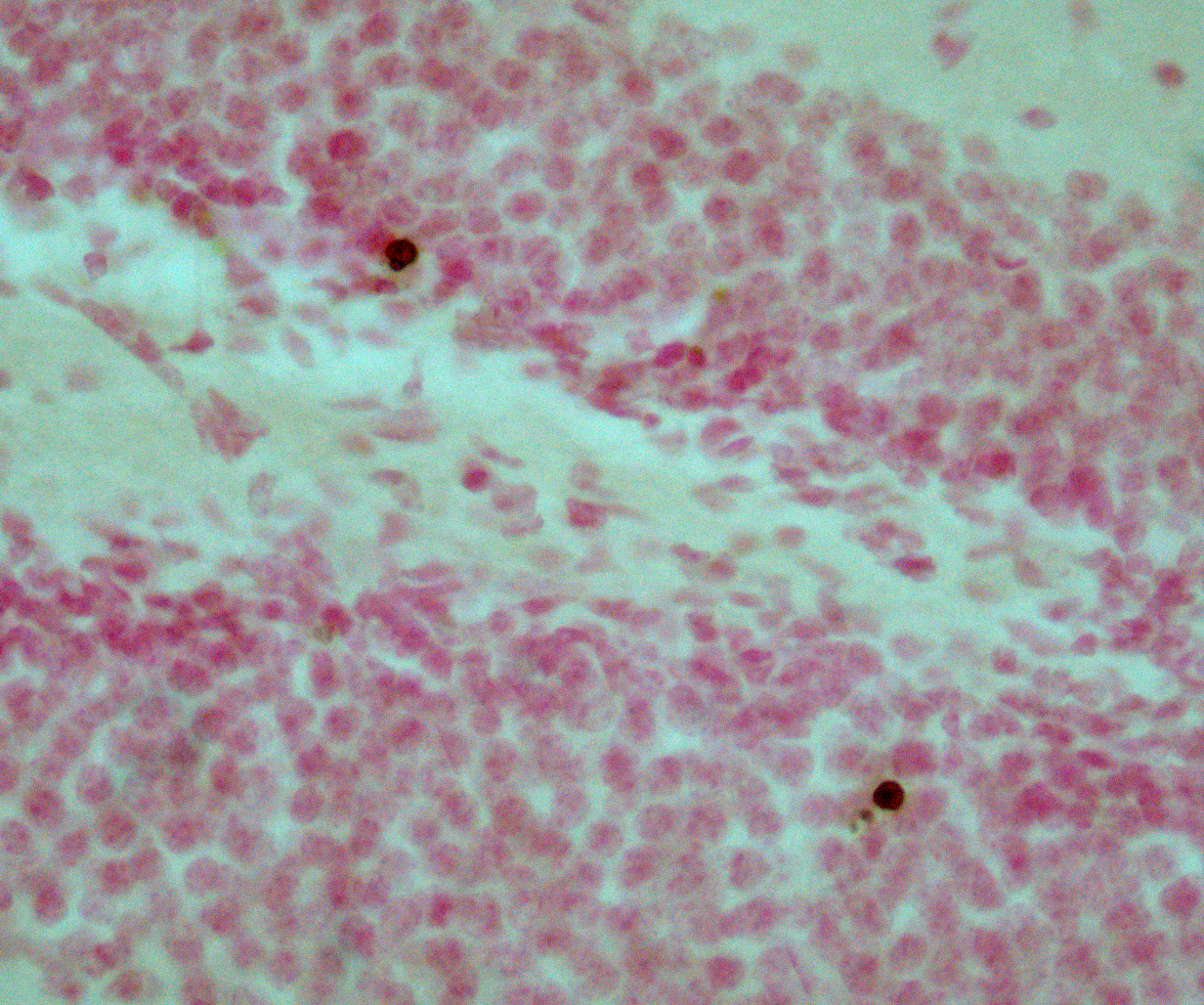

Supplement: Supplementary file 1 [file ijms-27-04356-s001.zip › Supplementary Figures/Supplementary Figure S2. CC3/KO Control/40x_CC3.tif]

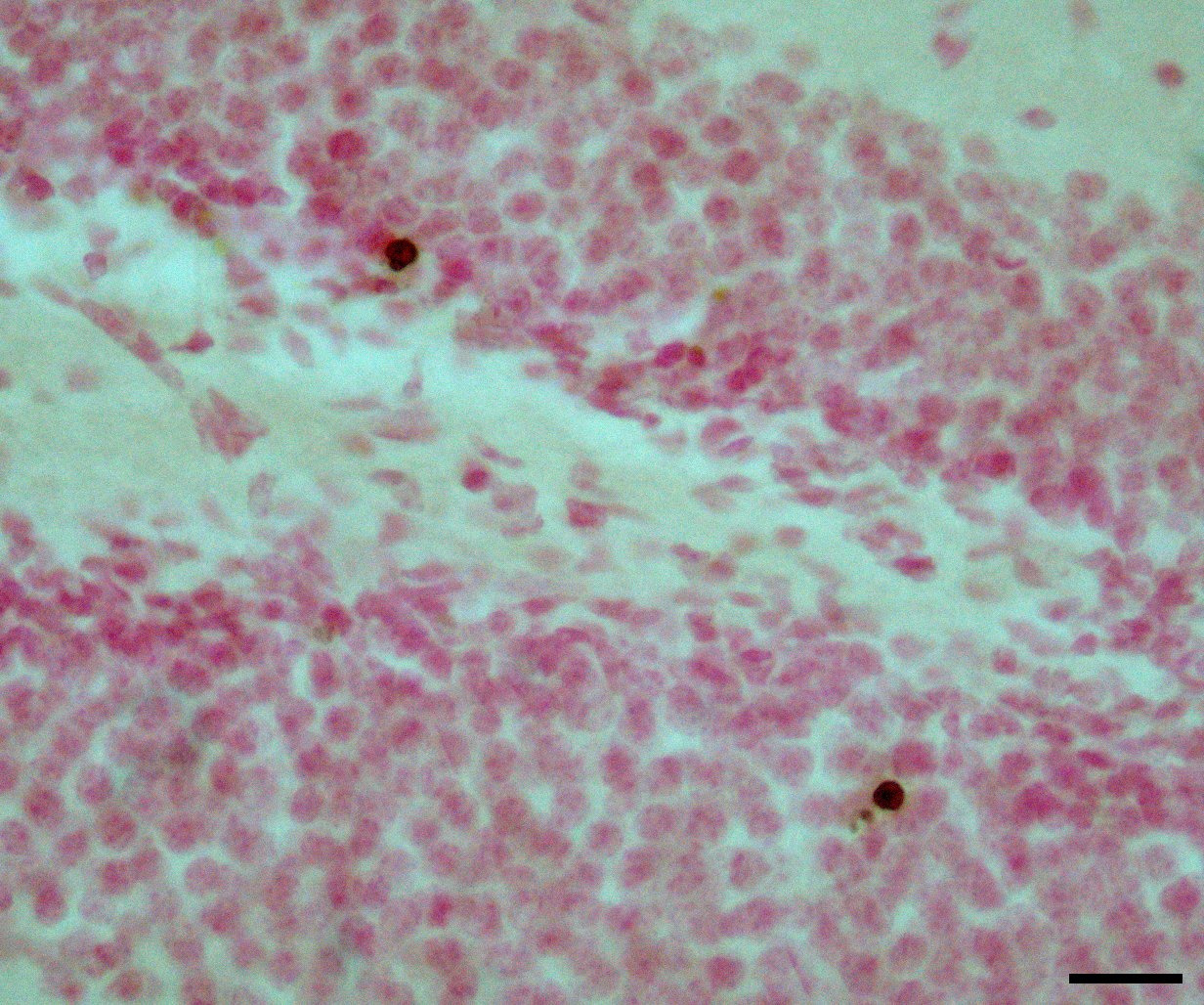

Supplement: Supplementary file 1 [file ijms-27-04356-s001.zip › Supplementary Figures/Supplementary Figure S2. CC3/KO Control/40x_CC3_scalebar.jpg]

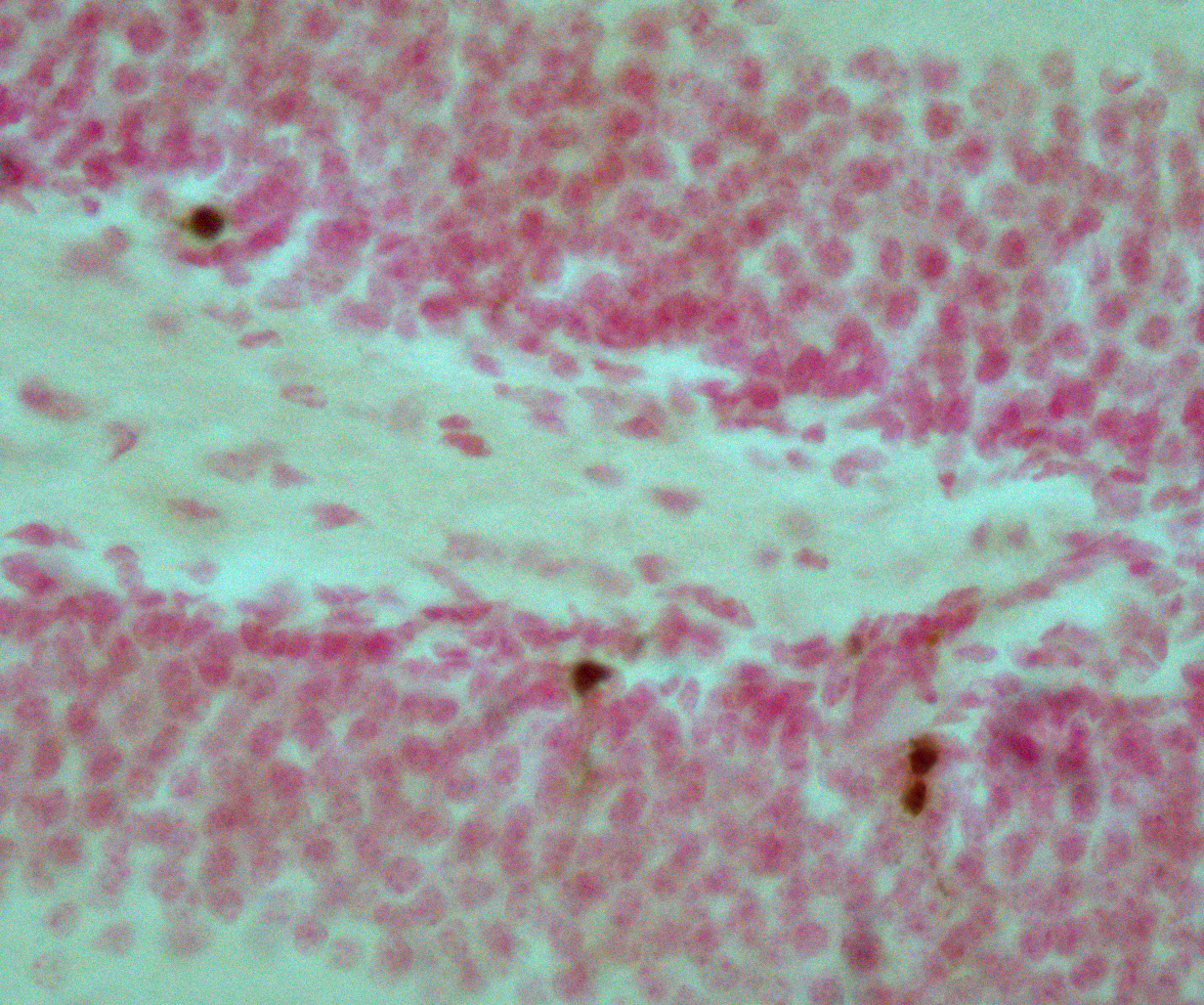

Supplement: Supplementary file 1 [file ijms-27-04356-s001.zip › Supplementary Figures/Supplementary Figure S2. CC3/KO Stress/40x_CC3.tif]

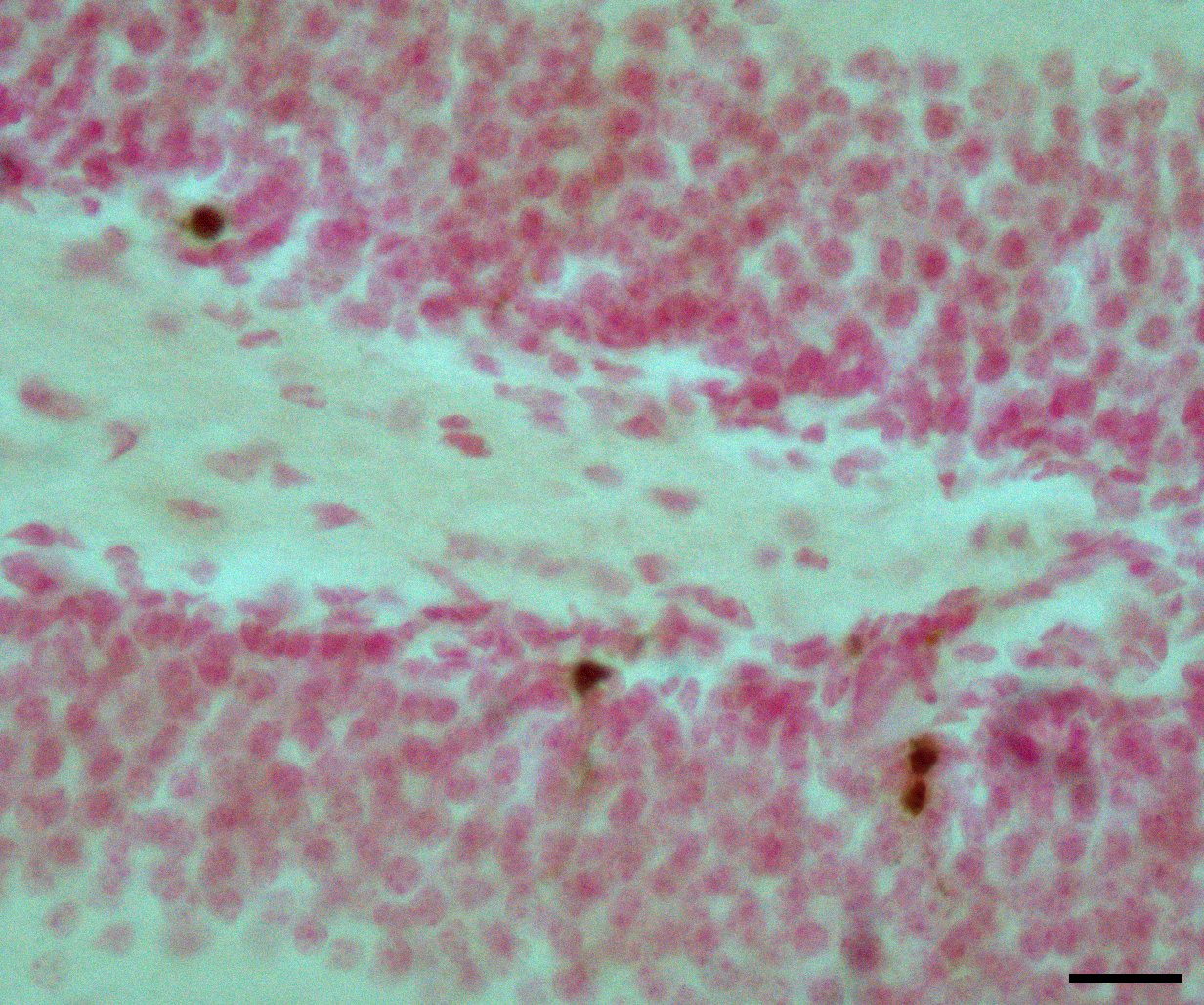

Supplement: Supplementary file 1 [file ijms-27-04356-s001.zip › Supplementary Figures/Supplementary Figure S2. CC3/KO Stress/40x_CC3_scalebar.jpg]

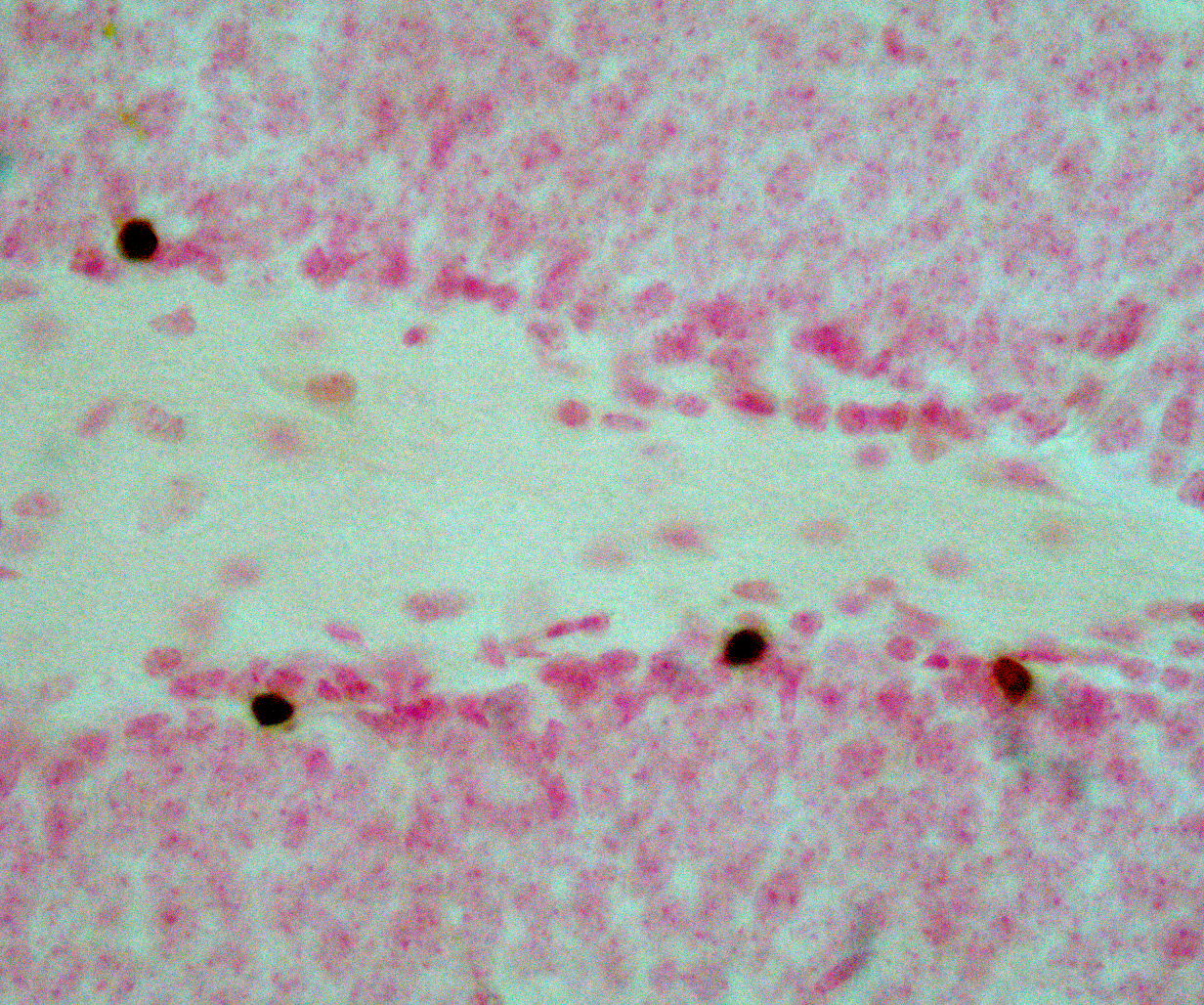

Supplement: Supplementary file 1 [file ijms-27-04356-s001.zip › Supplementary Figures/Supplementary Figure S2. CC3/WT Control/40x_CC3.tif]

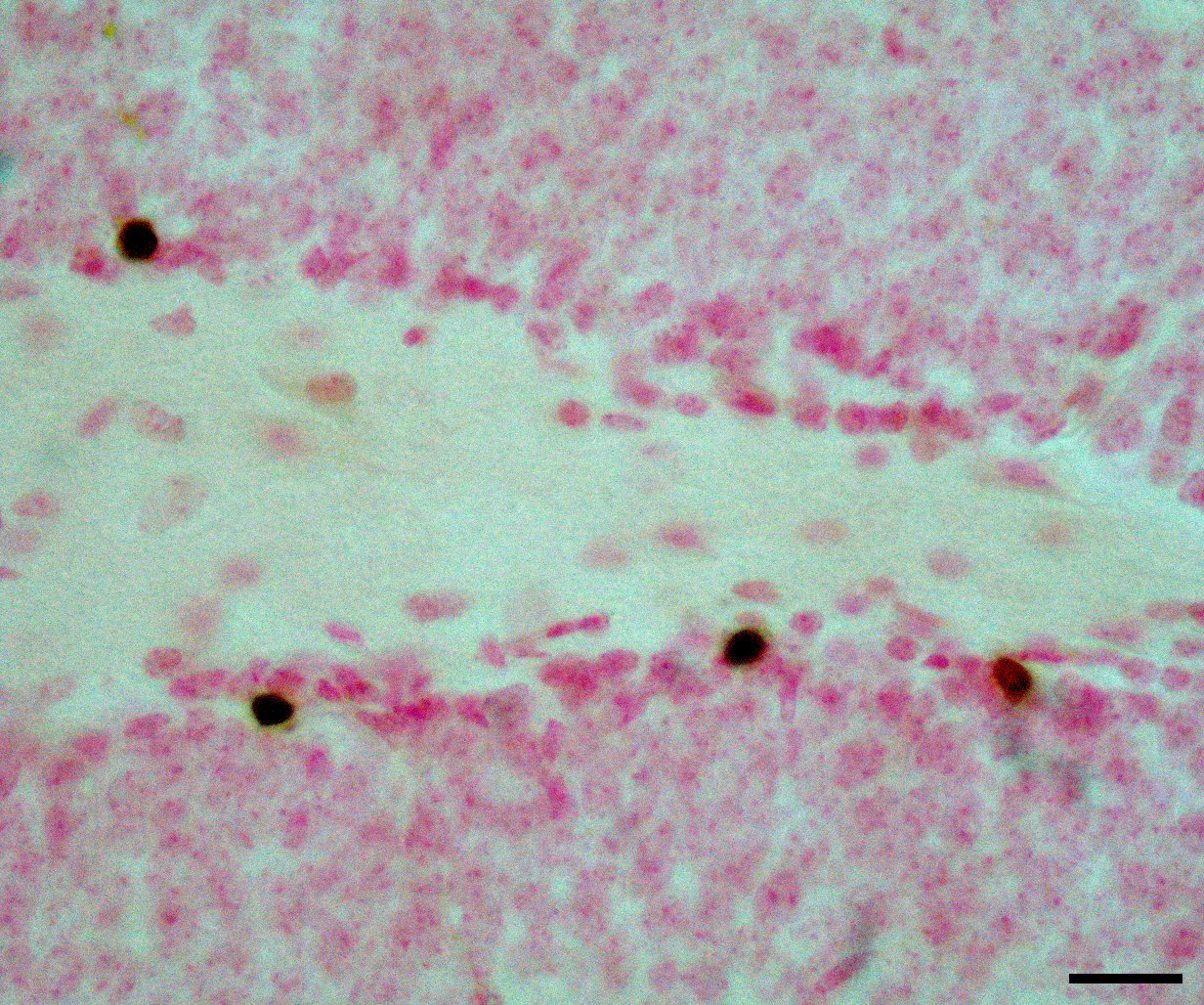

Supplement: Supplementary file 1 [file ijms-27-04356-s001.zip › Supplementary Figures/Supplementary Figure S2. CC3/WT Control/40x_CC3_scalebar.jpg]

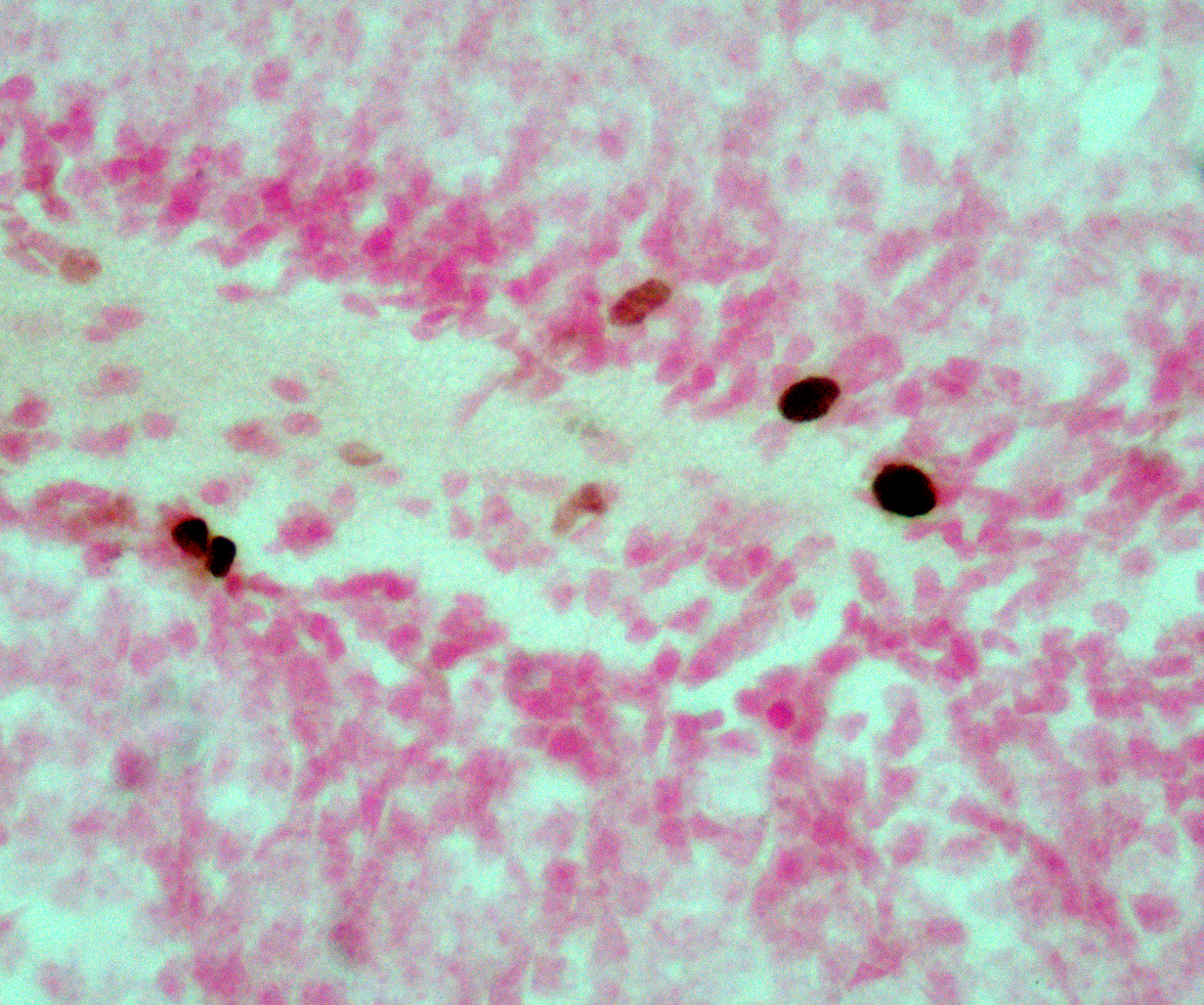

Supplement: Supplementary file 1 [file ijms-27-04356-s001.zip › Supplementary Figures/Supplementary Figure S2. CC3/WT Stress/40x_CC3.tif]

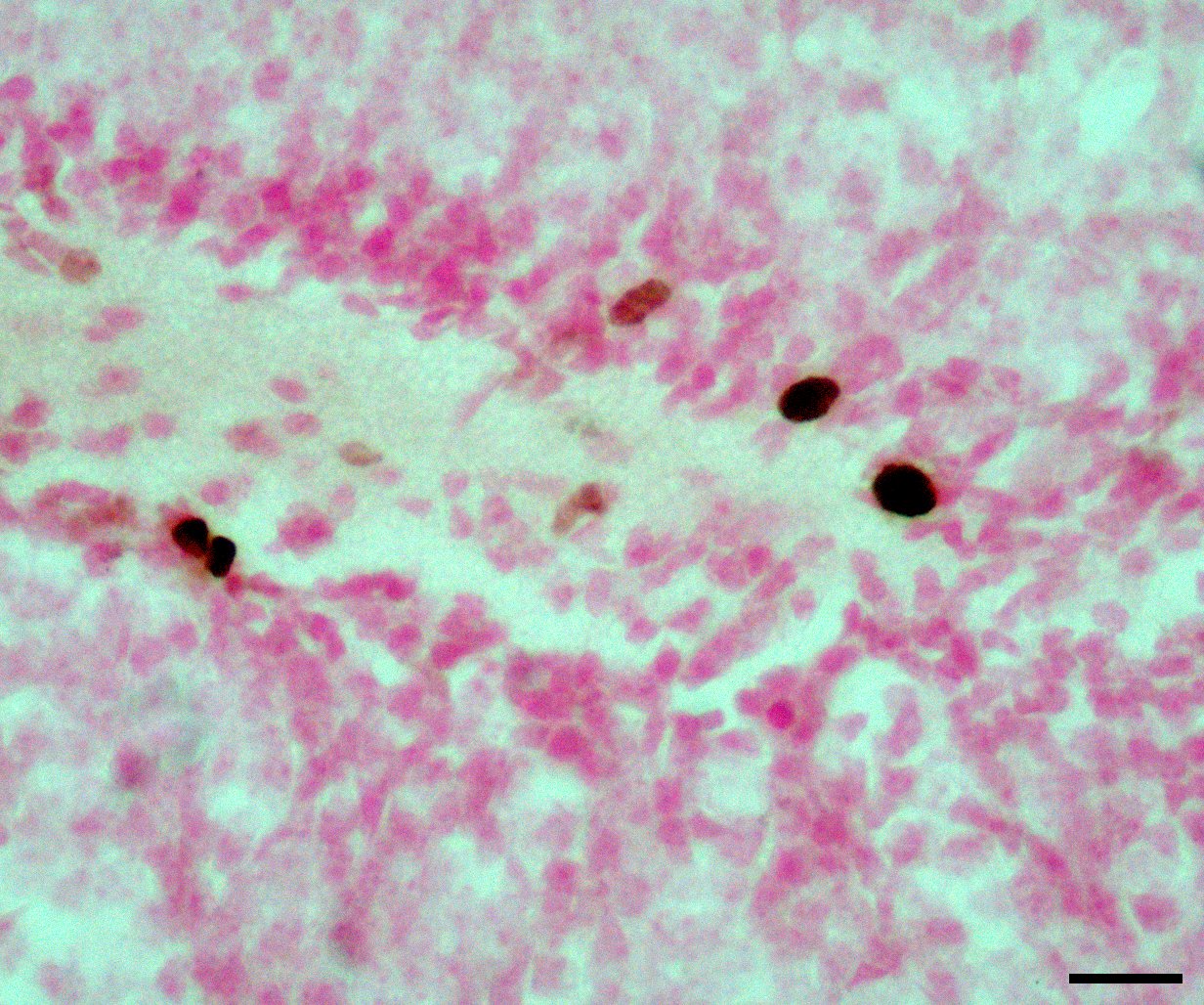

Supplement: Supplementary file 1 [file ijms-27-04356-s001.zip › Supplementary Figures/Supplementary Figure S2. CC3/WT Stress/40x_CC3_scalebar.jpg]

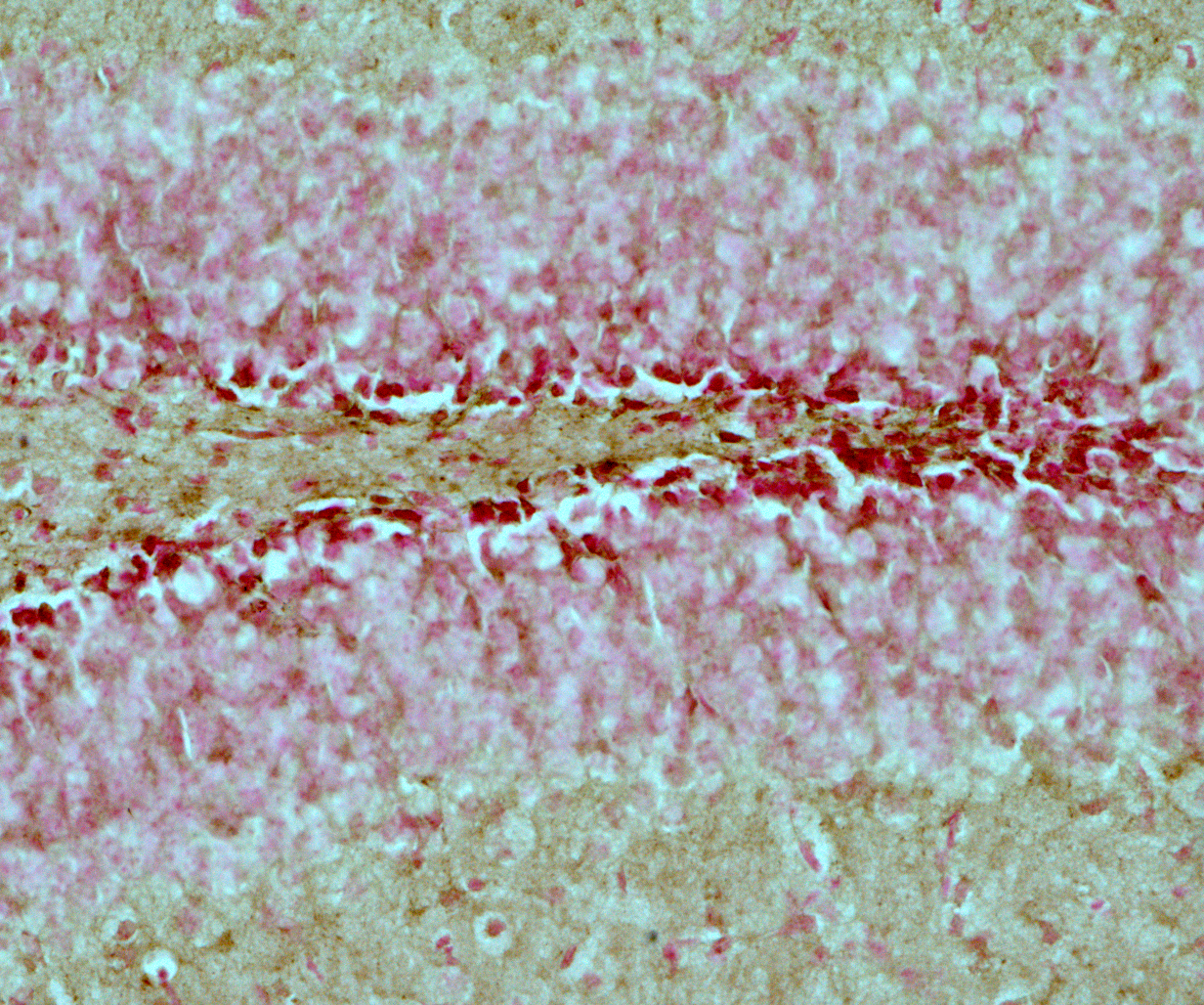

Supplement: Supplementary file 1 [file ijms-27-04356-s001.zip › Supplementary Figures/Supplementary Figure S3. DCX/DCX KO Control/20x_DCX.tif]

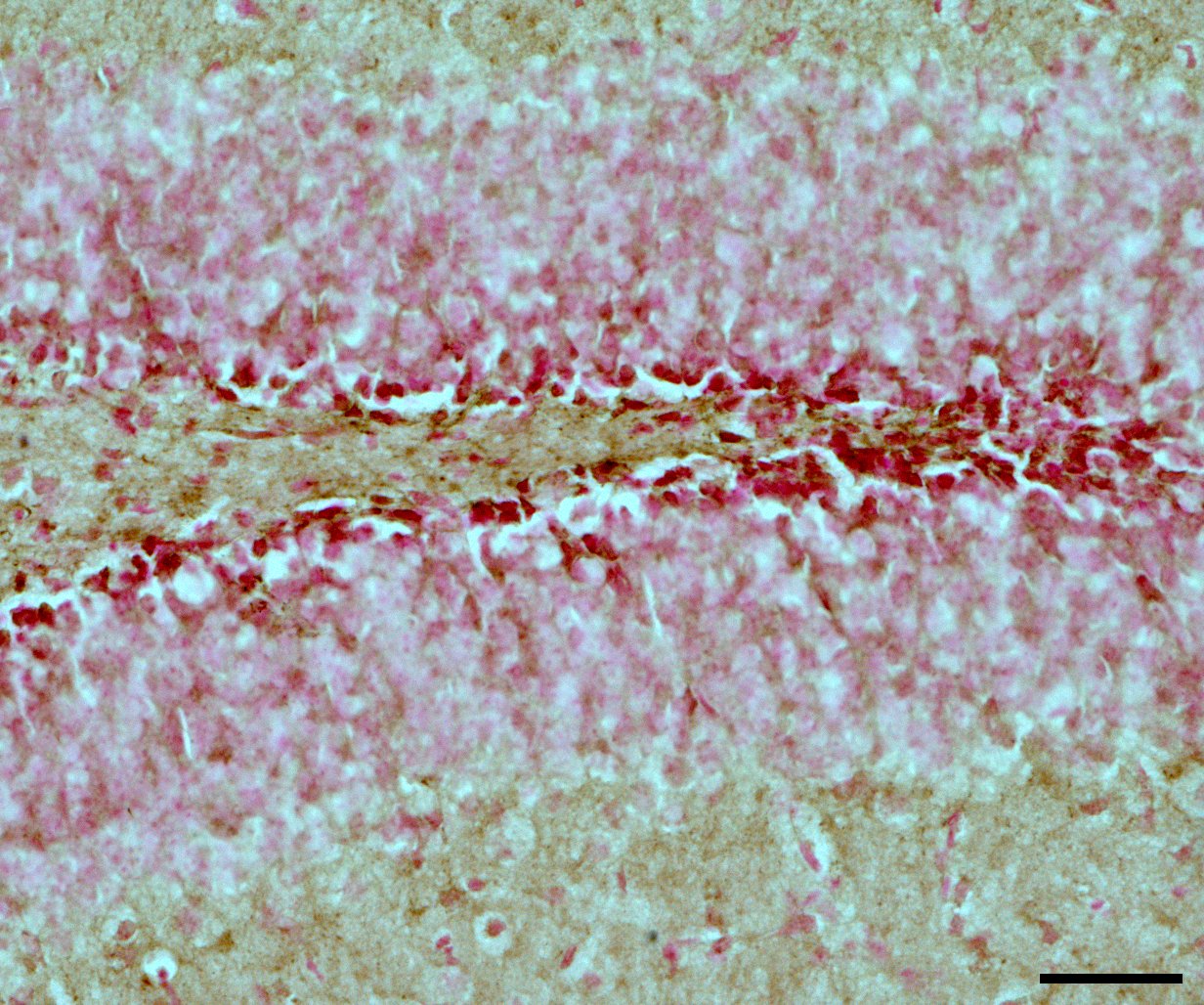

Supplement: Supplementary file 1 [file ijms-27-04356-s001.zip › Supplementary Figures/Supplementary Figure S3. DCX/DCX KO Control/20x_DCX_scalebar.jpg]

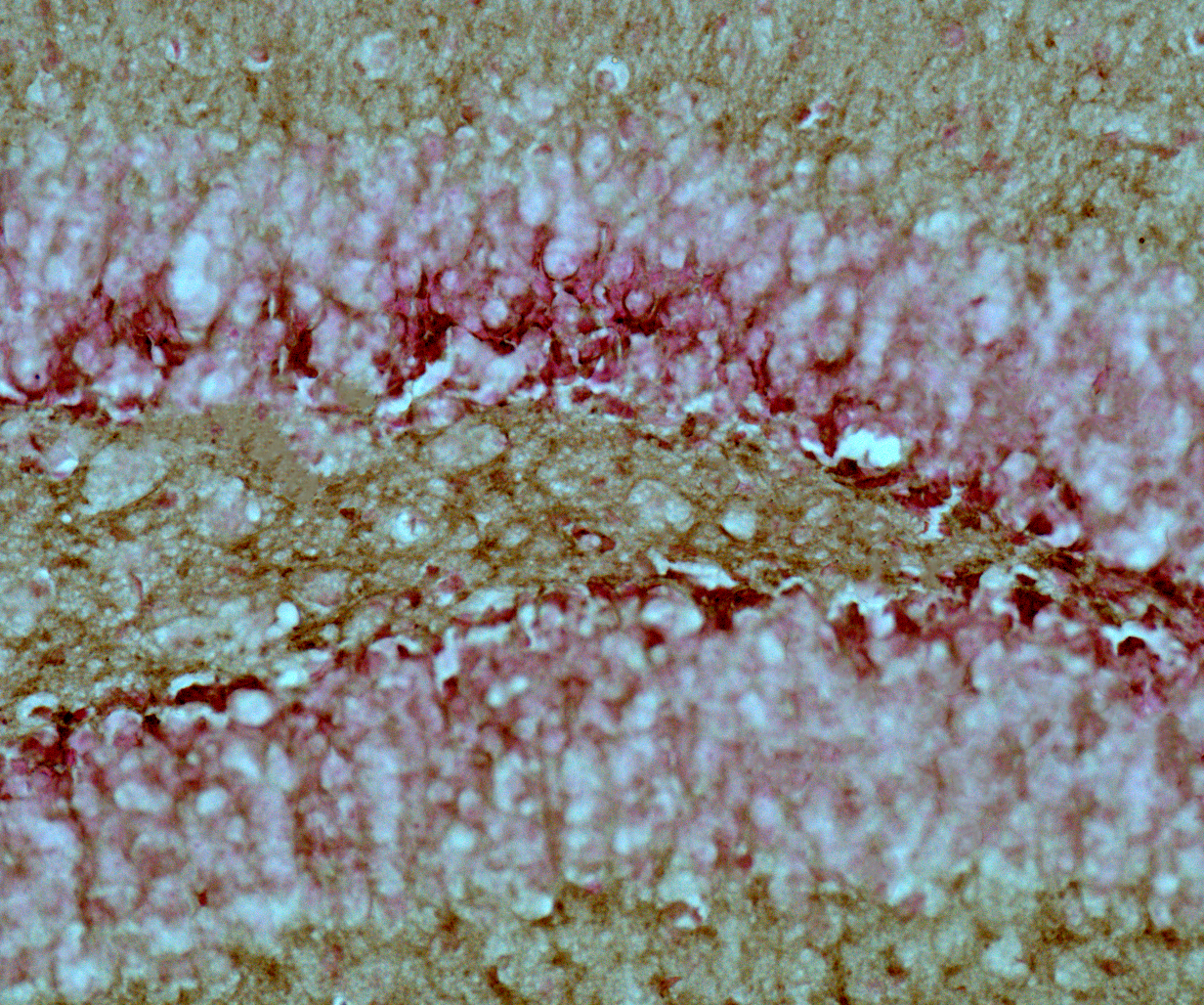

Supplement: Supplementary file 1 [file ijms-27-04356-s001.zip › Supplementary Figures/Supplementary Figure S3. DCX/DCX KO Stress/20x_DCX.tif]

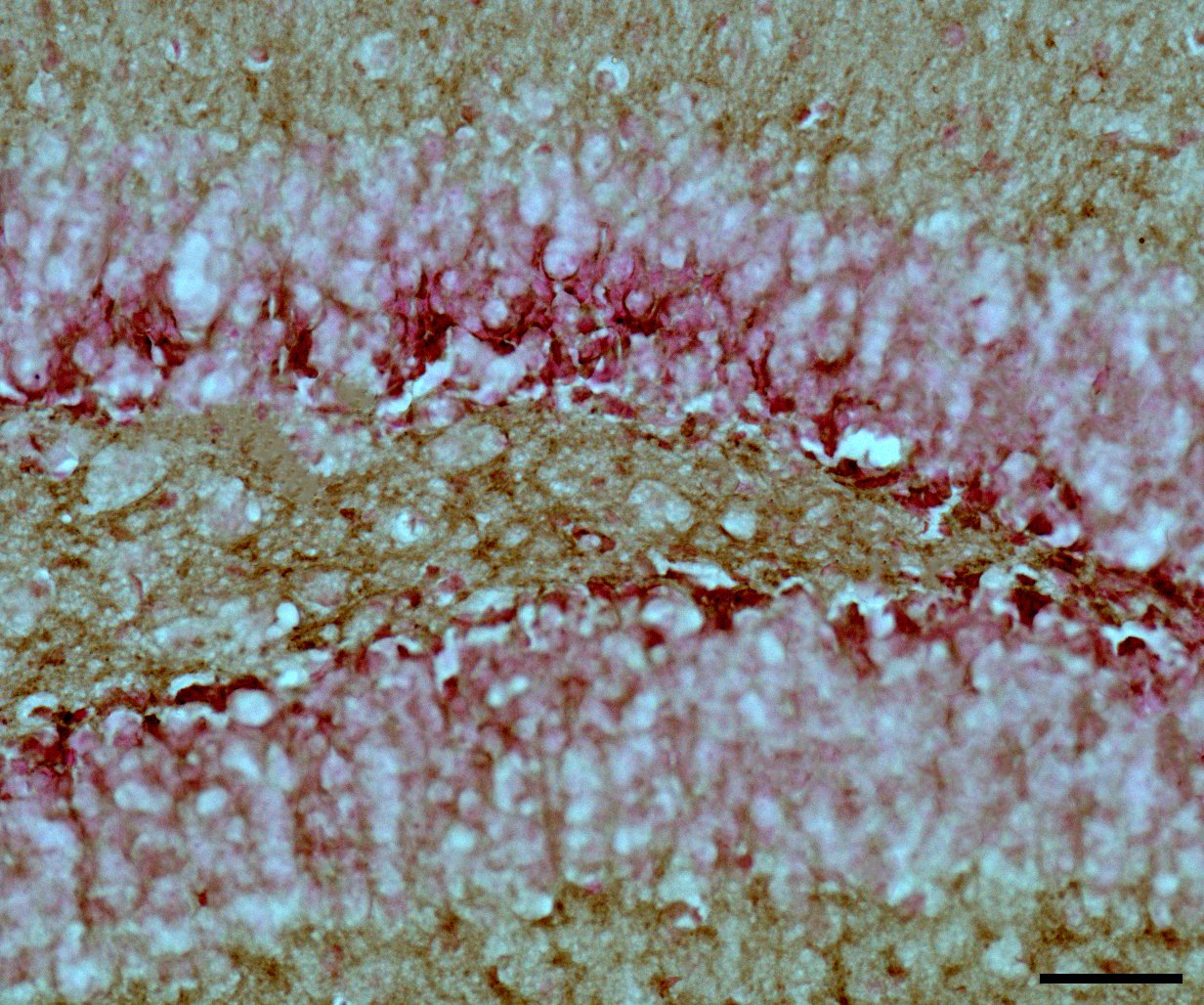

Supplement: Supplementary file 1 [file ijms-27-04356-s001.zip › Supplementary Figures/Supplementary Figure S3. DCX/DCX KO Stress/20x_DCX_scalebar.jpg]

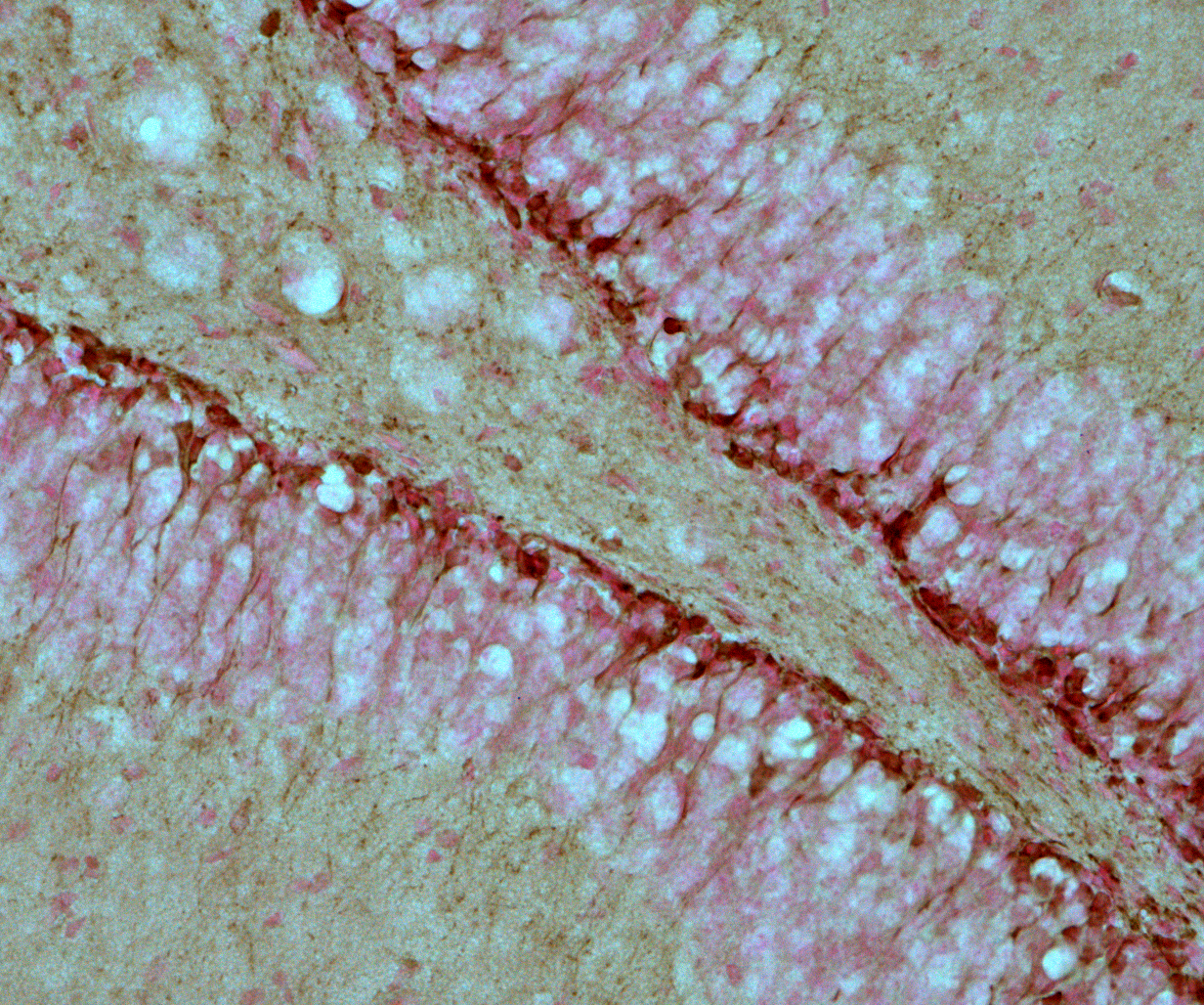

Supplement: Supplementary file 1 [file ijms-27-04356-s001.zip › Supplementary Figures/Supplementary Figure S3. DCX/DCX WT Control/20x_DCX.tif]

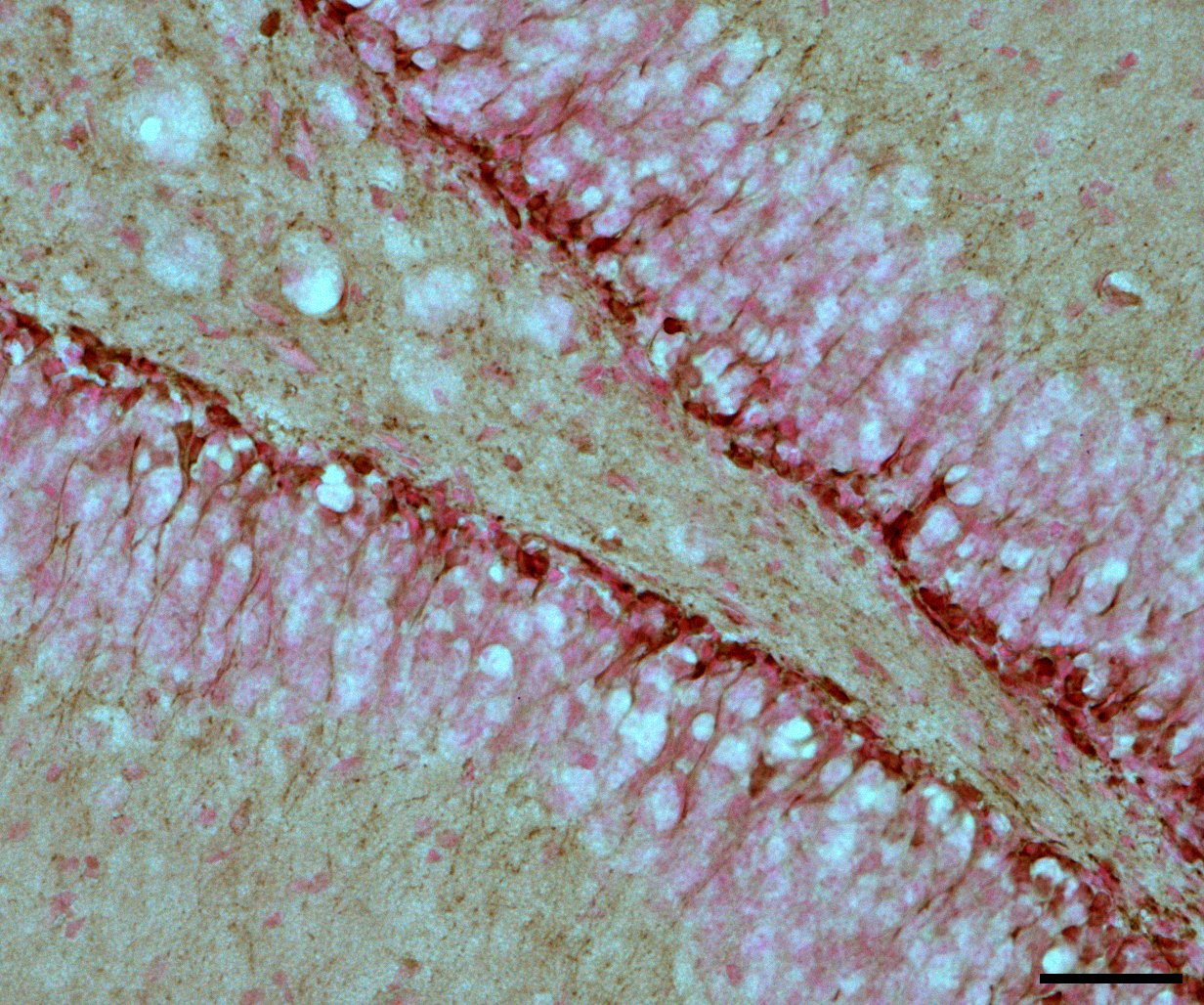

Supplement: Supplementary file 1 [file ijms-27-04356-s001.zip › Supplementary Figures/Supplementary Figure S3. DCX/DCX WT Control/20x_DCX_scalebar.jpg]

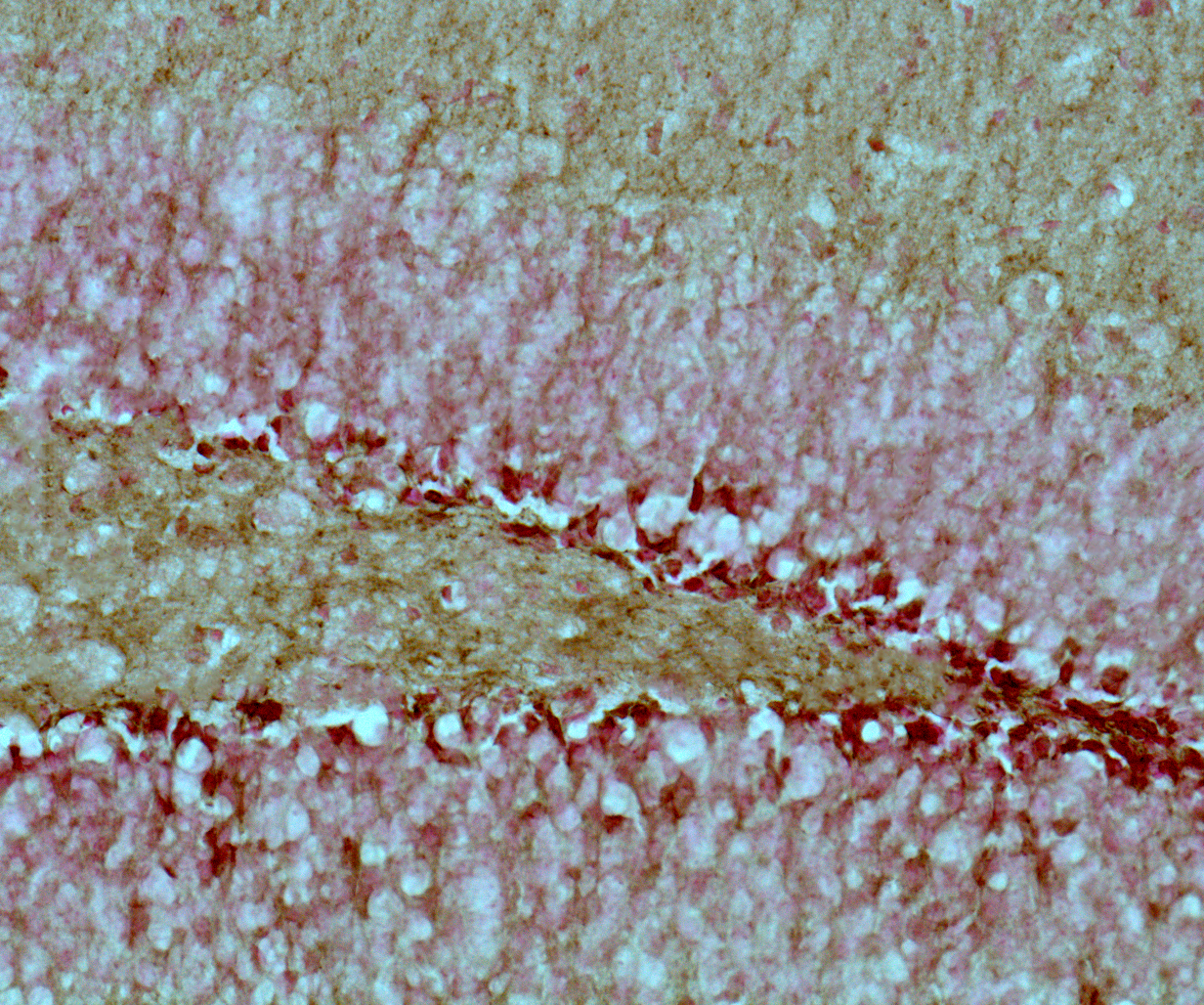

Supplement: Supplementary file 1 [file ijms-27-04356-s001.zip › Supplementary Figures/Supplementary Figure S3. DCX/DCX WT Stress/20x_DCX.tif]

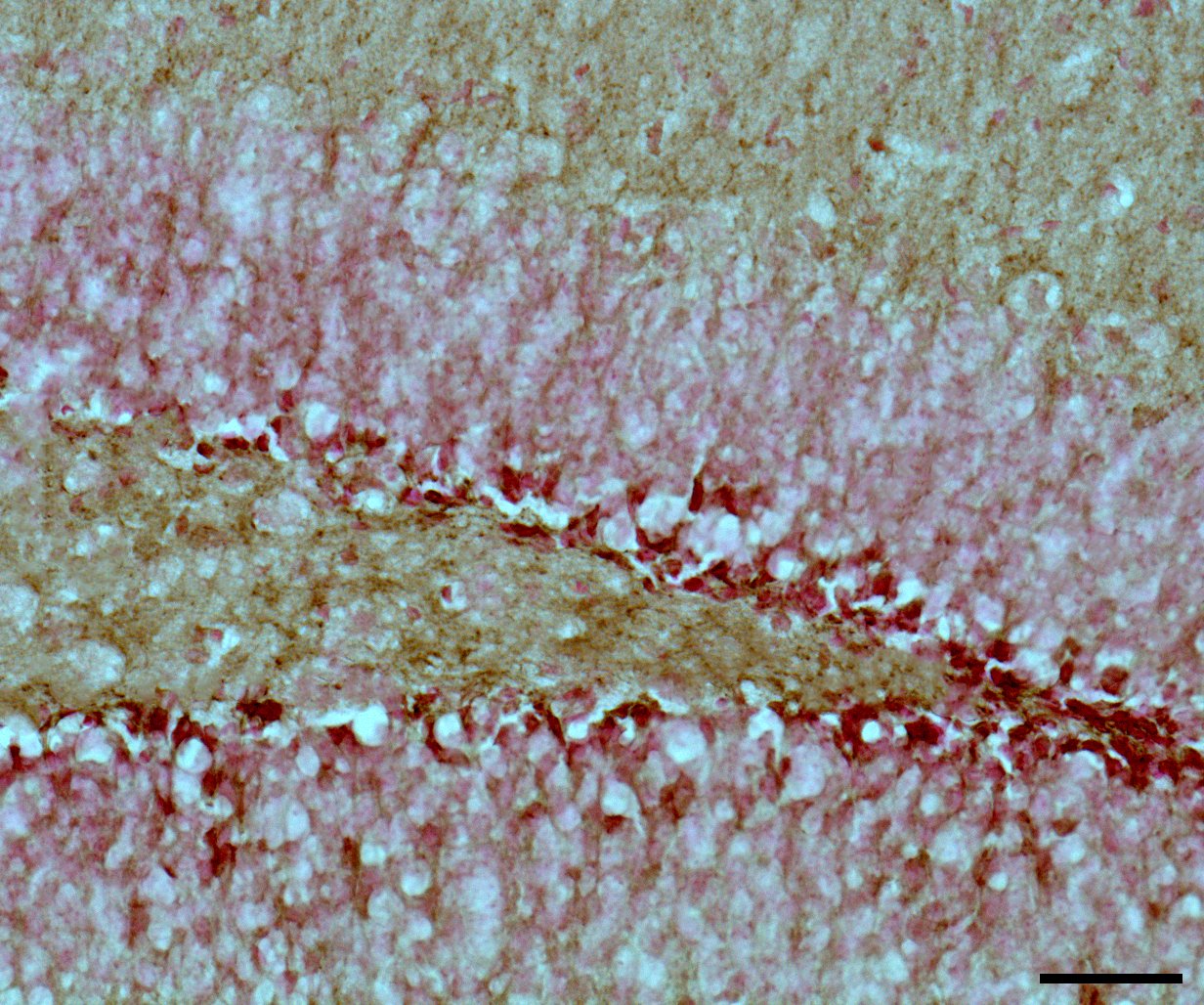

Supplement: Supplementary file 1 [file ijms-27-04356-s001.zip › Supplementary Figures/Supplementary Figure S3. DCX/DCX WT Stress/20x_DCX_scalebar.jpg]

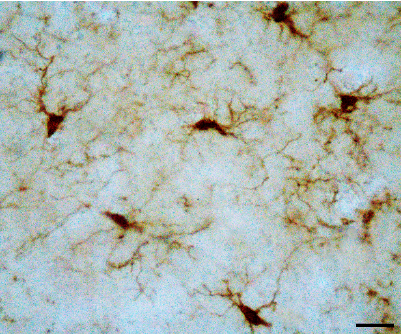

Supplement: Supplementary file 1 [file ijms-27-04356-s001.zip › Supplementary Figures/Supplementary Figure S4. Iba1/40x_Iba1_Fig.4E_Nearest_Neighbor.tif]

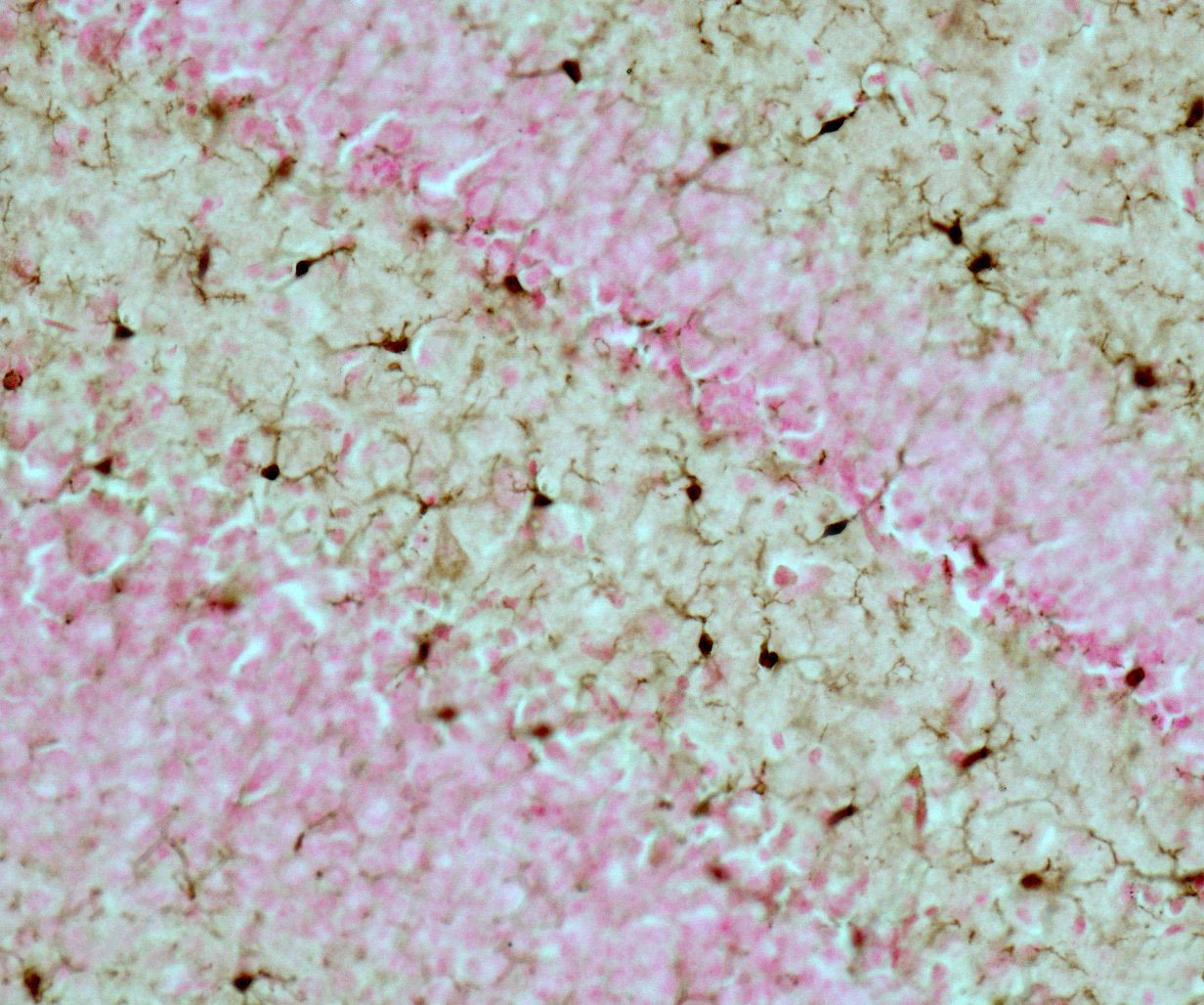

Supplement: Supplementary file 1 [file ijms-27-04356-s001.zip › Supplementary Figures/Supplementary Figure S4. Iba1/KO Control/20x_Iba1.tif]

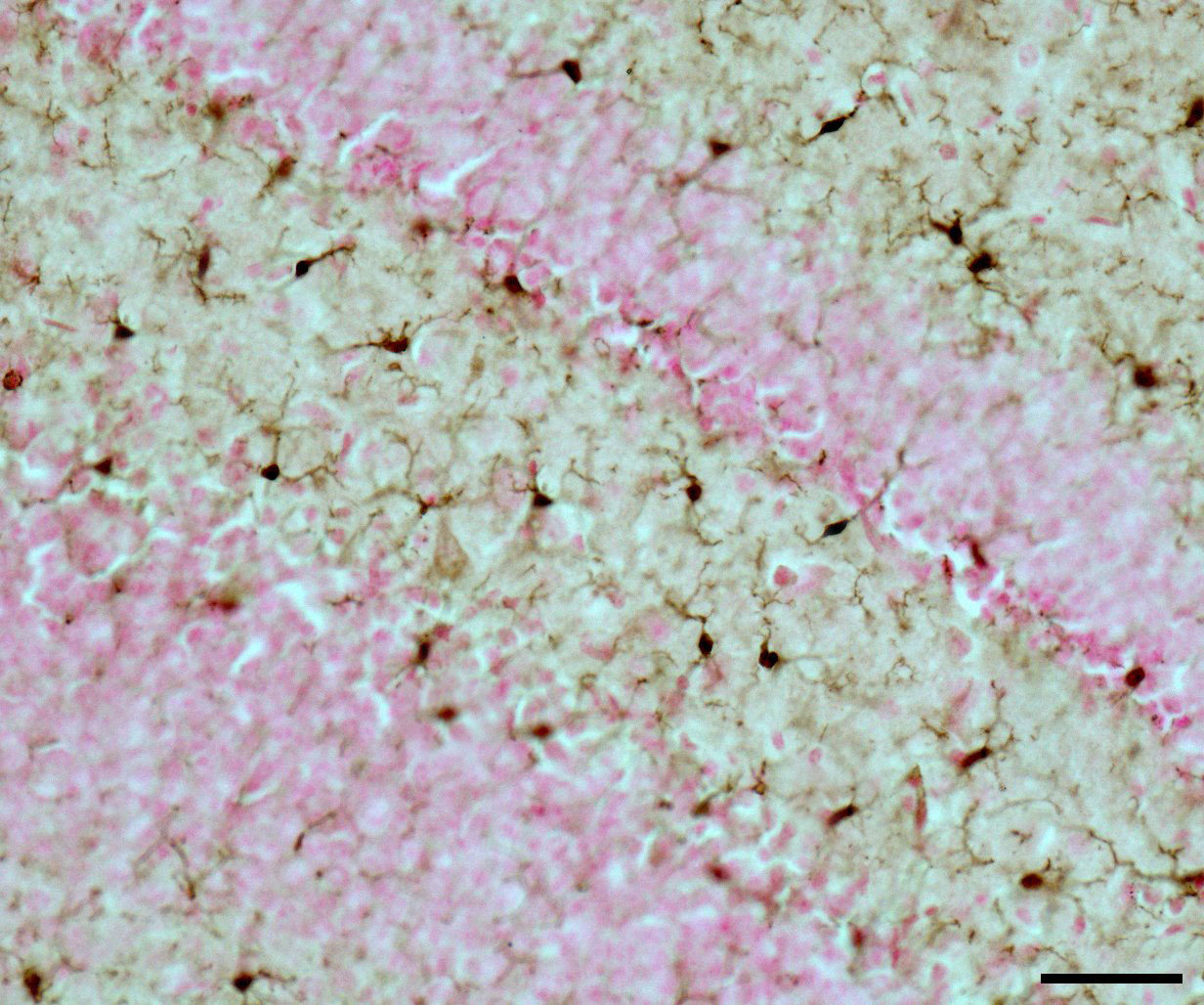

Supplement: Supplementary file 1 [file ijms-27-04356-s001.zip › Supplementary Figures/Supplementary Figure S4. Iba1/KO Control/20x_Iba1_scalebar.jpg]

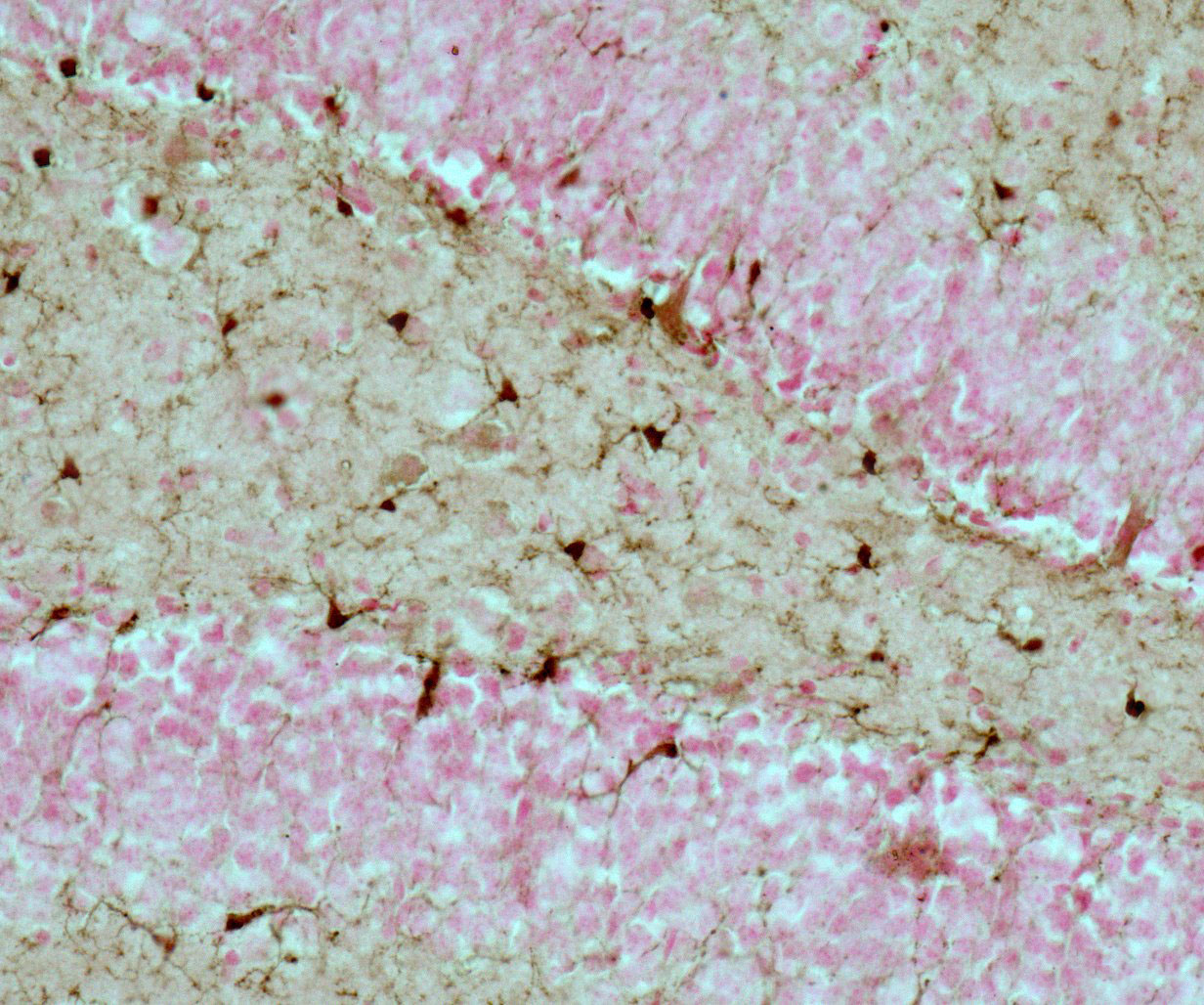

Supplement: Supplementary file 1 [file ijms-27-04356-s001.zip › Supplementary Figures/Supplementary Figure S4. Iba1/KO Stress/20x_Iba1.tif]

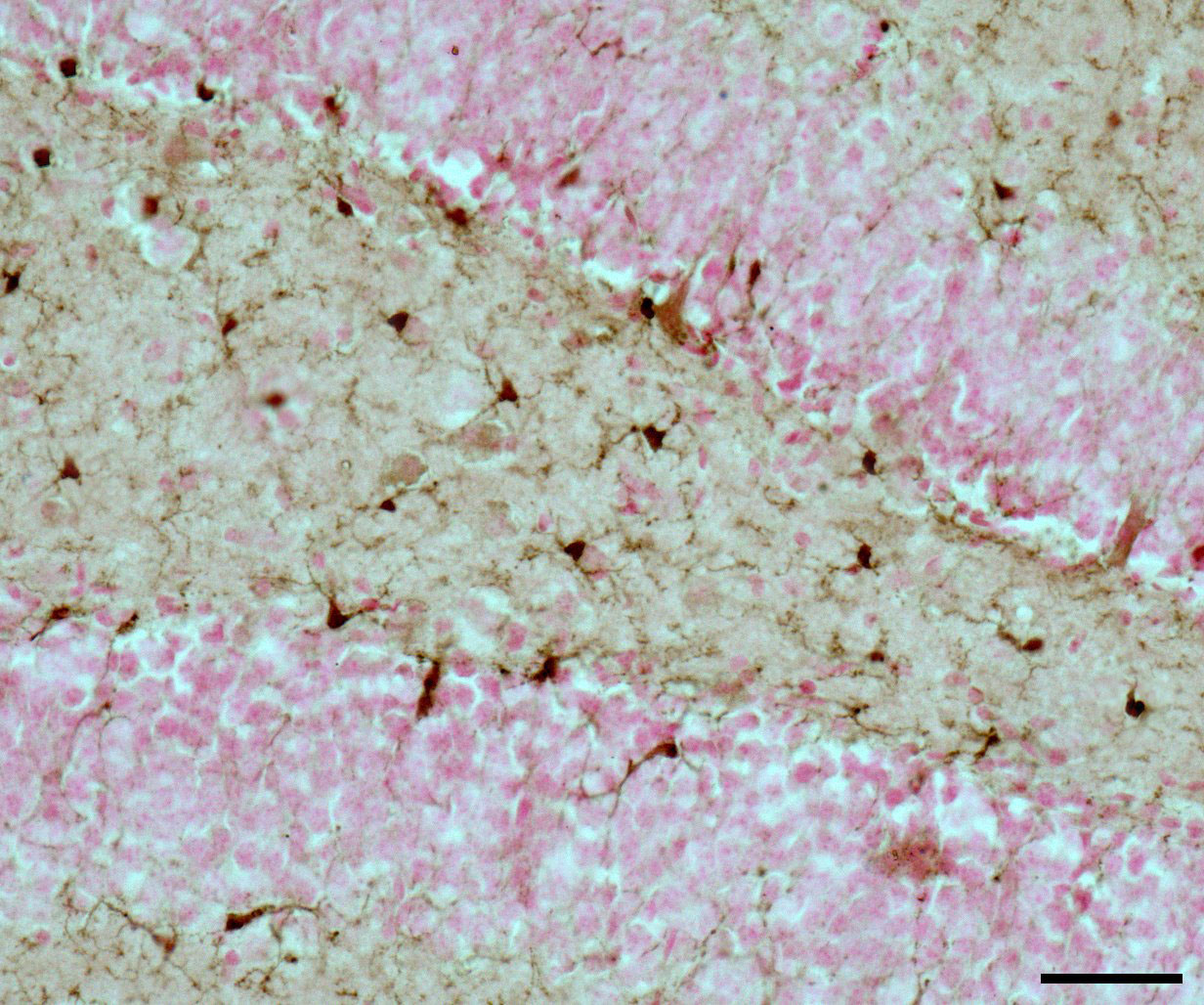

Supplement: Supplementary file 1 [file ijms-27-04356-s001.zip › Supplementary Figures/Supplementary Figure S4. Iba1/KO Stress/20x_Iba1_scalebar.jpg]

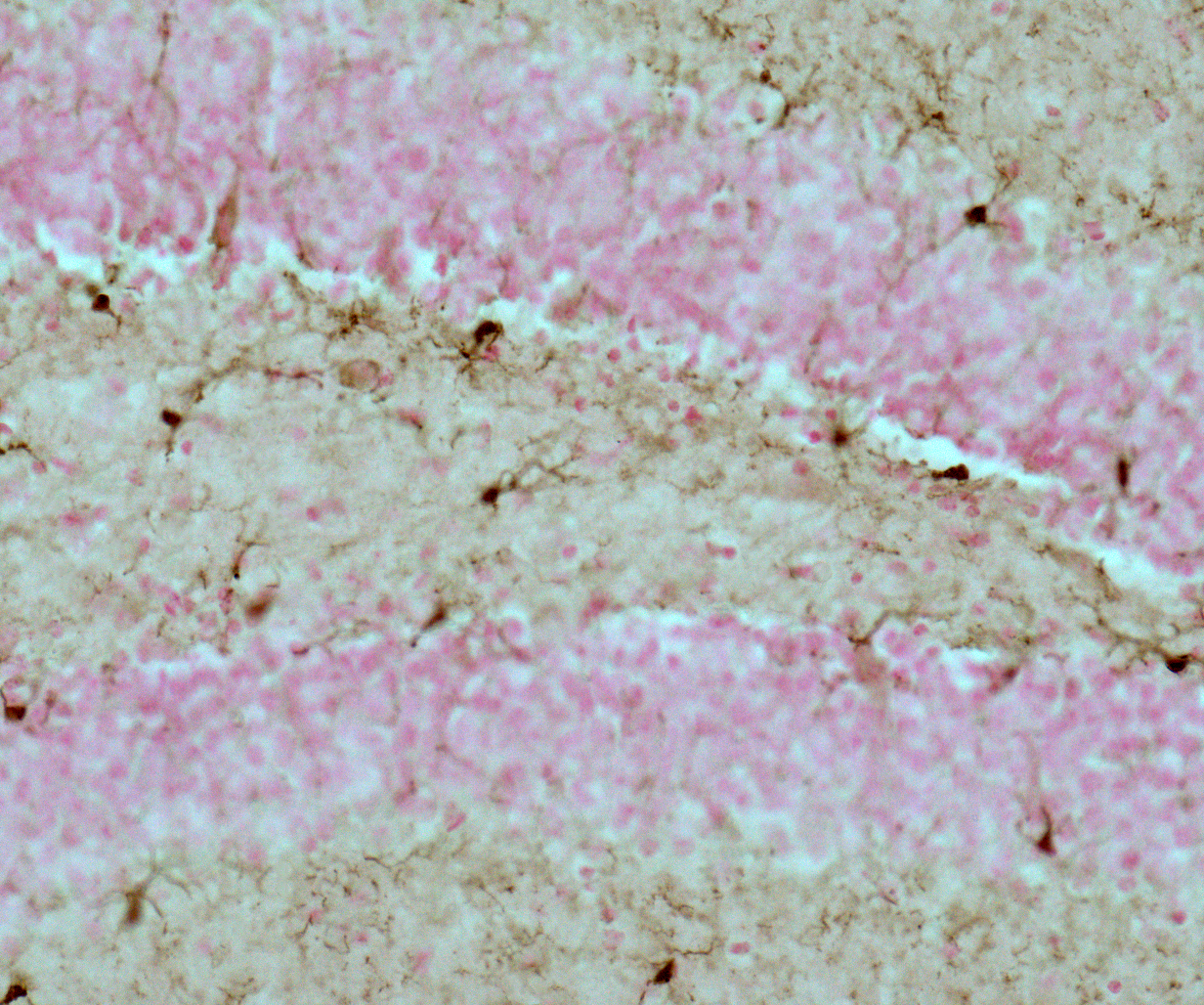

Supplement: Supplementary file 1 [file ijms-27-04356-s001.zip › Supplementary Figures/Supplementary Figure S4. Iba1/WT Control/20x_Iba1.tif]

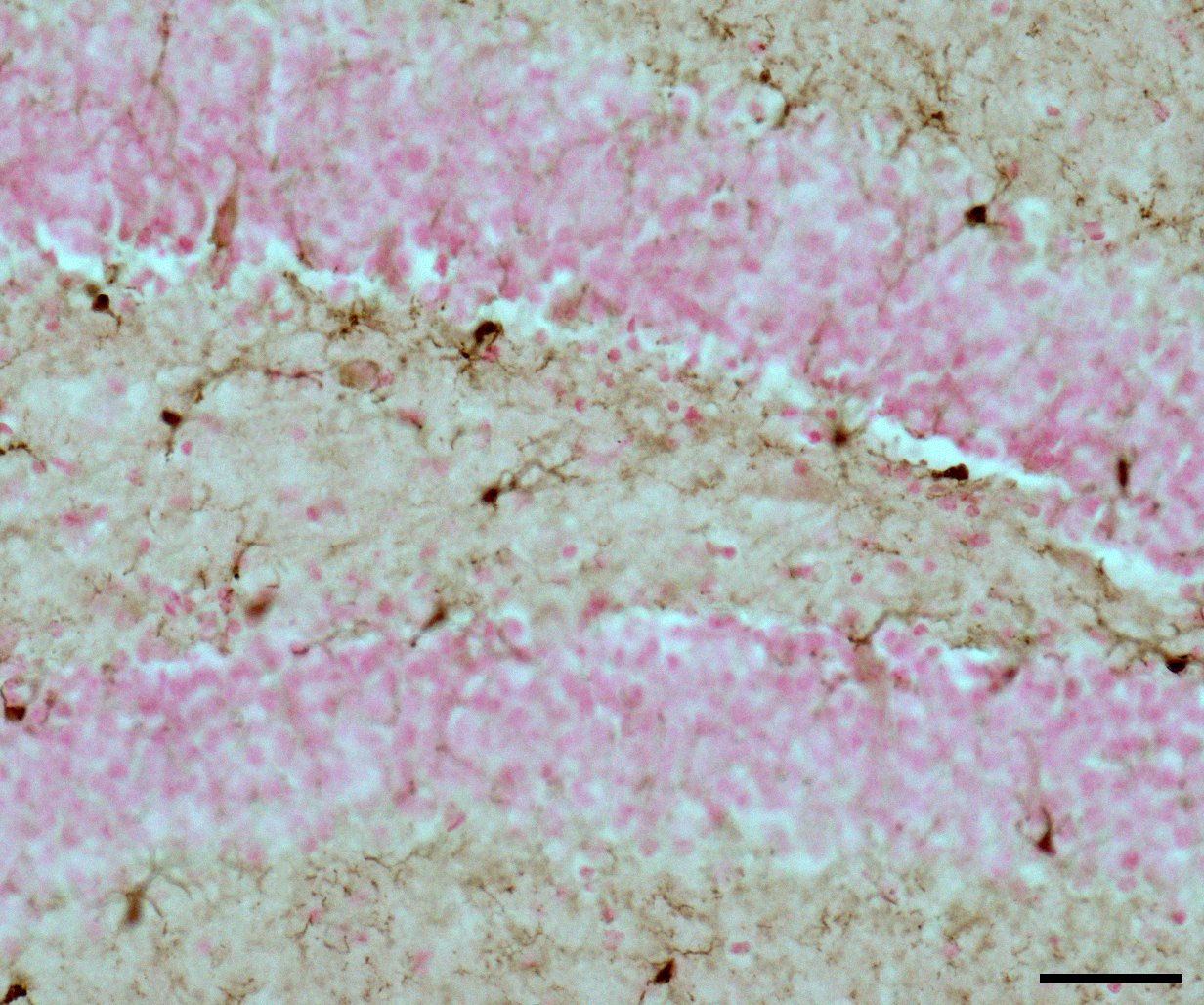

Supplement: Supplementary file 1 [file ijms-27-04356-s001.zip › Supplementary Figures/Supplementary Figure S4. Iba1/WT Control/20x_Iba1_scalebar.jpg]

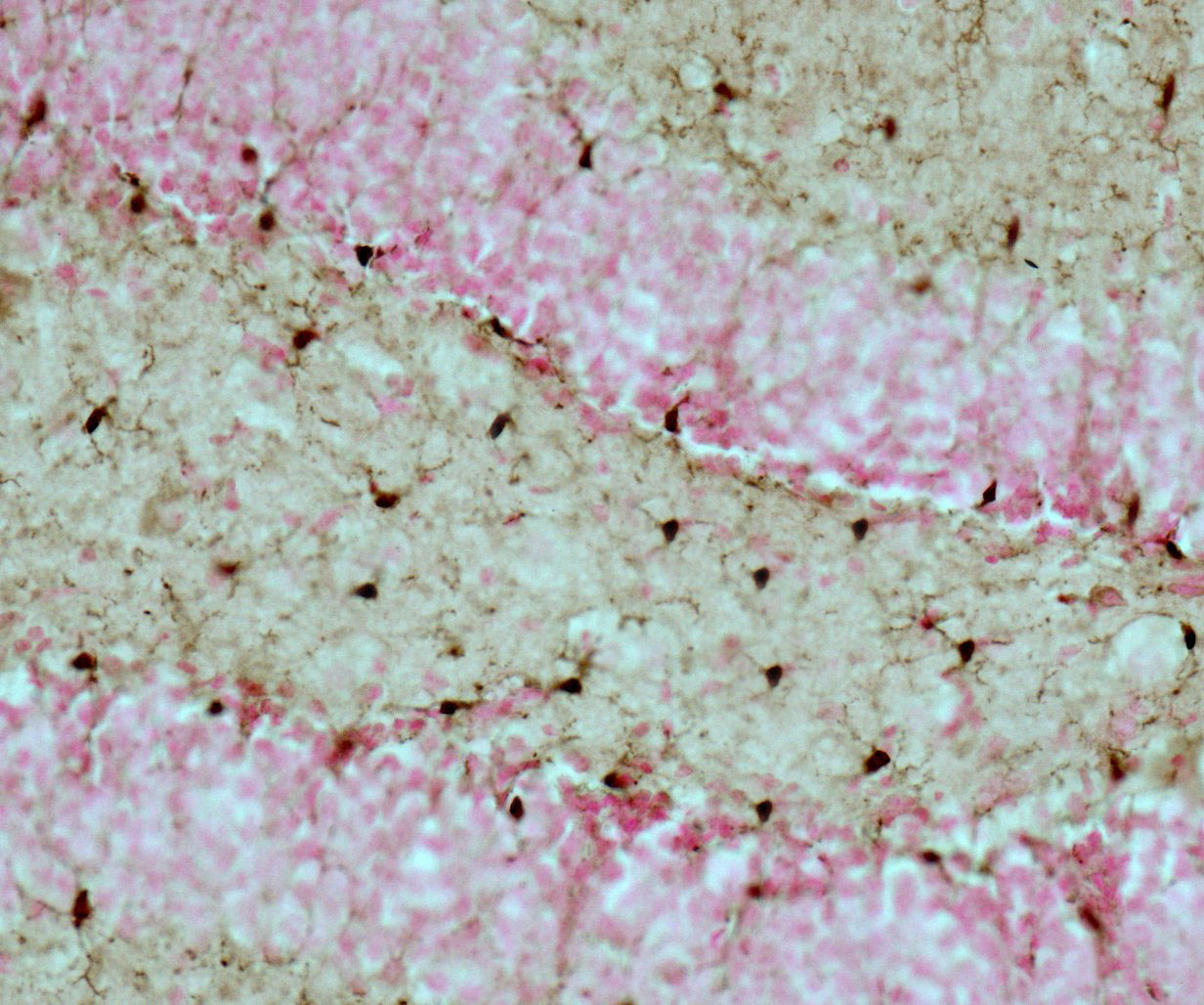

Supplement: Supplementary file 1 [file ijms-27-04356-s001.zip › Supplementary Figures/Supplementary Figure S4. Iba1/WT Stress/20x_Iba1.tif]

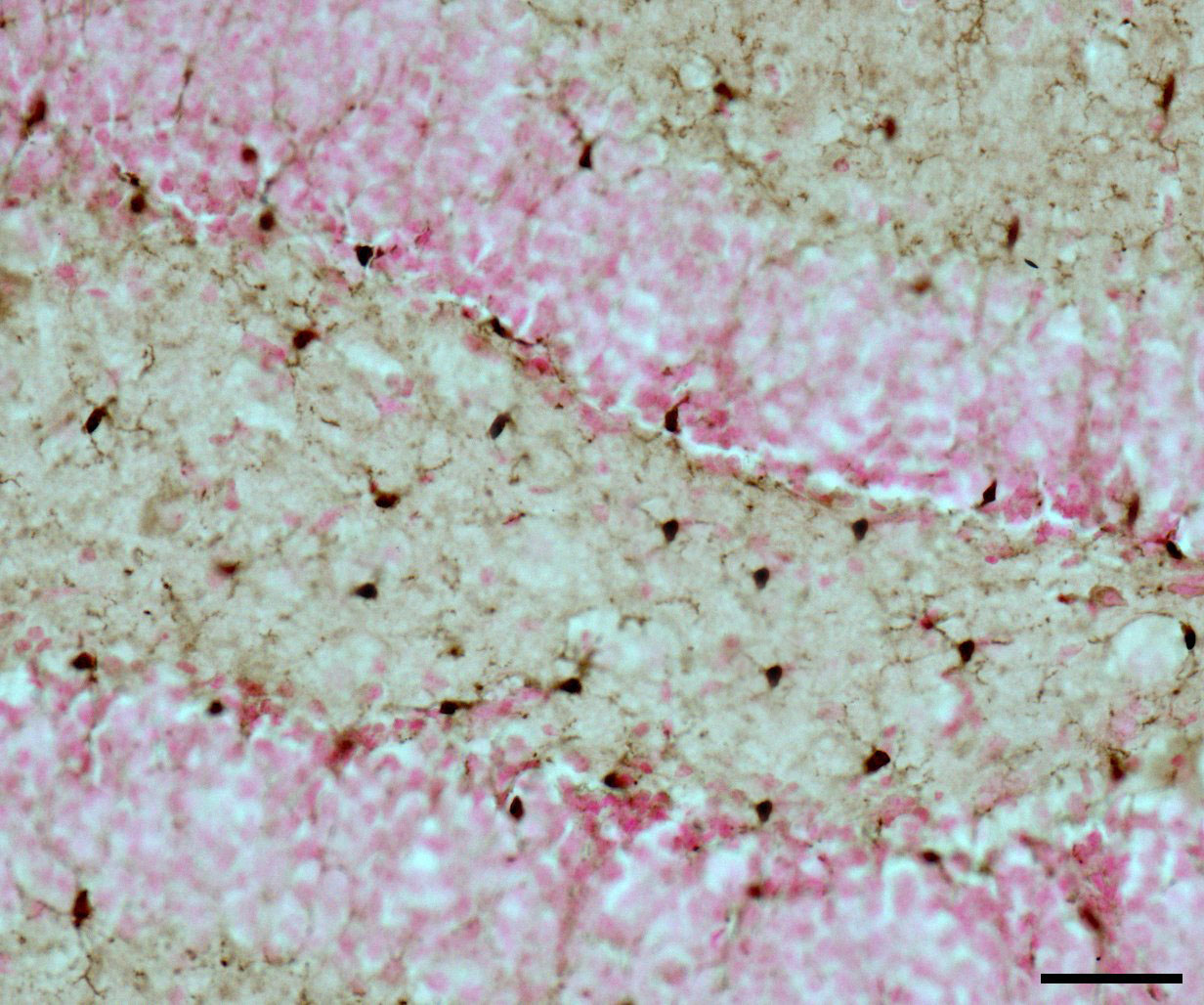

Supplement: Supplementary file 1 [file ijms-27-04356-s001.zip › Supplementary Figures/Supplementary Figure S4. Iba1/WT Stress/20x_Iba1_scalebar.jpg]

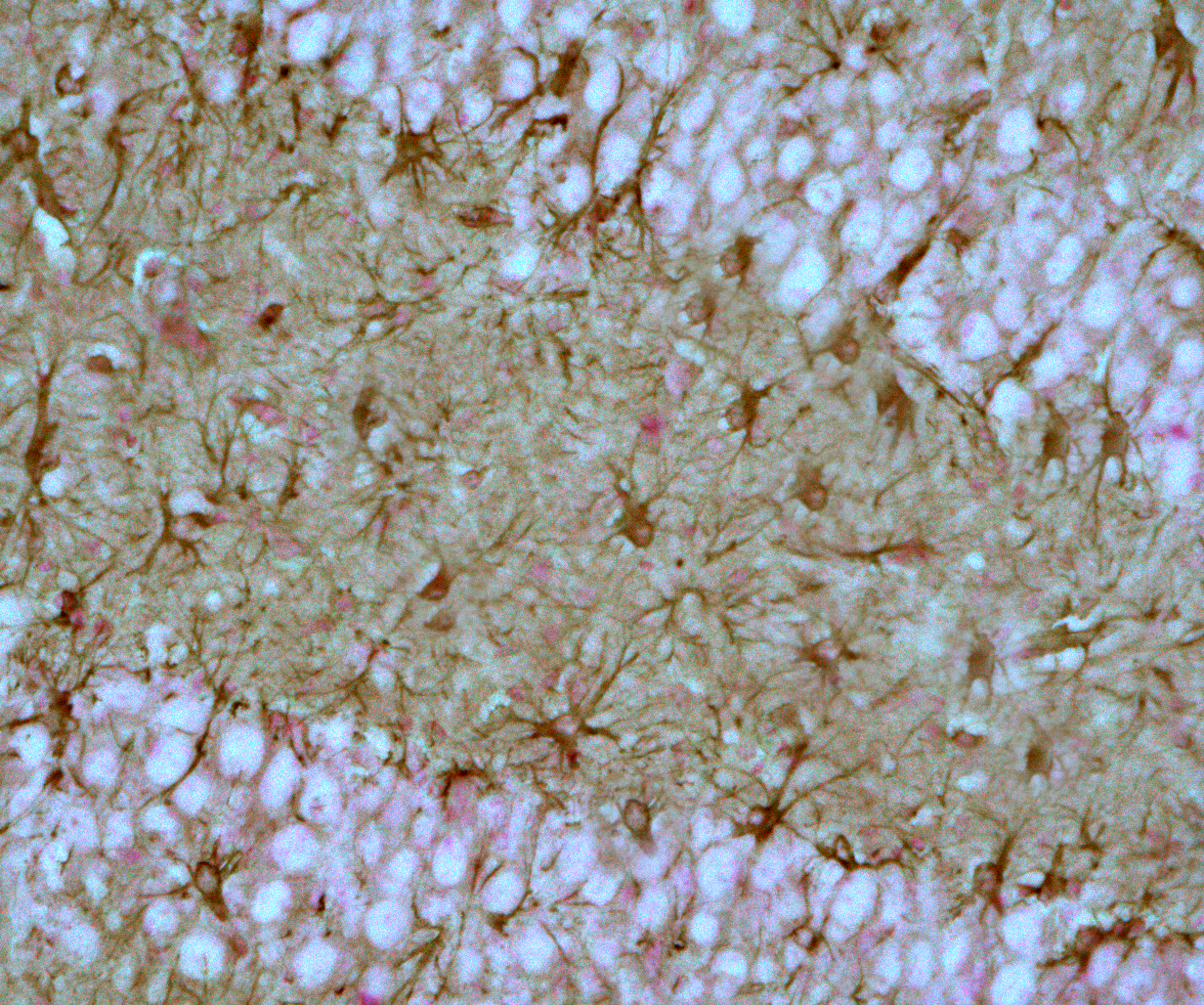

Supplement: Supplementary file 1 [file ijms-27-04356-s001.zip › Supplementary Figures/Supplementary Figure S5. GFAP/KO Control/20x_GFAP.tif]

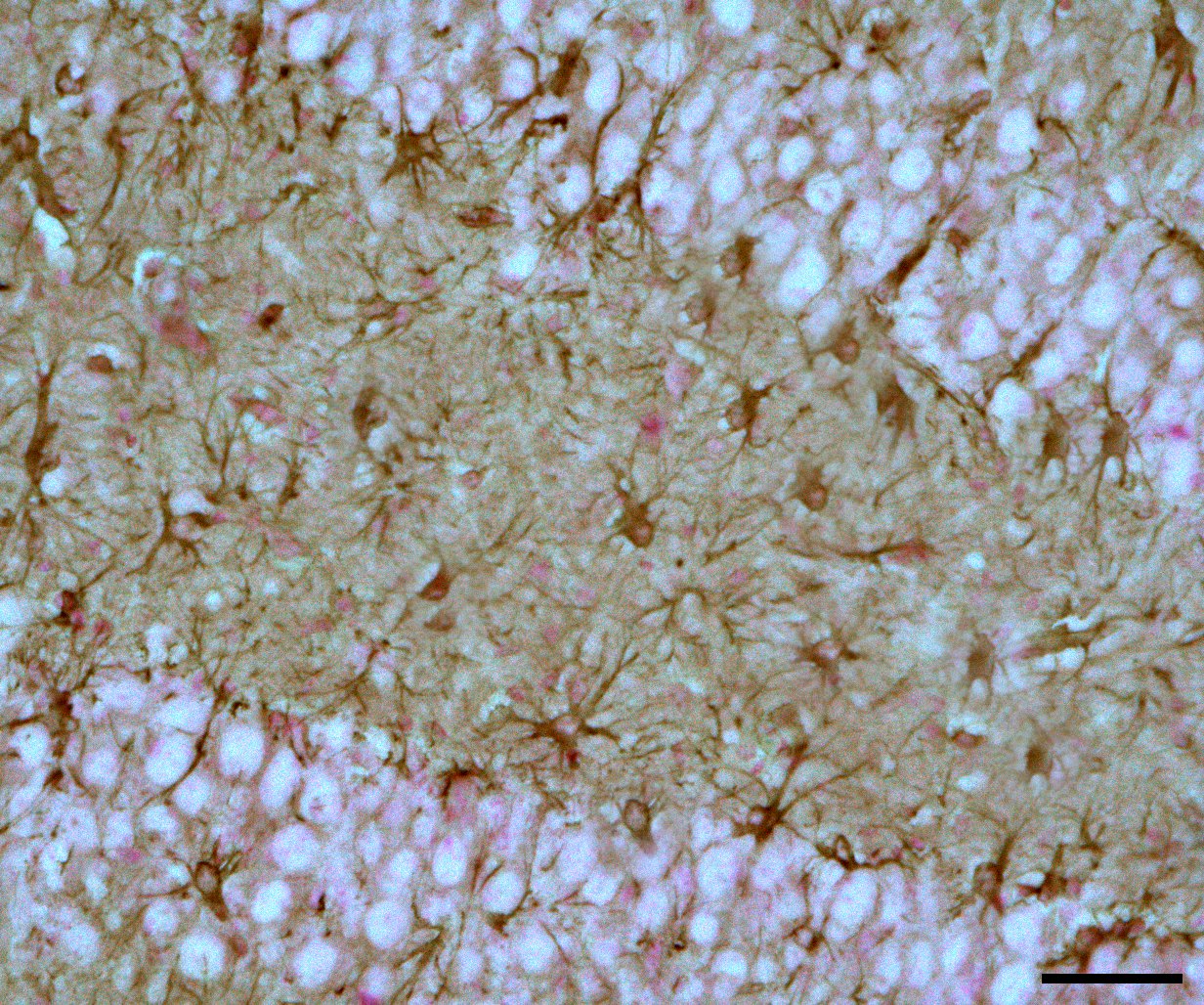

Supplement: Supplementary file 1 [file ijms-27-04356-s001.zip › Supplementary Figures/Supplementary Figure S5. GFAP/KO Control/20x_GFAP_scalebar.jpg]

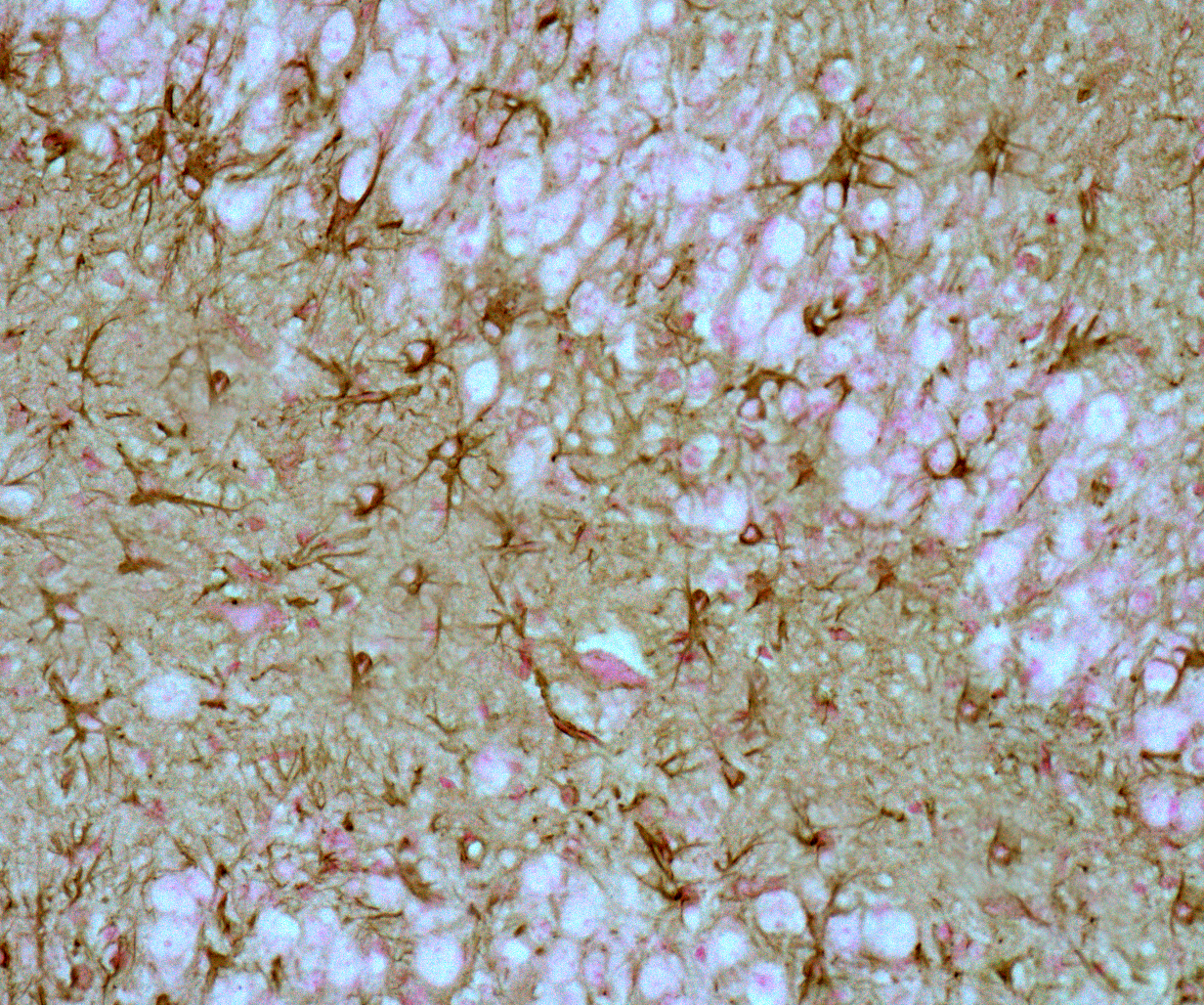

Supplement: Supplementary file 1 [file ijms-27-04356-s001.zip › Supplementary Figures/Supplementary Figure S5. GFAP/KO Stress/20x_GFAP.tif]

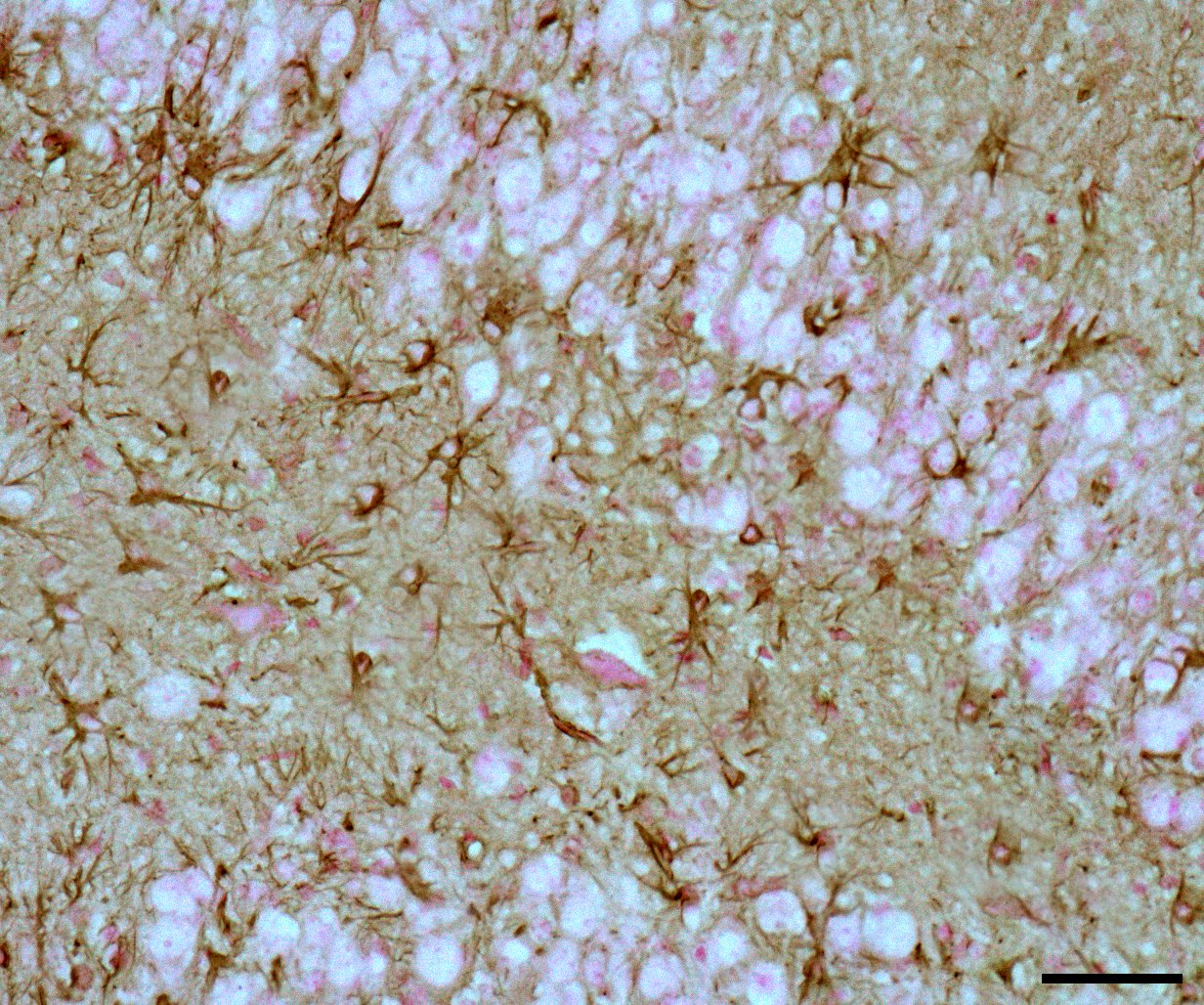

Supplement: Supplementary file 1 [file ijms-27-04356-s001.zip › Supplementary Figures/Supplementary Figure S5. GFAP/KO Stress/20x_GFAP_scalebar.jpg]

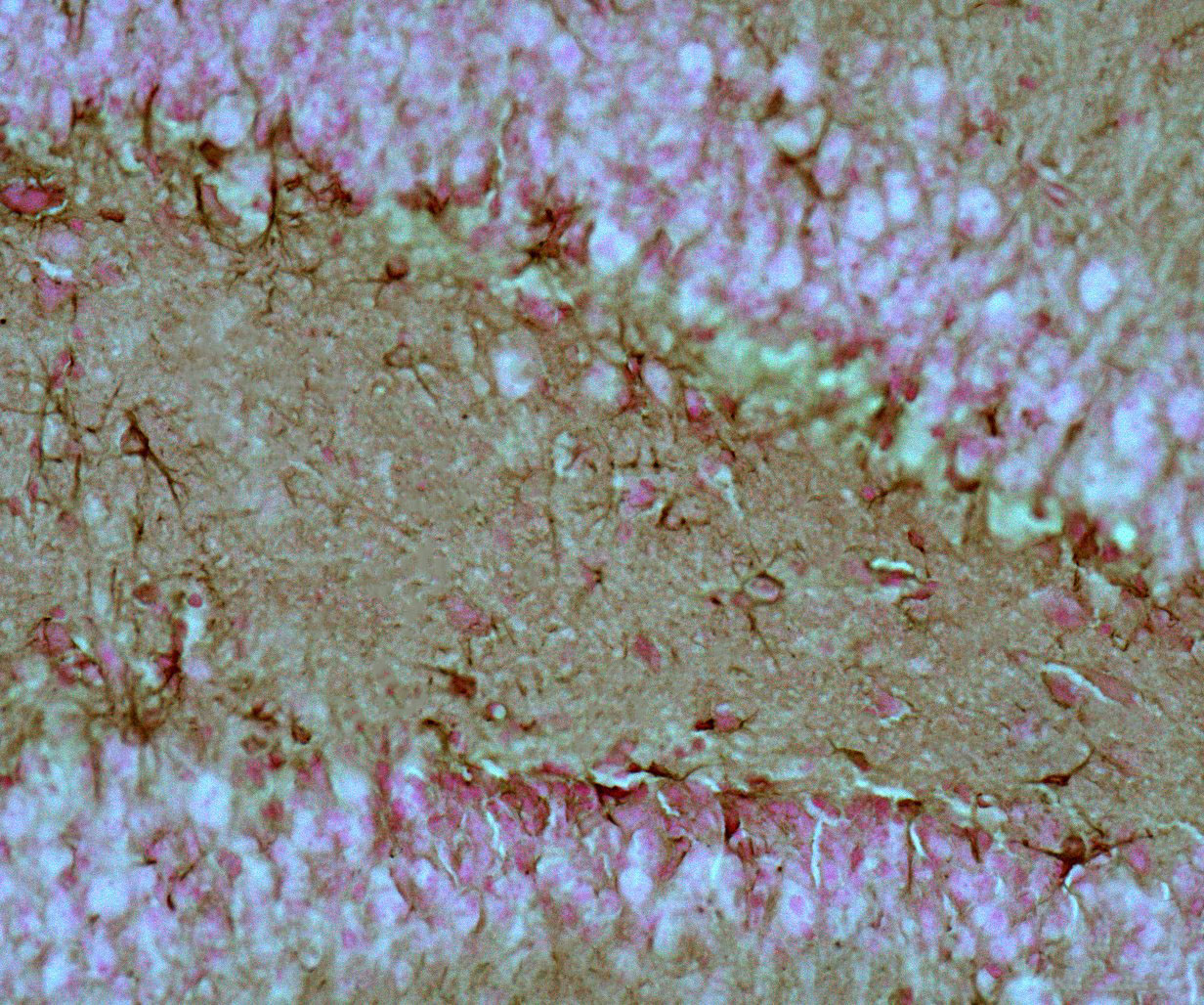

Supplement: Supplementary file 1 [file ijms-27-04356-s001.zip › Supplementary Figures/Supplementary Figure S5. GFAP/WT Control/20x_GFAP.jpg]

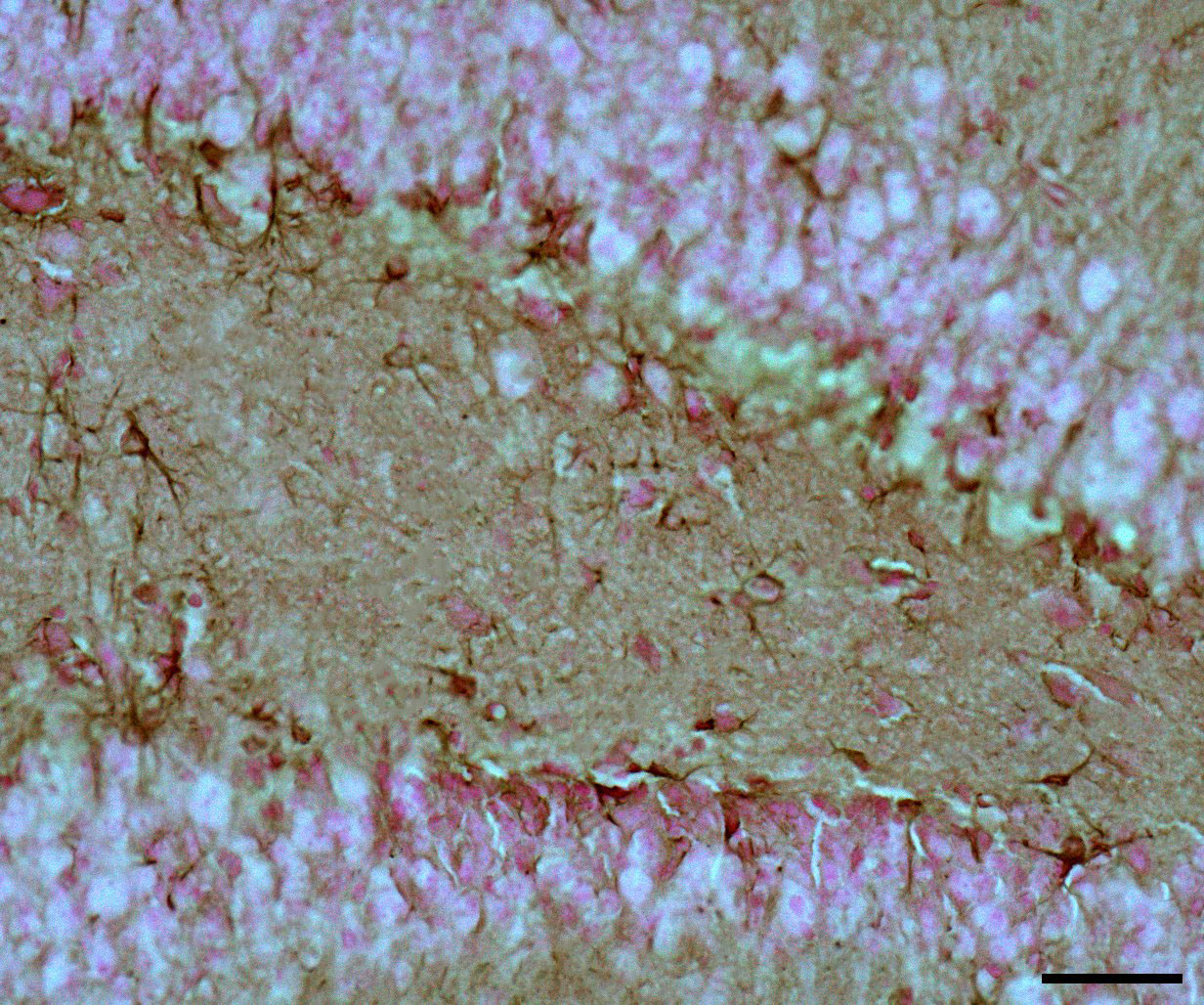

Supplement: Supplementary file 1 [file ijms-27-04356-s001.zip › Supplementary Figures/Supplementary Figure S5. GFAP/WT Control/20x_GFAP_scalebar.jpg]

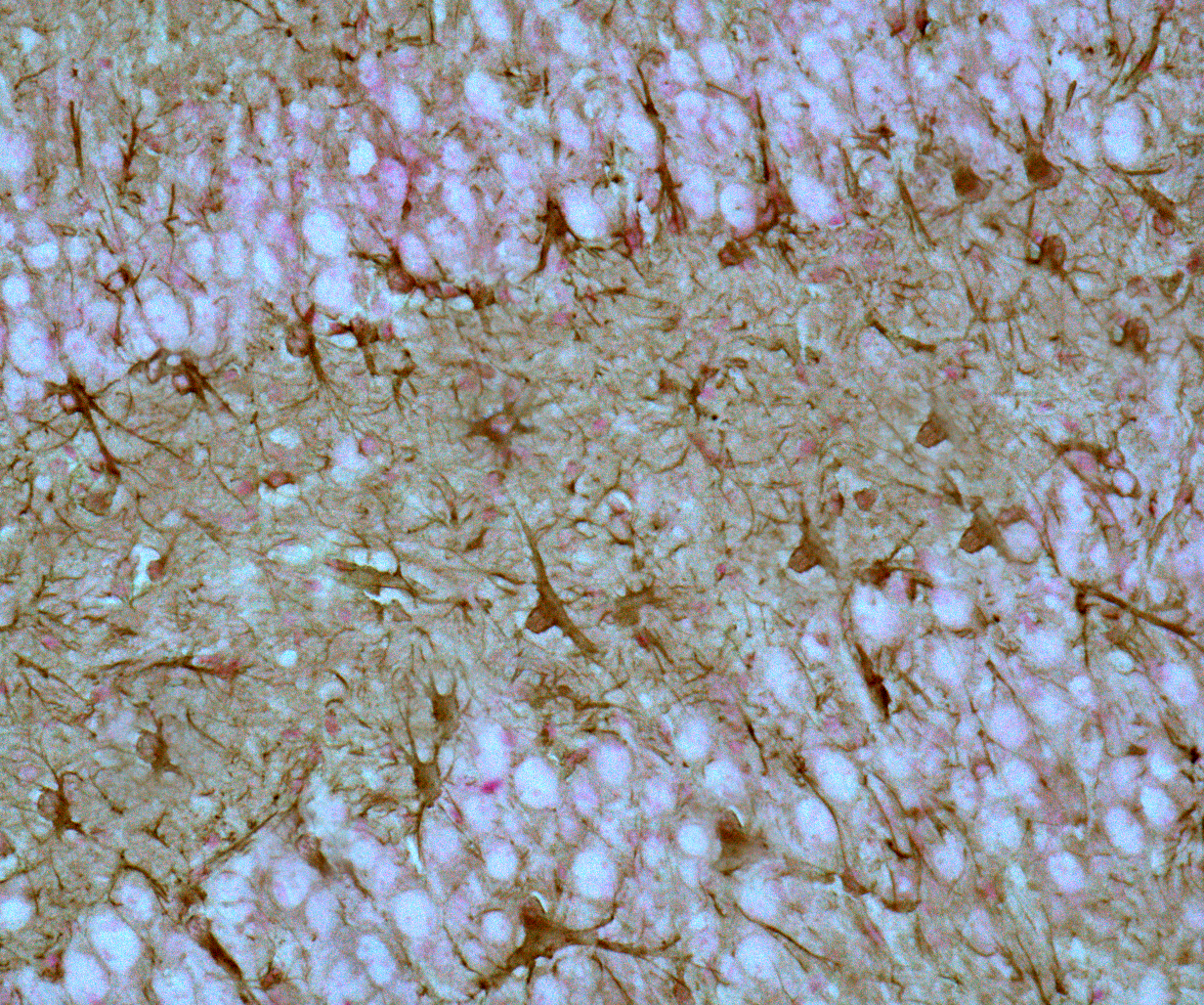

Supplement: Supplementary file 1 [file ijms-27-04356-s001.zip › Supplementary Figures/Supplementary Figure S5. GFAP/WT Stress/20x_GFAP.tif]

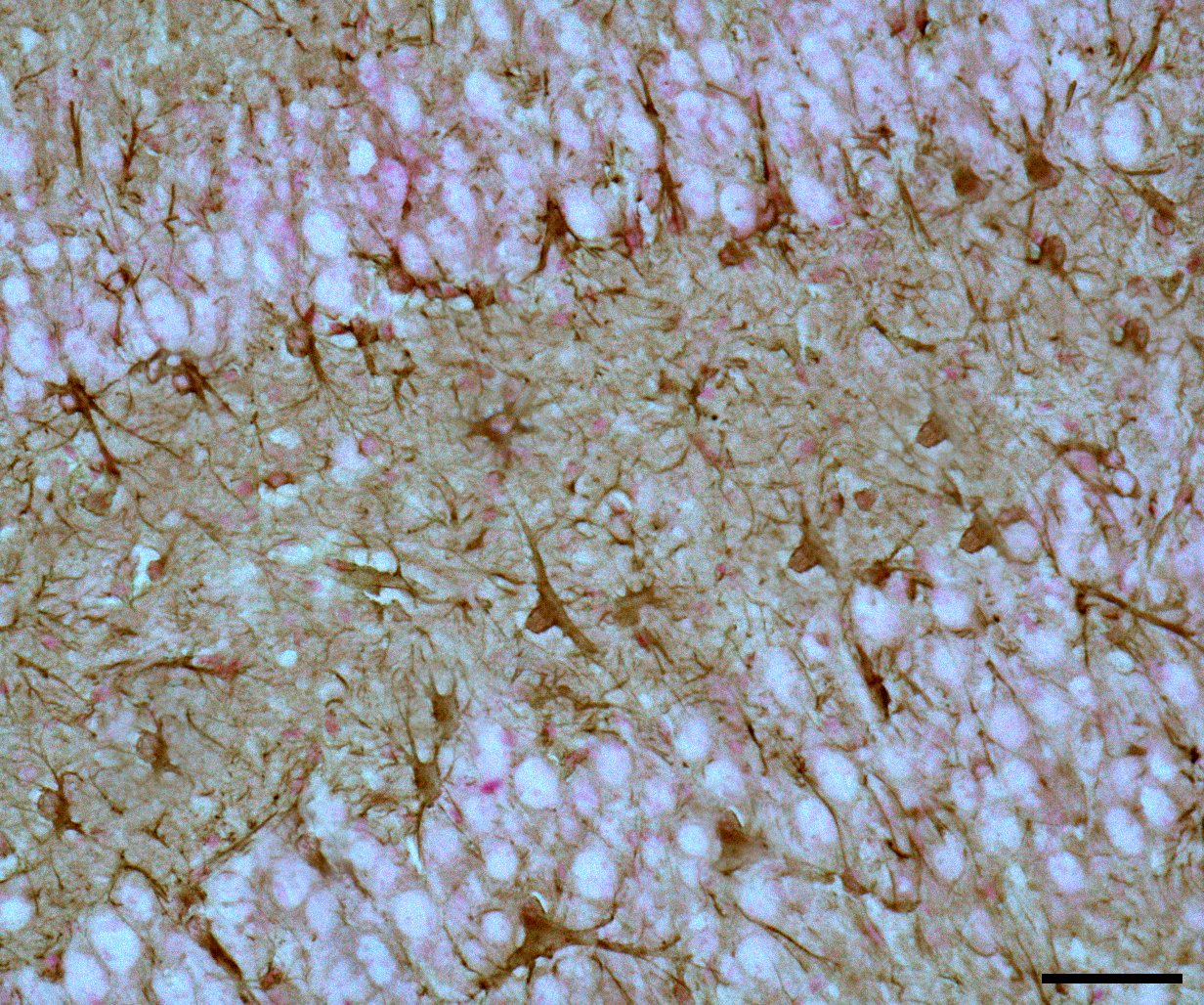

Supplement: Supplementary file 1 [file ijms-27-04356-s001.zip › Supplementary Figures/Supplementary Figure S5. GFAP/WT Stress/20x_GFAP_scalebar.jpg]
